# Genomic analysis of *Kazachstania aerobia* and *Kazachstania servazzii* reveals duplication of genes related to acetate ester production

**DOI:** 10.1099/mgen.0.001029

**Published:** 2023-06-05

**Authors:** Mandy Man-Hsi Lin, Michelle E. Walker, Vladimir Jiranek, Krista M. Sumby

**Affiliations:** ^1^​ Department of Wine Science, School of Agriculture, Food & Wine, The University of Adelaide, Waite Campus, South Australia, 5064, Australia; ^2^​ Australian Research Council Training Centre for Innovative Wine Production, Glen Osmond, South Australia, 5064, Australia

**Keywords:** PacBio, whole genome sequencing, *Kazachstania aerobia*, *Kazachstania servazzii*, ester genes, wine aroma

## Abstract

*Kazachstania aerobia* and *Kazachstania servazzii* can affect wine aroma by increasing acetate ester concentrations, most remarkably phenylethyl acetate and isoamyl acetate. The genetic basis of this is unknown, there being little to no sequence data available on the genome architecture. We report for the first time the near-complete genome sequence of the two species using long-read (PacBio) sequencing (*K. aerobia* 20 contigs, one scaffold; and *K. servazzii* 22 contigs, one scaffold). The annotated genomes of *K. aerobia* (12.5 Mb) and *K. servazzii* (12.3 Mb) were compared to *Saccharomyces cerevisiae* genomes (laboratory strain S288C and wine strain EC1118). Whilst a comparison of the two *Kazachstania* spp. genomes revealed few differences between them, divergence was evident in relation to the genes involved in ester biosynthesis, for which gene duplications or absences were apparent. The annotations of these genomes are valuable resources for future research into the evolutionary biology of *Kazachstania* and other yeast species (comparative genomics) as well as understanding the metabolic processes associated with alcoholic fermentation and the production of secondary ‘aromatic’ metabolites (transcriptomics, proteomics and metabolomics).

## Data Summary

All sequencing data generated in this study are available in the National Center for Biotechnology Information under BioSample SAMN25820612 (genome; PF_8_W29) and SAMN25820613 (genome; PF_9_W20), as part of the NCBI BioProject accession number PRJNA799447. Genomic data used in this study can be retrieved from NCBI genome accession numbers JAKOOU000000000 and JAKOOT000000000 (Table 1). Genome sequences of the reference *S. cerevisiae* strains S288C and EC1118 were downloaded from NCBI and *Saccharomyces* Genome Database (SGD, https://www.yeastgenome.org). All software used in the analyses of the genome sequences, except for Geneious, are publicly available, and the sources have been provided in the article. Figshare DOI: https://doi.org/10.6084/m9.figshare.22339312.v1 [1].

**Impact Statement**

*Kazachstania aerobia* and *Kazachstania servazzii* are members of the genus *Kazachstania*. Only two members of the genus are fully sequenced – *K. africana* and *K. naganishii*, together with closely related species in the family *Saccharomycetaceae* (*Naumovozyma castellii* and *Nauvomozyma dairenensis*). In this study, the genomes of *K. aerobia* (PF_8_W29) and *K. servazzii* (PF_9_W20) were sequenced and assembled as 20 contigs, one scaffold and 22 contigs, one scaffold respectively including the mitochondrial genome. Gene orthologues were identified by sequence comparison to *Saccharomyces cerevisiae* for proteins related to ester production. The orthologues from all six species were compared for sequence similarities to identify any gene divergence between these members of the family *Saccharomycetaceae*. The results provide valuable insight into species-specific variation in ester formation during fermentation.

## Introduction

Yeasts play an essential role in the fermentation of alcoholic beverages transforming sugars to ethanol, carbon dioxide and other metabolites [2–4]. Many of these secondary metabolites contribute to the unique aroma and flavour of fermented beverages. *Saccharomyces cerevisiae*, the primary yeast involved in alcohol fermentation, has received wide attention in research and in the beverage (and food) industry due to its fast growth rate and ability to complete fermentation. Whilst inoculation with *S. cerevisiae* starters is considered to lower the risk of off-flavours or stuck fermentation [5], these strains (with a few exceptions) do not contribute significantly to the sensory properties of the final product, which often lacks complexity [6]. The demand for new wine styles, greater complexity and reduced alcohol content has led to bioprospecting for novel yeasts capable of enhancing beverage flavour or limiting alcohol content [7, 8]. Non-*Saccharomyces* yeasts, once considered undesirable as a source of spoilage, are of particular interest as potential wine starters, due to their ability to secrete enzymes (e.g. β-glucosidase to release glycosidically bound aroma compounds), reduce ethanol concentration and produce secondary metabolites such as esters [9, 10]. Their sensitivity to ethanol necessitates that non-*Saccharomyces* yeasts be used in mixed- or co-culture fermentations with *S. cerevisiae*, allowing for complete sugar utilization as well as modulation of positive volatile compounds [11]. In addition, some species have antimicrobial activity towards wine spoilage organisms [12], which also lends these yeasts to potential use as starter cultures to preserve (as a bioprotectant [13, 14]) and improve the sensory quality of wine and beers [15].

Whilst the application of non-*Saccharomyces* in wine production is becoming more common [16], the intense focus on the genetics and the physiology of these organisms is more recent. High-throughput whole genome sequencing [17] has led to the repository of mostly draft genomes [18–20], with only a few complete assemblies to allow the prediction of functional genes, gene annotation and genome architecture [21–23]. Additionally, recent studies have reported on specific flavour gene duplications and the absence of genes putatively involved in ester production in the non-*Saccharomyces* yeast species *Hanseniaspora uvarum*, *Hanseniaspora osmophila* and *Hanseniaspora vineae* [24, 25]. The increasing availability of these genome sequences and others is important to understand the genomic and metabolic features of non-*Saccharomyces* yeasts in relation to the fermentation of foods and beverages.

*Kazachstania* is a non-*Saccharomyces* yeast genus belonging to the family *Saccharomycetaceae* [26, 27]. In 1971, Zubkova first proposed the genus *Kazachstania* with the description of *Kazachstania viticola*, which was first isolated in Kazakhstan from fermenting grapes [28]. It was later considered to be a synonym of *Saccharomyces dairenensis* [29]. In 2003, several species belonging to *Arxiozyma*, *Kluyveromyces*, *Pachytichospora* and *Saccharomyces* (*sensu lato*) were reassigned and reclassified into the genus *Kazachstania* [26] based on multigene sequence analysis of the ‘*Saccharomyces* complex’ (where ~80 species were grouped into 14 clades). Phylogenetic analysis using the D1/D2 LSU rRNA gene sequences has led to the inclusion of over 40 species in this genus [27], with numbers continuing to increase. To date, several species of this genus (including *Kazachstania aerobia*, *K. gamospora* and *K. servazzii*) are reported to produce high amounts of floral and fruity compounds in white and red wines following sequential fermentation with *S. cerevisiae* [30–33].

The genus *Kazachstania* is phylogenetically diverse [26, 27, 34–37]; however, the lack of a fully sequenced reference genome makes useful assembly and annotation arduous. Data on the genetic features and physiological properties of the genus *Kazachstania* are scarce in comparison to its closest relative, *S. cerevisiae*, which is well characterized as a ‘model’ eukaryote. Whilst the *Kazachstania africana* and *K. naganishii* genomes have been fully sequenced with properties such as protein coding genes and genome size reported [21, 22], the genomes are not fully annotated, with protein functions undefined. With regard to *K. servazzii*, the mitochondrial genome (30.8 kb) was initially reported by Langkjær *et al*. [38] from a soil isolate (strain CBS4311; NCBI BioProject accession no.: PRJNA12156). To date, only four draft (incomplete) genomes have been made publicly available: two isolates from kimchi strain CBA6004 [36 contigs (12.5 Mb); NCBI BioProject accession no.: PRJNA434537], and strain SRCM102023, [91 contigs (12.8 Mb); NCBI BioProject accession no.: PRJNA390859 [39]], and the soil isolates UCD13 (12 Mb) and UCD335 (11.8 Mb) (assembled at scaffold level, both under NCBI BioProject accession no.: PRJNA564535) [19].

In our previous studies [32, 33], we explored the fermentative traits and characteristics of *K. aerobia* and *K. servazzii* isolates in both sterile and non-sterile red and white wines. Wines fermented with *Kazachstania* spp. were chemically and sensorially distinct from those that were fermented with *S. cerevisiae* alone. Whilst further evaluation is required in winery-scale fermentations, these species appear to be ideal as potential starter cultures partnered with *S. cerevisiae* as they produce high levels of acetate esters, such as 2-phenylethyl acetate and isoamyl acetate [40, 41]. Sensory analysis of Shiraz wines showed that these compounds were perceived as jammy and fruity flavours when compared with the *S. cerevisiae* fermented wines [33]. Other non-*Saccharomyces* species associated with increased levels of 2-phenylethyl acetate in wines include *Hanseniaspora guillermondii* and *H. osmophila* [42, 43]. Additionally, *Wickerhamomyces anomalus* (previously known as *Pichia anomala*) and *H. guillermondii* increased isoamyl acetate concentrations in mixed fermentations [42]. More recently, the increased formation of 2-phenylethyl acetate in *H. vineae* was suggested to be caused by gene duplication of the aromatic amino acid aminotransferases (*ARO8* and *ARO9*) and phenylpyruvate decarboxylase (*ARO10*) [24].

The first genes identified in acetate ester synthesis were the alcohol acetyltransferases (AATases) catalysing the formation of esters from acetyl coenzyme A (CoA) and their corresponding alcohols [44]. During alcoholic fermentation AATase activity of yeasts produce many important aroma compounds including phenylethyl acetate, which is described as a floral aroma that is reminiscent of roses [45]. The alcohol acetyltransferase encoding genes, *ATF1* and *ATF2* (paralogue of *ATF1*), are responsible for the majority of acetate ester biosynthesis in *S. cerevisiae* [46]. Overexpression of *ATF1* in *S. cerevisiae* results in significantly increased ester production and when constitutively expressed in three commercial wine yeasts, the levels of acetate esters increased, with 2-phenylethyl acetate being 2- to 10-fold higher than the wild-type [47]. The double deletion of *ATF1* and *ATF2* in *S. cerevisiae* results in the inability to form isoamyl acetate, although 2-phenylethyl acetate is still produced, albeit in reduced amounts (11 % of the parent strain) [46]. Additional information on AATase and ester synthesis in *S. cerevisiae* can be found in the review by Sumby *et al*. [48]. More recently AATase orthologues have also been identified in several non-*Saccharomyces* species including *Candida glabrata*, *Kluyveromyces lactis*, *Lachancea waltii* [49] and *Hanseniaspora vineae* [24]. Each species has a single AATase orthologue, which is similar to *S. cerevisiae* Atf2 based on the pairwise alignment of AATase orthologous amino acid sequences [49].

**Fig. 1.**
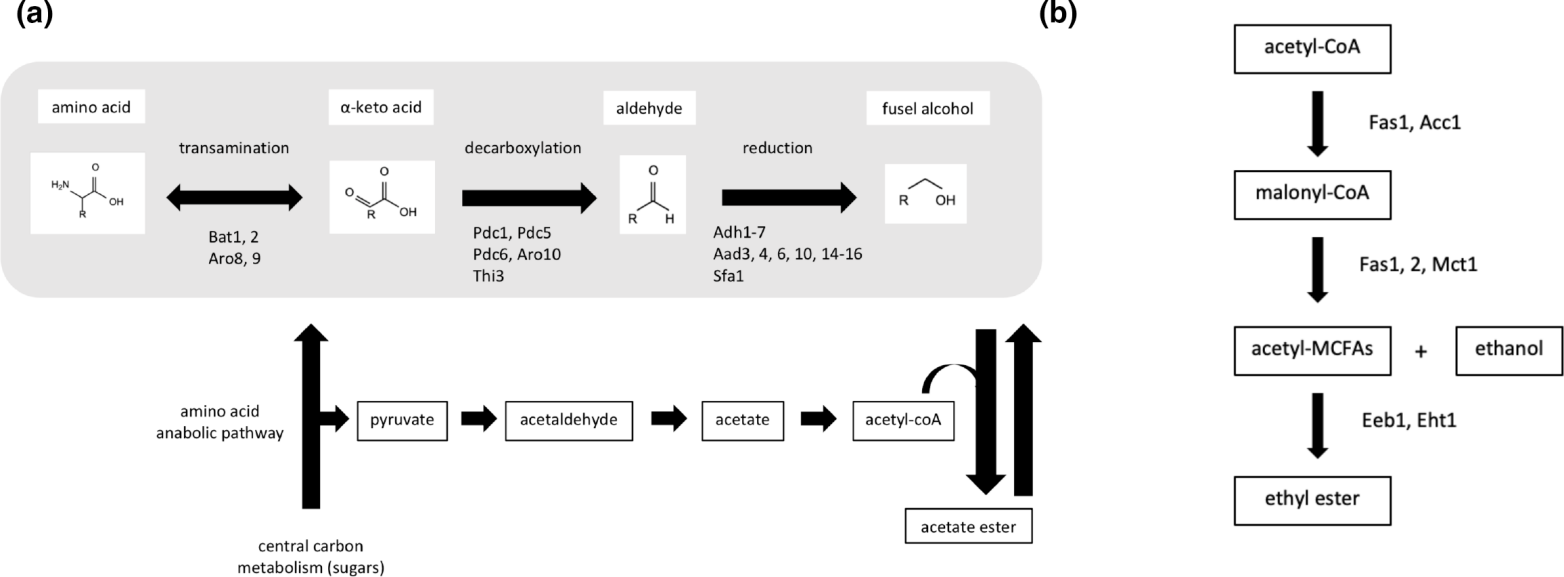
(a) Enzymes involved in ester production in *S. cerevisiae* and (b) enzymes involved in the synthesis of fatty acid ethyl esters. The Ehrlich pathway reactions are shaded grey. Once fusel (higher) alcohols are formed, they can be esterified to the corresponding esters (acetates).

Focusing on 2-phenylethyl acetate and isoamyl acetate, two pathways lead to their synthesis in *S. cerevisiae* during fermentation (Fig. 1a): *de novo* synthesis from sugar substrates or the catabolism of branch amino acids. The latter is via the Ehrlich pathway to form fusel alcohols which are then esterified by alcohol acetyltransferase (Fig. 1a). In the case of 2-phenylethyl acetate, l-phenylalanine is the precursor to phenylethyl alcohol, whilst isoleucine and valine are precursors to amyl alcohol and isobutanol [50]. The first and the second steps of the Ehrlich pathway are catalysed by amino acid transaminases (Aro8, Aro9, Bat1 and Bat2) and thiamine pyrophosphate (TPP)-dependent decarboxylases (Aro10, Pdc1, Pdc5, Pdc6 and Thi3) [50, 51]. The final step of the Ehrlich pathway (higher alcohol formation) may be catalysed by any of the alcohol dehydrogenases (Adh1–5) or by Sfa1 (a formaldehyde dehydrogenase) [52].

The high levels of acetate ester production (nominally; phenylethyl acetate and isoamyl acetate) by *Kazachstania* spp. could be explained in two ways: (1) species-specific differences in the genes involved in aroma formation, or (2) resistance or lack of negative feedback by high levels of phenylethyl acetate and isoamyl acetate. For example, yeasts that are resistant to toxic analogues of phenylalanine show increased production of aromatic alcohols and their corresponding esters [53–55]. Researchers have used toxic analogues of phenylalanine to select for yeast with mutations that increase phenylalanine metabolism, enhancing the production of 2-phenyl ethanol and 2-phenylethyl acetate during saké production [54, 55]. Yeasts that are resistant to toxic analogues of phenylalanine, such as *o*-fluoro-dl-phenylalanine or *p*-fluoro-dl-phenylalanine, displayed changes in the action of phenylalanine-dependent 3-deoxy-d-arabino-heptulosonate-7 phosphate (DAHP) synthase [54], which catalyses the first step in the production of aromatic amino acids (tryptophan, tyrosine and phenylalanine) via the Shikimate pathway.

DAHP synthase is known to be encoded by two genes, *ARO3* and *ARO4* [56]. *ARO3* is regulated through feedback inhibition by phenylalanine with the GCN4 activator protein implicated in both the activation and the basal control of *ARO3* [56]. More recent efforts to understand the genes involved in the production of phenylethyl acetate have focused on quantitative trait locus (QTL) analysis [57]. Four QTLs responsible for high 2-phenylethyl acetate (2-PEA) production were identified, two of which were linked to the parental genomes and further investigated for causative gene mutations. *FAS2*, encoding the alpha subunit of the fatty acid synthetase complex, and *TOR1*, a PIK-related protein kinase and rapamycin target, involved in nitrogen regulation, were identified and the mutations tested in relation to 2-PEA production [57]. CRISPR-Cas9-mediated allele exchange of the superior alleles of *TOR1* and *FAS2* in the parent strain increased 2-PEA production by 70 % [57].

Other major genes involved in ester biosynthesis in *S. cerevisiae* include the paralogous genes *EEB1* and *EHT1* [58], which encode an acyl-CoA: ethanol *O*-acyltransferase [an enzyme required to produce medium-chain fatty acid (MCFA) ethyl esters, such as ethyl hexanoate, ethyl octanoate, ethyl decanoate, Fig. 1b]. Ethyl hexanoate, which imparts a fruity flavour (apple-like aroma) in alcoholic beverages is formed by an enzyme-catalysed condensation reaction of hexanoic acid and ethanol. Formation of ethyl hexanoate is dependent upon the substrate concentrations and enzymatic activity, as the Eht1 enzyme has the largest contribution to the formation of MCFA ethyl esters [59]. In *Komagataella phaffii* (previously known as *Pichia pastoris*), the esterase activity of *EHT1* knockout and overexpression strains was either significantly lower or higher, respectively, which demonstrates the importance of *EHT1* in regulating esterase activity in fermentation products [59]. The major esterase, isoamyl acetate-hydrolysing esterase encoded by *IAH1* (YOR126C), has been cloned and characterized in *S. cerevisiae* [60]. Fukuda *et al*. [60] reported a decrease in the production of isoamyl acetate (banana aroma) in saké by disrupting the *IAH1* and overexpressing the *ATF1* genes, and concluded that isoamyl acetate accumulation was dependent on the ratio of the esterase and alcohol acetyltransferase activities. More recently, the alcohol transferase Eat1 was reported to be responsible for bulk ethyl acetate production in *Cyberlindnera fabianii*, *Kluyveromyces lactis*, *Kluyveromyces marxianus* and *Wickerhamomyces anomalus* [61, 62]. Two putative homologues were later identified in *S. cerevisiae* (*EAT1* and *IMO32*), with evidence that *EAT1* is responsible for 50 % of ethyl acetate production [62]. Interestingly, Eat1 differs from the other AATases which are cytosolic proteins, as found in the mitochondria [63]; the coding region is predicted to have a mitochondrial targeting sequence. The mechanism that promotes AATase activity in Eat1 remains elusive, as to date there is no crystal structure of the enzyme and its acetyl-CoA intermediate to study this.

In this study, we sequenced the genomes of two *Kazachstania* spp. isolates, with the aim of providing insight into the genomic and metabolic features of *K. aerobia* and *K. servazzii* using data that are readily available on the Basic Local Alignment Search Tool (blast; https://blast.ncbi.nlm.nih.gov/Blast.cgi) database and the *Saccharomyces* Genome Database (SGD; https://www.yeastgenome.org/). We present the *de novo* sequences and assembly (at contig level) of both isolates using PacBio technology, as well as the analysis of orthologous genes responsible for flavour compounds. Sequencing of wine yeast genomes is the first step towards understanding the genetic differences and phenotypic variation between the different *Kazachstania* species suited to winemaking.

## Methods

### Yeast isolates

Isolates used in this study (Table 1) were grown in YEPD (1 % yeast extract, 2 % bactopeptone, 2 % glucose) for 24 h at 28 °C from glycerol stocks.

**Table 1.** *Kazachstania* spp. isolates used in this study; NCBI GenBank accession numbers (ITS sequences) were obtained in a previous study [32]

Species (isolate)	NCBI GenBank accession no. (ITS)	NCBI genome accession no.	NCBI BioSample accession no.	NCBI BioProject accession no.
K. aerobia (PF_8_W29)	MN328365	JAKOOU000000000	SAMN25820612	PRJNA799447
K. servazzii (PF_9_W20)	MN328373	JAKOOT000000000	SAMN25820613	PRJNA799447

### DNA extraction

Genomic DNA (gDNA) was extracted using a Qiagen Genomic-tip 100 G^−1^ kit (cat. no. 10243; Qiagen) according to the manufacturer’s instructions for yeast, with minor modifications. Zymolyase 20T (MP Biomedicals; 1000 U ml^−1^ in distilled water) was increased to 500 µL and added to *Kazachstania* cells (~ 2×10^9^). The yield and concentration of the eluted DNA was assessed via a NanoDrop One spectrophotometer (Thermo Fisher Scientific). DNA purity and integrity were based on calculation of the *A*_260_/*A*_280_ and *A*_260_/*A*_230_ ratios, as well as visually after electrophoresis (0.75 % agarose in tris acetate EDTA (TAE) buffer). gDNA samples with an *A*_260_/*A*_280_ ratio of 1.8–2.0 [7.5 μg (413.9 ng µl^−1^; PF_8_W29 and 56.2 ng µl^−1^; PF_9_W20) for each sample] were submitted to the South Australian Genomics Centre (SAGC) (Adelaide, Australia) for PacBio sequencing.

### Library preparation and PacBio sequencing

Library preparation and sequencing were performed by the Central Analytical Research Facility (CARF) at the Queensland University of Technology, Australia (subcontracted by SAGC). gDNA samples were checked for sugars (*N*-acetyl-d-glucosamine) by HPLC using a Shodex OHpak SB-806M HQ (8.0 mm I.D.×300 mm) column (https://www.shodex.com/en/dc/03/06/05.html#!), because chitosan, a carbohydrate found in certain fungi, crustaceans and insects, can inhibit PacBio sequencing and reduce the number and quality of the reads. In total, 1 µg of each gDNA sample was sheared using a Covaris g-Tube to produce sheared library sizes of 9.5–10 kb. Femto Pulse (Agilent Technologies) was used to confirm the size fragments and concentrations were measured on a Qubit 4.0 fluorometer (Thermo Fisher Scientific). Libraries were then prepared following the protocol in the PacBio Procedure and Checklist – Preparing Multiplexed Microbial Libraries Using SMRTbell Express Template Prep Kit 2.0 (Pacific Biosciences), with double standard volumes throughout the procedure due to having a 2-plex library. Beads were used to remove <3 kb SMRTbell templates and the final library size and concentration was measured by Femto Pulse and Qubit for equimolar pooling.

The PacBio Sequel Binding kit 3.0 was used to bind prepared DNA libraries to the Sequel I system, with the calculations obtained from SMRT Link software (v8.0), sequencing primer v4, 1 h polymerase binding time and 1.2× AMPure PB beads complex clean-up. Libraries were sequenced on a SMRT Cell 1M v3 LR for 20 h in Continuous Long Read (CLR) mode with 2 h of pre-extension, as recommended by PacBio for *De Novo* Assembly – Microbial Multiplexing.

### *De novo* genome assembly, annotation and gene orthology analysis (flavour compounds)

*De novo* genome assembly was performed by SAGC following the following procedures: the raw sequences [subreads.bam, in PacBio BAM files, from zero-mode waveguide (ZMW) hole)] were split into BAM files by sample which includes demultiplexing of barcoded data by using Lima (v.2.0.0). The demultiplexed reads were then converted to CCS/HiFi reads with a minimum predicted accuracy read of 0.99 (default) using pbccs (v6.0.0), and finally converted to fasta format using bam2fastx (v1.3.1). The long read assembler Flye (v2.8.3) was used to perform *de novo* assembly on the CCS reads with the --pacbio-hifi command line argument.

Gene features were annotated in the genome sequences using *S. cerevisiae* S288C as the reference genome. Protein coding gene models were predicted using both AUGUSTUS (ver. 3.4.0) [64] and the Yeast Genome Annotation Pipeline (YGAP) [65]. For homology-based prediction of transcripts/genes, the S288C ORF were downloaded from the SGD (https://www.yeastgenome.org/) and Geneious Prime (ver. 2021.0.3) was employed to align the annotated ORFs with the hypothetical protein ORFs. Protein sequences were functionally assigned using InterPro (version 87.0) (https://www.ebi.ac.uk/interpro/). A protein blast (BLASTp) analysis search (E-value ≤0.01, gapped alignments, W value=3) was performed with amino acid sequences of *K. aerobia* and *K. servazzii*, which resulted in the best hit with the two members of the genus *Kazachstania* (*K. africana*, *K. naganishii*) as well as those closely related species in the family *Saccharomycetaceae* (*Naumovozyma castellii* and *Naumovozyma dairenensis*).

Amino acid sequences of orthologues were used to generate a multiple sequence alignment with Clustal Omega ([66]; https://www.ebi.ac.uk/Tools/msa/clustalo), in order to find conserved regions and important sequences. Orthologous relationships with *S. cerevisiae* strain S288C and the wine strain EC1118 sequences were analysed on OrthoVenn2 (https://orthovenn2.bioinfotoolkits.net/home) [67]. OrthoFinder (v.2.5.4) was also used to detect orthologous groups, as well as to identify duplicate genes when compared to both *S. cerevisiae* strains (https://github.com/davidemms/OrthoFinder) [68].

## Results

### High-quality *de novo* sequencing and genome assemblies of *K. aerobia* and *K. servazzii*

High-quality genome assemblies for *K. aerobia* PF_8_W29 and *K. servazzii* PF_9_W20 were generated from the PacBio Sequel I platform. A total of 16.45 Gb of raw reads was generated for both isolates, which was subsequently demultiplexed. Of the initial 612 011 productive ZMWs, 40.9 % (250 078) contained reads with one or two barcodes used for the isolates. Following the demultiplexing step, subreads (≥470× coverage) were collapsed to generate higher accuracy (≥99 % base accuracy) Hi-Fi reads, which were subsequently assembled into 12.5 and 12.3 Mb genomes for *K. aerobia* PF_8_W29 and *K. servazzii* PF_9_W20 respectively. The sequencing results and assembled contigs (and scaffolds) are summarized in Table 2. As the main point of this study was a targeted analysis of orthologous genes responsible for flavour compounds of *K. aerobia* and *K. servazzii*, we are unable to provide any more information on chromosome number or structure. Both *K. aerobia* and *K. servazzii* are post-whole genome duplication (WGD) species and the expected number of chromosomes in such organisms generally ranges between 12 and 16. The genomes of *K. aerobia* (12.5 Mb) and *K. servazzii* (12.3 Mb) were comparable to the previously published genomes for *K. africana* (11.13 Mb [21]) and *K. naganishii* (10.84 Mb) and other members of *Saccharomycetaceae* [22, 23].

**Table 2.** Summary of *K. aerobia* PF_8_W29 and *K. servazzii* PF_9_W20 genome assemblies using the PacBio (Sequel I) platform

Metric	*K. aerobia* PF_8_W29	*K. servazzii* PF_9_W20
Scaffold	20	22
Scaffold N50 (bp)	965 203	981 509
Contigs	21	23
Contig N50 (bp)	890 346	874 116
Maximum contig length (bp)	1 231 885	1 131 086
Mitochondrial genome size (kb)	29.6	29.4
G+C (%)	35.8	34.4
Total length (Mb)	12.5	12.3

### *K. aerobia* and *K. servazzii* genome prediction and annotation

Based on the reference genome of a closely related species and well-annotated *S. cerevisiae* (https://www.yeastgenome.org/), the high-quality *de novo* assembly of *Kazachstania* spp. genomes enabled the prediction of 5425 protein-coding genes for *K. aerobia* PF_8_W29 and 5335 for *K. servazzii* PF_9_W20 using the AUGUSTUS and YGAP programs, of which 4621 and 4550, respectively, were *S. cerevisia*e (S288C) homologues and 804 and 785, respectively, were unique genes. The number of gene annotations is among the highest reported for species of the genus *Kazachstania*, and is only comparable to the annotated *K. africana* and *K. naganishii*, for which 5378 and 5321 protein-coding genes, respectively, were predicted [22].

### Genome comparison (orthologous relationships) between *K. aerobia*, *K. servazzii* and *S. cerevisiae* (S288C and EC1118)

**Fig. 2.**
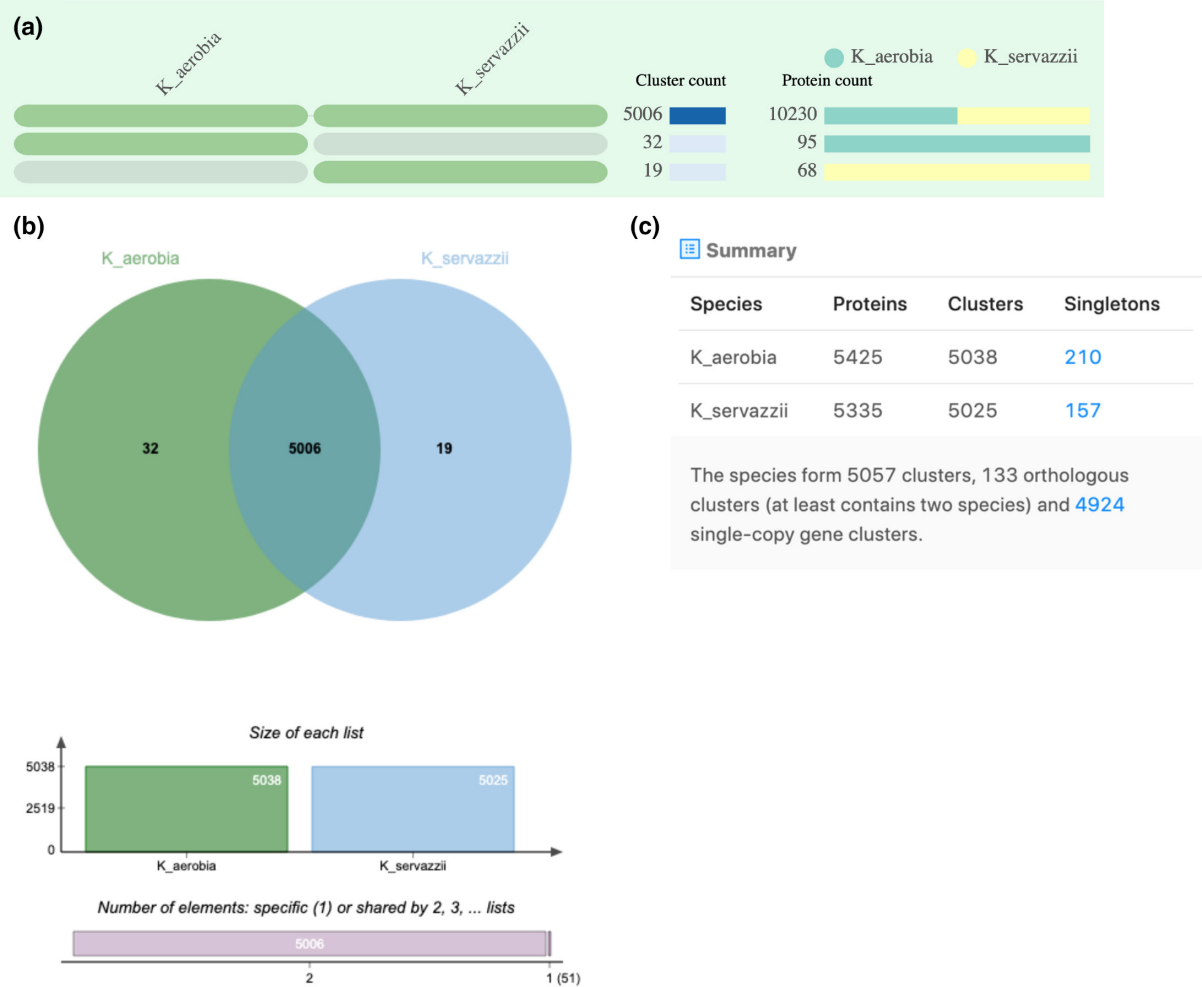
(**a**) Occurrence table indicating shared orthologous group patterns between *K. aerobia* (PF_8_W29) and *K. servazzii* (PF_9_W20). (**b**) Venn diagram displaying the shared orthologous cluster distributions among the species. (**c**) Cluster count in each genome. Singletons describe those genes for which no orthologues can be found in other species.

**Fig. 3.**
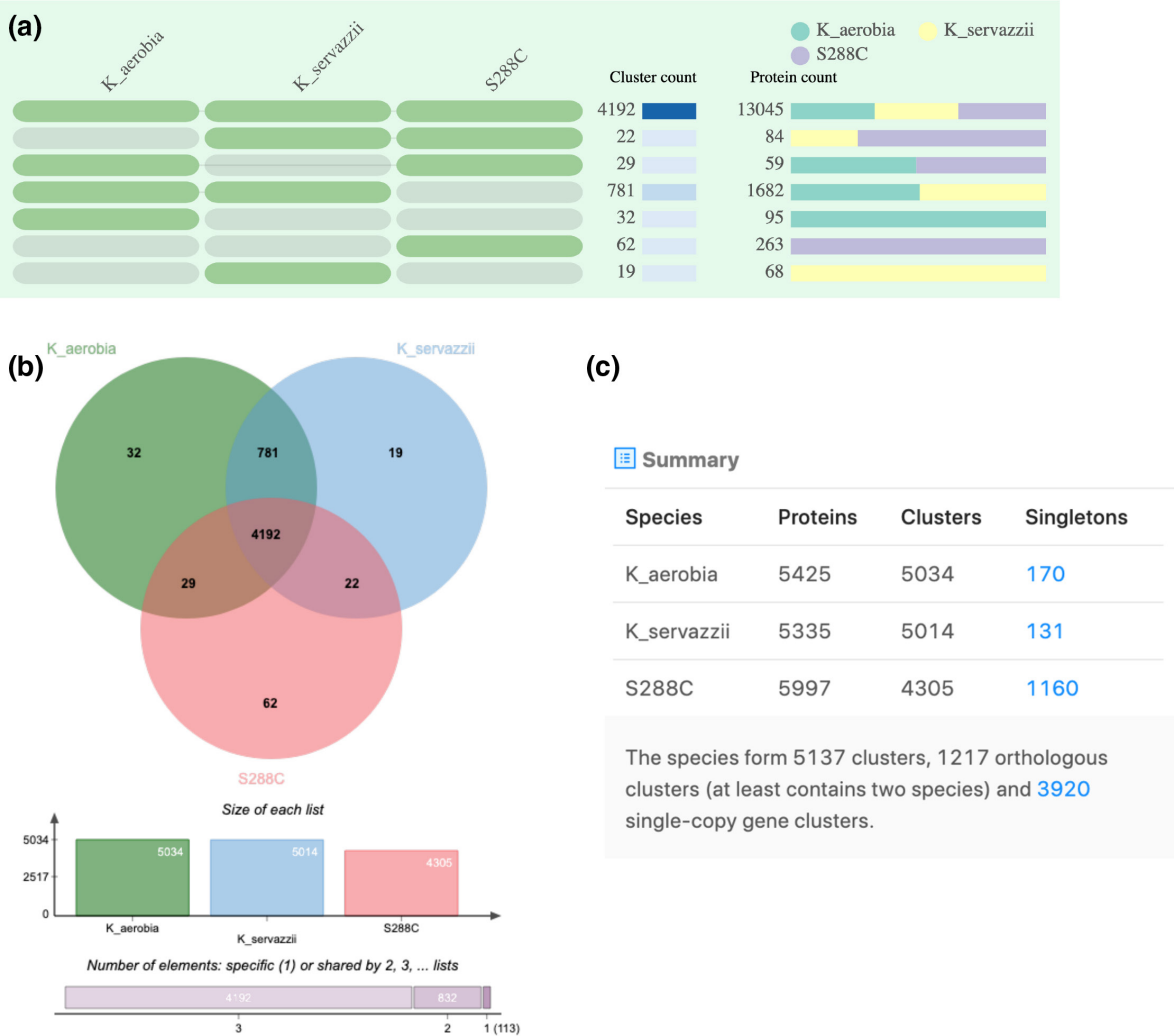
(**a**) Occurrence table indicating shared orthologous group patterns between *K. aerobia* (PF_8_W29), *K. servazzii* (PF_9_W20) and *S. cerevisiae* (S288C). (**b**) Venn diagram displaying the shared orthologous cluster distributions among the species. (**c**) Cluster count in each genome. Singletons describe those genes for which no orthologues can be found in other species.

**Fig. 4.**
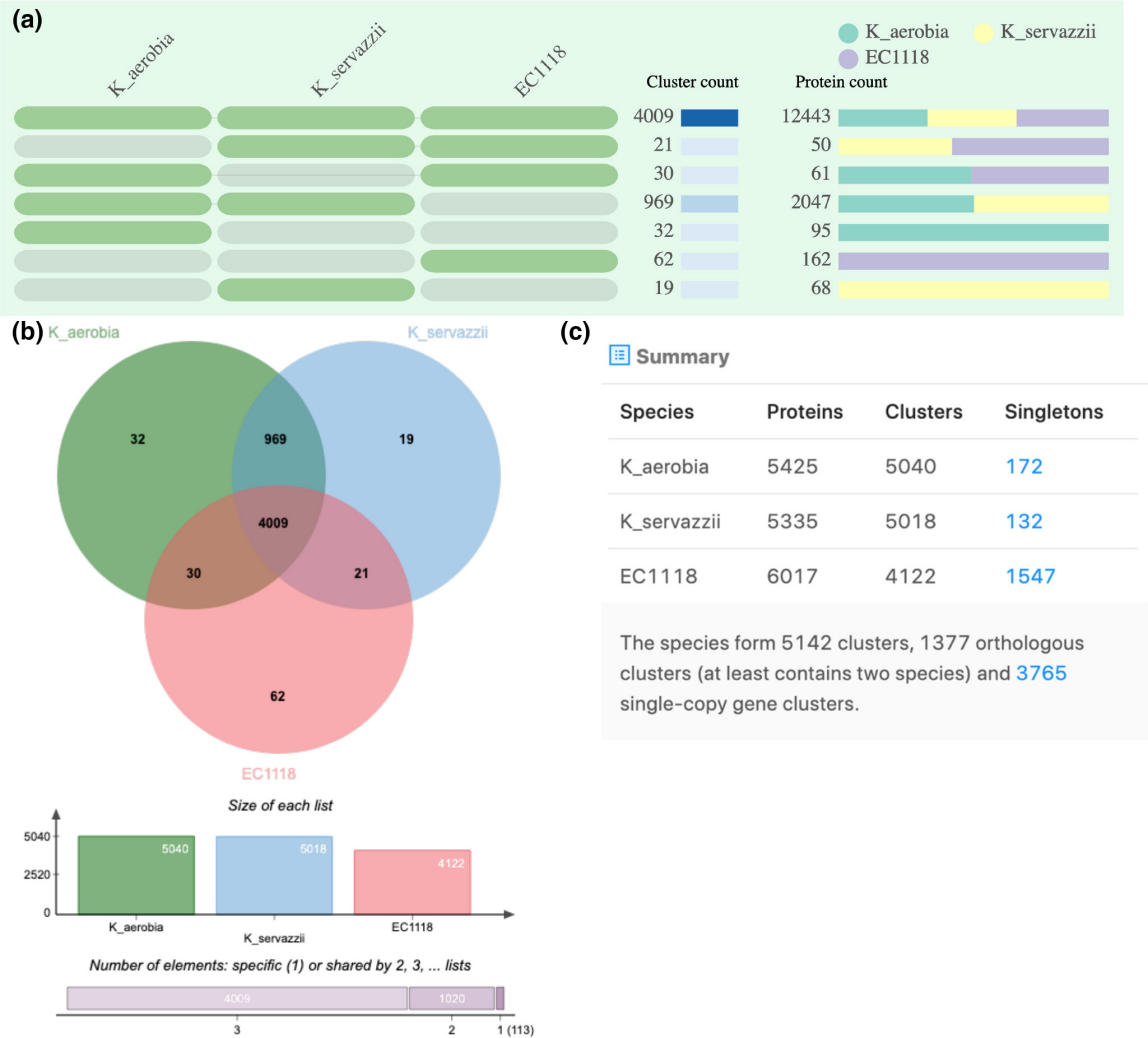
(**a**) Occurrence table indicating shared orthologous group patterns between *K. aerobia* (PF_8_W29), *K. servazzii* (PF_9_W20) and *S. cerevisiae* (EC1118). (**b**) Venn diagram displaying the shared orthologous cluster distributions among the species. (**c**) Cluster count in each genome. Singletons describe those genes for which no orthologues can be found in other species.

The predicted proteome of *K. aerobia* and *K. servazzii* was assigned into orthologous clusters (along with *S. cerevisiae*, S288C and EC1118) in an attempt to identify shared and/or unique characteristics between the species. The OrthoVenn2 web server generated comparison results in tables (Figs 2a–4a) showing the occurrence of cluster groups between species (left), the number of clusters shared between the species (middle) and the number of protein members (protein count) in the shared clusters (right). The OrthoVenn2 software also generated Venn diagrams indicating the number of orthologues shared between the species. In the case of *K. aerobia* (having 5038 clusters), 5006 were shared with *K. servazzii* (5025 total clusters). Thirty-two clusters were unique to *K. aerobia* and 19 clusters to *K. servazzii* (Fig. 2b). When compared to the S288C and EC1118 strains, 4192 clusters were shared between *K. aerobia*, *K. servazzii* and S288C (Fig. 3b), and 4009 clusters were shared between *K. aerobia*, *K. servazzii* and EC1118 (Fig. 4b). There were fewer unique gene clusters identified in *Kazachstania* spp. compared to *S. cerevisiae* (both S288C and EC1118 have 62) (Figs 3b and 4b). The results from OrthoVenn2 (Figs 2c–4c) reflected the genome size differences between the two *Kazachstania* spp. as the predicted 5425 proteins and 5038 clusters in *K. aerobia* was higher than that of *K. servazzii*. Similarly, the two *Saccharomyces* strains varied in protein number, with S288C having 5997 proteins (Fig. 3c) and EC1118 having 6017 proteins (Fig. 4c). The additional 20 proteins probably originate from horizontal gene transfer, as EC1118 has an additional 120 kb sequence not found in S288C [69]. Additionally, the bar plots/graphs revealed the total number of orthologous gene clusters in each species (Figs 2b–4b). The orthologous clusters of *Kazachstania* spp. and *S. cerevisiae* were also annotated, which assigned the clusters to three main gene ontology (GO) categories: (1) biological process, (2) molecular function and (3) cellular component. The GO analysis/functional information associated with each cluster is provided in Table S1, available in the online version of this article. Among the three main categories, GO terms for core orthologous gene clusters were mainly distributed in biological processes in both *Kazachstania* spp., as the most abundant number was associated with enriched biological and metabolic processes (Table S1). For the unique genes found in both *Kazachstania* spp., the majority of GO terms were not assigned to *K. aerobia*, and the majority of GO terms of *K. servazzii* were for helicase activity and SRP-dependent cotranslational protein targeting to membrane (Table S1).

### *In silico* analysis of yeast genes involved in ester (and higher alcohol) biosynthesis

**Fig. 5.**
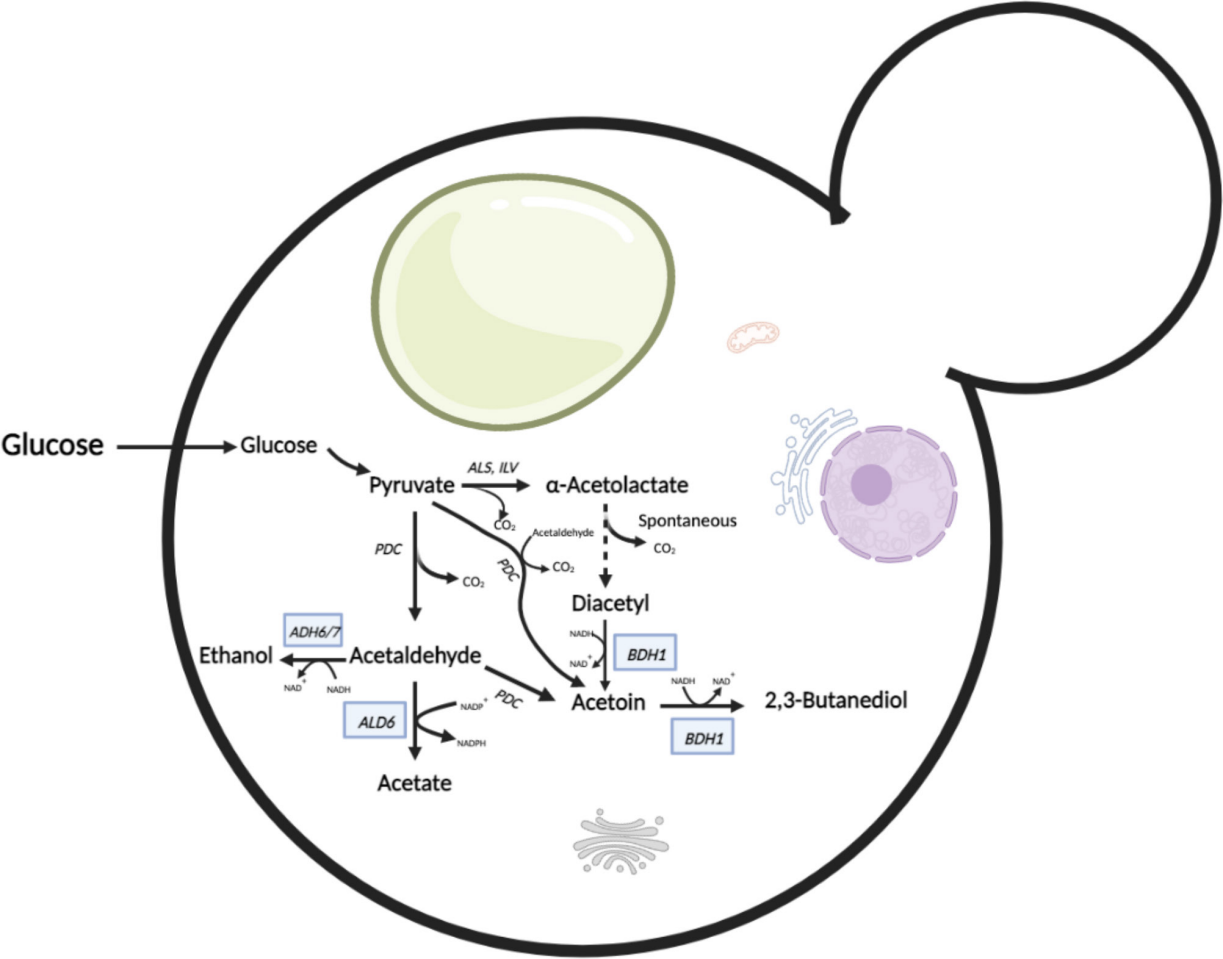
Schematic diagram of metabolic pathways involving putative duplicated genes [*ADH6/7*, *ALD6* and *BDH1* (in blue boxes)] identified in *Kazachstania* spp. (*K. aerobia* and *K. servazzii*) in comparison to *S. cerevisiae* EC1118 (adapted from Ehsani *et al*. [82]). Created with BioRender.com. These duplicated genes are involved in ethanol (alcohol), acetate and 2,3,butanediol synthesis starting from pyruvate.

A list of genes based on *S. cerevisiae* (S288C) was compiled as there was no available information on the genes involved in ester production in *Kazachstania* spp. The gene sequences were used to search for their orthologues in *Kazachstania* spp. (Table 3). All orthologous amino acid sequences (putative proteins) were identified, except for those of Eat1, Adh2 and Adh4 (Tables S2–S17; Figs S1–S16b). Two genes coding for alcohol dehydrogenases (ADHs), *ADH1* and *ADH5*, had similar sequences (Fig. S4b). Additionally, only one orthologue (sequence) could be identified for Eht1 and Eeb1 in *K. aerobia* and *K. servazzii*, which was referred to as Eht1/Eeb1 (Table S3; Fig. S2). When compared to the EC1118 strain, duplicate/repeated genes were found in *Kazachstania* spp. for *ADH6/7*, *ALD6* and *BDH1* (Tables S15–S17, SFigs S14a–S16b). An overview of duplicated genes involved in ester biosynthesis in *K. aerobia* and *K. servazzii* is shown in Fig. 5. No apparent orthologues were identified for *EAT1*, *ADH2* and *ADH4* in *Kazachstania* spp. (Table 3).

**Table 3.** Genes of interest involved in/related to flavour compound biosynthesis (esters and higher alcohols)

Gene name	Related flavour compounds	Major function	Cellular compartment	*K. aerobia* ortholog(ue)	*K. servazzii* ortholog(ue)
*ATF1*	Acetate esters	Alcohol acetyl-CoA transferase	Lipid droplets	contig_21 .g2134	contig_9 .g4697
*ATF2*	Acetate esters	Alcohol acetyl-CoA transferase	Endoplasmic reticulum		
*ARO10*	2-Phenylethanol	Phenylpyruvate decarboxylase	Cytoplasm	contig_3 .g3086	contig_25 .g2549
*ARO3*	2-Phenylethanol	3-Deoxy-d-arabino- heptulosonate-7-phosphase (DAHP) synthase	Cytoplasm	contig_25 .g2765	contig_6 .g4293
*ARO4*	2-Phenylethanol	DAHP synthase	Cytoplasm	contig_14 .g1272	contig_14 .g1042
*ARO7*	2-Phenylethanol	Chorismate mutase	Cytoplasm	contig_25 .g2821	contig_9 .g4733
*EAT1*	Ethyl acetate	Ethanol acetyl-CoA transferase	Mitochondrion	n/a	n/a
*EEB1*	Ethyl hexanoate	Ethanol acyl-CoA transferase	Unknown	contig_30 .g3649	contig_4 .g3533
*EHT1*	Ethyl hexanoate	Ethanol acyl-CoA transferase	Lipid droplets/mitochondrion		
*IAH1*	Acetate esters (isoamyl acetate)	Isoamyl acetate-hydrolysing esterase	Cytoplasm	contig_17 .g1404	contig_17 .g1398
*ADH1*	Higher alcohols	Alcohol dehydrogenase	Cytoplasm	scaffold_15 .g5281	scaffold_20 .g5325
*ADH2*	Higher alcohols	Alcohol dehydrogenase	Cytoplasm	n/a	n/a
*ADH3*	Higher alcohols	Alcohol dehydrogenase	Mitochondrion	contig_25 .g2657	contig_6 .g4185
*ADH4*	Higher alcohols	Alcohol dehydrogenase	Mitochondrion	n/a	n/a
*ADH5*	Higher alcohols	Alcohol dehydrogenase	Cytoplasm/nucleus	Similar to ADH1	Similar to ADH1
ADH6*	Higher alcohols	NADPH-dependent alcohol dehydrogenase	Cytoplasm	contig_3 .g2902 contig_17 .g1558	contig_10 .g24 contig_24 .g2365
ADH7*	Higher alcohols	NADPH-dependent medium-chain alcohol dehydrogenase	Cytoplasm	contig_19 .g1873 contig_21 .g2255	contig_25 .g2372
*BAT1*	Higher alcohols and other aroma compounds	Branched-chain amino acid transferase	Mitochondrion	contig_14.g1311	contig_14.g1081
*BAT2*	Higher alcohols and other aroma compounds	Branched-chain amino acid transferase	Cytoplasm	contig_9.g4737	contig_15.g1327
*FAS2*	Phenylethyl acetate	Fatty acid synthetase	Mitochondrion/cytoplasm	contig_19.g1791	contig_24.g2287
*TOR1*	Phenylethyl acetate	Phosphatidylinositol kinase (PIK)-related protein kinase	Nucleus/cytoplasm	contig_13.g342	contig_12.g604
*GCN4*	Phenylethyl acetate	Transcriptional activator of amino acid biosynthetic genes	Cytoplasm	contig_17.g1561	contig_10.g21
ALD6*	Acetate esters	Acetaldehyde dehydrogenase	Cytoplasm/mitochondrion	scaffold_15.g5207 scaffold_15.g5208	scaffold_20.g5254 scaffold_20.g5255
BDH1*	Higher alcohols	NAD-dependent (*R*,*R*)-butanediol dehydrogenase	Cytoplasm	contig_13.g329 contig_25.g2491 contig_30.g3329	contig_6.g4029

*Duplicate genes which have been found in *Kazachstania* spp. in comparison to the *S. cerevisia*e EC1118 strain. n/a, Not applicable.

The identified putative protein sequences were used as queries in BLASTp, where the highest alignment scores showed similarities with orthologues in closely related species within the family *Saccharomycetaceae* (*S. cerevisiae*, *K. africana, K. naganishii*, *N. castellii* and *N. dairenensis*). The percentages of sequence similarity and identity are shown in Tables S2–S17. Of the AATase family (*ATF1* and *ATF2*), both *K. aerobia* and *K. servazzii* revealed only one AATase orthologue, and only showed similarities with *S. cerevisiae* Atf2 (Table S2). The AATase of *K. aerobia* and *K. servazzii* have 38.98 and 39.96 % amino acid identity with *S. cerevisiae* Atf2, which also showed less homology when compared to orthologues of *Kazachstania* spp. and *Naumovozyma* spp. (Table S2). The rest of the protein sequences displayed relatively similar identities with higher homology across all species (including *S. cerevisiae*), ranging from 50.78 to 88.73 % (Tables S3–S14). Interestingly, no hits were found for the Tor1 (PIK family) orthologue in *N. dairenensis* (Table S14).

The amino acid sequences of the identified flavour (ester and higher alcohol) orthologues were aligned using Clustal Omega with default parameters, and revealed highly conserved regions between *K. aerobia*, *K. servazzii* and *S. cerevisiae* (Figs S1–S12). The *Kazachstania* spp. Atf and the *S. cerevisiae* Atf1 and Atf2 orthologues share two conserved motifs: (1) an H-X-X-X-D catalytic (active) site (*S. cerevisiae* Atf1 residues 19–198) and (2) the WRLICLP region (*S. cerevisiae* Atf1 residues 169–175) (Fig. S1).

## Discussion

Non-*Saccharomyces* yeasts play a substantial role in producing volatile aroma/flavour compounds during winemaking and hence are now being studied with a view towards their potential biotechnological and industrial application. Among these yeasts, several fairly novel species belonging to the genus *Kazachstania* genus are attracting significant interest [27–30] as they can modulate wine aroma profiles through their metabolic activities (particularly ester biosynthesis). However, in contrast to *Saccharomyces*, where most genomics studies are conducted in *S. cerevisiae*, and to a lesser extent other members of the *Saccharomycotina*, there is a lack of gene knowledge related to *Kazachstania* spp. genes. Likewise, from a phenotypic perspective, there are no physiological or morphological traits that can accurately describe the genus *Kazachstania* [70]. In this regard, genomic studies allowing for the linkage of genes to traits would be a valuable resource for future biotechnological application [71].

In this study we present the *de novo* whole-genome sequencing of two *Kazachstania* spp. isolates (*K. aerobia* and *K. servazzii*) from spontaneous Shiraz fermentations from the McLaren Vale region of South Australia [32]. Comparison of the whole-genome assemblies of the two species showed similar numbers/values for genome size (~12.4 Mb), G+C content (~35 %) and the number of predicted genes (average ~5380) (Table 2). These values were expected given the properties of other fully sequenced members of the genus *Kazachstania* [21–23]. In general, the predicted protein-coding genes in *K. aerobia* and *K. servazzii* were also comparable to the those reported by Wolfe *et al*. [22] in *K. africana* and *K. naganishii* (all >5000), with *K. aerobia* displaying the highest number. Comparative analyses of the inferred proteins among the two species showed that there were more unique gene clusters in *K. aerobia* than in *K. servazzii* (Fig. 2). Additionally, when compared with both *S. cerevisiae* strains (S288C and EC1118) *K. aerobia* shared more orthologous gene clusters than *K. servazzii* (Figs 3b and 4b). Lastly, *K. aerobia* exhibited the highest diversity of orthologous gene clusters out of the three species, which could be explained as the result of genetic divergence and domestication events [22, 72] (Figs 3b and 4b).

Amino acid metabolism in yeasts during alcoholic fermentation is responsible for 80 % of flavour-active compounds, as their catabolism leads to the production of higher alcohols which can then be utilized by AATases for the formation of acetate esters [73]. Only one orthologue in the AATase family was found in *Kazachstania* spp., which only had ~38–39 % identity to *S. cerevisiae* Atf2 (Table S2). As mentioned earlier, *S. cerevisiae* AATase is encoded by two genes, as opposed to the distantly related yeast species *C. glabrata*, *K. lactis*, *L. waltii*, *S. castellii* (now *Nauvomozyma castellii*) and *P. anomala* (now *Wickerhamomyces anomalus*), which have only one [49, 74]. The presence of two genes in *Saccharomyces* (*sensu stricto*) species and only one in closely and distantly related species (noted above) may be the result of WGD during the evolution of ascomycete yeasts. The genus *Kazachstania*, along with several genera in the family *Saccharomycetaceae* (*Saccharomyces*, *Nakaseomyces* and *Tetrapisispora*) went through a WGD event (known as the post-WGD clade), which resulted in differential gene loss and gene duplications (the latter being referred to as ohnologues) [72, 75, 76]. Though *ATF1* and *ATF2* have similar functions, it is expected that only one orthologue is in pre-WGD species (*L. waltii* and *K. lactis*) as van Laere *et al*. [49] had suggested that *ATF2* in *S. cerevisiae* had retained its initial function of AATase pre-WGD, while *ATF1* had developed a new function, probably in anaerobic lipid metabolism. Moreover, the existence of one AATase gene in some post-WGD species could also be explained by reciprocal gene loss after speciation [49].

In the ADH family, seven genes have been identified and characterized in *S. cerevisiae* [77]. Almost every species has at least two *ADH* genes, although numbers vary and are diversified across species. In this study, three (or possibly four) putative genes coding for ADHs were found in *K. aerobia* and *K. servazzii* (*ADH1*, *ADH3, ADH6*/*ADH7*), with the *S. cerevisiae ADH5* sequence being the same as *ADH1* in both *Kazachstania* spp., suggesting gene duplication in the latter species. The repeated *ADH6*/*ADH7* homologues in *Kazachstania* spp. (four and three copies in *K. aerobia* and *K. servazzii*, respectively), which are a possible explanation for the enhanced formation of phenylethyl acetate by *Kazachstania* spp., as four paralogous *ADH6* genes were also found in *H. vineae* [24]. Crabtree-negative yeasts such as *K. lactis*, which is a poor fermentative species [78], have four *ADH* genes: *ADH1* and *ADH2* (which has a similar function to *ADH1* in *S. cerevisiae*), and *ADH3* and *ADH4* (mitochondrially encoded ADH), which possess reciprocal regulation properties. Recently, ethanol metabolism has been investigated in *K. phaffii*, with four ADH genes being identified (*ADH2*, *ADH6*, *ADH7*, *ADH900*) [79]. *ADH900* is the main gene responsible for ethanol production in *K. phaffii*, as *ADH2* plays a minor role in the absence of *ADH900* [79]. In contrast, the duplication of ADH encoding genes and WGD was suggested to be the origin of the Crabtree effect in *Saccharomycetaceae*, which had occurred after the split of WGD yeasts from the *Kluyveromyces* lineage [80]. Species belonging to the post-WGD lineage have a more pronounced Crabtree effect, with increased carbon metabolism under both anaerobic and aerobic conditions [80].

Regarding the biosynthesis of higher alcohols, the branched chain amino acid transaminases (BCAATases) catalyse the transfer of amino groups to α-keto acids, the precursors of higher (fusel) alcohols, which influence the aroma and flavour of yeast-derived fermentation products [45, 80]. In *S. cerevisiae*, BCAATases are encoded by two paralogous genes, *BAT1* and *BAT2*, that arose through a WGD event, as each perform different functions since *Bat1* is mitochondrially located while *Bat2* is cytosolic [81]. Both *Bat1* and *Bat2* orthologes were identified in *K. aerobia* and *K. servazzii*, as they both have high sequence similarity with the orthologues in closely related species (Tables S11 and S12). As *Kazachstania* spp. are high producers of acetate esters, in particular phenylethyl acetate [32, 33], the *in silico* analysis for the set of *ARO* genes (*ARO3, 4, 7* and *10*) involved in the Ehrlich pathway and the biosynthesis of 2-phenylethanol showed highly conserved sequences between *Kazachstania* spp. and *S. cerevisiae* (Figs S6–S9). The putative orthologues for *FAS2* and *TOR1* involved in the production of phenylethyl acetate (esterified from phenylethyl alcohol) in *S. cerevisiae* were also identified in *Kazachstania* spp., with high sequence similarity (Tables S13 and S14). Though AATases are primarily responsible for the production of acetate esters, the high production of phenylethyl acetate in *Kazachstania* spp. could be explained by the presence of the *TOR1* and *FAS2* genes.

In conclusion, these data contribute to and provide a good starting point to better understand the *Kazachstania* spp. genomes and their potential usefulness in winemaking and other applications (transcriptomic and metabolomic studies). While the exact function of these putative orthologous genes is unknown, further comparative functional genomics studies are required to characterize these genes and their genetic context.

## Supplementary Data

Supplementary material 1

Supplementary material 2
