# Supplementary material for: Genomic analysis of Kazachstania aerobia and Kazachstania servazzii reveals duplication of genes related to acetate ester production
Source: Microb Genom. 2023 Jun 5;9(6):mgen001029. doi: 10.1099/mgen.0.001029 (PMC10327501; doi:10.1099/mgen.0.001029)
Supplement: Supplementary material 1 [file mgen-9-1029-s001.pdf]

Supplementary Table 2. Protein sequence similarity and identity of *K. aerobia* Atf and *K. servazzii* Atf and orthologous proteins found in closely related species of the *Saccharomycetaceae* family.

	K. aerobia Atf		*K. servazzii* Atf	
	% similarity	% identity	% similarity	% identity
S. cerevisiae Atf2 YGR177c	60	39.96	60	38.78
K. africana hypothetical protein KAFR_0D01730	68	47.40	71	50.60
K. naganishii hypothetical protein KNAG_0H02650	67	47.57	66	47.09
N. castellii (CBS 4309) hypothetical protein NCAS_0A06920	61	42.80	61	43.03
N. dairenensis (CBS 421) hypothetical protein NDA_0D01980	59	39.52	60	39.51

Supplementary Table 3. Protein sequence similarity and identity of *K. aerobia* Eht1/Eeb1 and *K. servazzii* Eht1/Eeb1 and orthologous proteins found in closely related species of the *Saccharomycetaceae* family.

	K. aerobia Eht1/Eeb1		*K. servazzii* Eht1/Eeb1	
	% similarity	% identity	% similarity	% identity
S. cerevisiae Eht1 YBR177c	77	60.73	78	59.66
S. cerevisiae Eeb1 YPL095c	68	50.78	69	54.78
K. africana hypothetical protein KAFR_0I000680	76	61.74	77	60.43
K. naganishii hypothetical protein KNAG_0A03490	78	65.30	78	64.84
N. castellii (CBS 4309) hypothetical protein NCAS_0A03490	76	61.59	76	60.65
N. dairenensis (CBS 421) hypothetical protein NDA_0D01980	76	60.56	76	60.34

Supplementary Table 4. Protein sequence similarity and identity of *K. aerobia* Iah1 and *K. servazzii* Iah1 and orthologous proteins found in closely related species of the *Saccharomycetaceae* family.

	K. aerobia Iah1		*K. servazzii* Iah1	
	% similarity	% identity	% similarity	% identity
S. cerevisiae Iah1 YOR126C	70	50	71	46.67
K. africana hypothetical protein KAFR_0D05100	71	51.29	71	47.41
K. naganishii hypothetical protein KNAG_0B04160	73	52.56	72	50
N. castellii (CBS 4309) hypothetical protein NCAS_0H02130	73	46.81	69	44.68
N. dairenensis (CBS 421) hypothetical protein NDAI_0C01520	70	47.60	68	45.41

Supplementary Table 5. Protein sequence similarity and identity of *K. aerobia* Adh1 and *K. servazzii* Adh1 and orthologous proteins found in closely related species of the *Saccharomycetaceae* family.

	K. aerobia Adh1		*K. servazzii* Adh1	
	% similarity	% identity	% similarity	% identity
S. cerevisiae Adh1 YOL068C	91	84.64	90	84.64
K. africana hypothetical protein KAFR_0G01420	94	88.73	94	89.31
K. naganishii hypothetical protein KNAG_0K02000	94	87.50	93	87.79
N. castellii (CBS 4309) hypothetical protein NCAS_0I02350	93	86.05	93	86.63
N. dairenensis (CBS 421) hypothetical protein NDAI_0A06930	92	86.30	92	86.59

Supplementary Table 6. Protein sequence similarity and identity of *K. aerobia* Adh3 and *K. servazzii* Adh3 and orthologous proteins found in closely related species of the *Saccharomycetaceae* family.

	K. aerobia Adh3		*K. servazzii* Adh3	
	% similarity	% identity	% similarity	% identity
S. cerevisiae Adh3 YMR083W	89	81.94	88	82.50
K. africana hypothetical protein KAFR_0A00790	89	79.72	89	80
K. naganishii hypothetical protein KNAG_0E02590	92	86.97	92	88.03
N. castellii (CBS 4309) hypothetical protein NCAS_0A07780	88	81.38	88	82.18
N. dairenensis (CBS 421) hypothetical protein NDAI_0H02160	90	83.20	91	83.20

Supplementary Table 7. Protein sequence similarity and identity of *K. aerobia* Aro3 and *K. servazzii* Aro3 and orthologous proteins found in closely related species of the *Saccharomycetaceae* family.

	K. aerobia Aro3		*K. servazzii* Aro3	
	% similarity	% identity	% similarity	% identity
S. cerevisiae Aro3 YDR035W	92	84.41	93	84.41
K. africana hypothetical protein KAFR_0A01020	91	84.45	91	84.45
K. naganishii hypothetical protein KNAG_0H01240	91	84.14	91	83.33
N. castellii (CBS 4309) hypothetical protein NCAS_0A10560	93	87.63	93	87.10
N. dairenensis (CBS 421) hypothetical protein NDAI_0H05730	93	86.56	94	87.10

Supplementary Table 8. Protein sequence similarity and identity of *K. aerobia* Aro4 and *K. servazzii* Aro4 and orthologous proteins found in closely related species of the *Saccharomycetaceae* family.

	K. aerobia Aro4		*K. servazzii* Aro4	
	% similarity	% identity	% similarity	% identity
S. cerevisiae Aro4 YBR249C	91	85.41	91	83.42
K. africana hypothetical protein KAFR_0AG03630	92	86.03	92	84.43
K. naganishii hypothetical protein KNAG_0M00510	90	83.83	89	82.11
N. castellii (CBS 4309) hypothetical protein NCAS_0H01150	93	85.48	93	83.61
N. dairenensis (CBS 421) hypothetical protein NDAI_0F02150	92	86.68	92	84.93

Supplementary Table 9. Protein sequence similarity and identity of *K. aerobia* Aro7 and *K. servazzii* Aro7 and orthologous proteins found in closely related species of the *Saccharomycetaceae* family.

	K. aerobia Aro7		*K. servazzii* Aro7	
	% similarity	% identity	% similarity	% identity
S. cerevisiae Aro7 YPR060C	87	76.26	87	76.26
K. africana hypothetical protein KAFR_0H00990	88	76.06	89	75.68
K. naganishii hypothetical protein KNAG_0H00740	87	71.60	87	70.82
N. castellii (CBS 4309) hypothetical protein NCAS_0A11070	88	76.17	88	75.78
N. dairenensis (CBS 421) hypothetical protein NDAI_0A05230	88	75.42	88	75.85

Supplementary Table 10. Protein sequence similarity and identity of *K. aerobia* Aro10 and *K. servazzii* Aro10 and orthologous proteins found in closely related species of the *Saccharomycetaceae* family.

	K. aerobia Aro10		*K. servazzii* Aro10	
	% similarity	% identity	% similarity	% identity
S. cerevisiae Aro10 YDR380W	75	58.71	76	57.49
K. africana hypothetical protein KAFR_0E03730	77	61.75	77	60.65
K. naganishii hypothetical protein KNAG_0C04790	79	62.34	79	62.46
N. castellii (CBS 4309) hypothetical protein NCAS_0A11830	76	60	76	60.16
N. dairenensis (CBS 421) hypothetical protein NDAI_0A04450	78	59.96	78	59.75

Supplementary Table 11. Protein sequence similarity and identity of *K. aerobia* Bat1 and *K. servazzii* Bat1 and orthologous proteins found in closely related species of the *Saccharomycetaceae* family.

	K. aerobia Bat1		*K. servazzii* Bat1	
	% similarity	% identity	% similarity	% identity
S. cerevisiae Bat1 YHR208W	84	76.34	86	79.26
K. africana hypothetical protein KAFR_0B07030	87	79.59	88	80.53
K. naganishii hypothetical protein KNAG_0M00140	88	79.79	88	79.69
N. castellii (CBS 4309) hypothetical protein NCAS_0J02240	88	78.44	89	78.34
N. dairenensis (CBS 421) hypothetical protein NDAI_0D03470	90	81.40	89	80.11

Supplementary Table 12. Protein sequence similarity and identity of *K. aerobia* Bat2 and *K. servazzii* Bat2 and orthologous proteins found in closely related species of the *Saccharomycetaceae* family.

	K. aerobia Bat2		*K. servazzii* Bat2	
	% similarity	% identity	% similarity	% identity
S. cerevisiae Bat2 YJR148W	86	76.08	85	75.40
K. africana hypothetical protein KAFR_0E04480	89	79.41	89	79.62
K. naganishii hypothetical protein KNAG_0M00140	86	77.96	85	77.27
N. castellii (CBS 4309) hypothetical protein NCAS_0J02240	89	81.02	89	79.89
N. dairenensis (CBS 421) hypothetical protein NDAI_0D03470	89	78.02	89	77.87

Supplementary Table 13. Protein sequence similarity and identity of *K. aerobia* Fas2 and *K. servazzii* Fas2 and orthologous proteins found in closely related species of the *Saccharomycetaceae* family.

	K. aerobia Fas2		*K. servazzii* Fas2	
	% similarity	% identity	% similarity	% identity
S. cerevisiae Fas2 YPL231W	89	81.16	89	80.96
K. africana hypothetical protein KAFR_0F01200	90	81.54	89	81.39
K. naganishii hypothetical protein KNAG_0D00890	88	81.46	88	81.25
N. castellii (CBS 4309) hypothetical protein NCAS_0G01260	90	81.67	90	81.47
N. dairenensis (CBS 421) hypothetical protein NDAI_0F01390	89	81.66	89	81.34

Supplementary Table 14. Protein sequence similarity and identity of *K. aerobia* Tor1 and *K. servazzii* Tor1 and orthologous proteins found in closely related species of the *Saccharomycetaceae* family.

	K. aerobia Tor1		*K. servazzii* Tor1	
	% similarity	% identity	% similarity	% identity
S. cerevisiae Tor1 YJR066W	77	61.19	77	61.41
K. africana hypothetical protein KAFR_0A08100	80	63.99	80	64.39
K. naganishii hypothetical protein KNAG_0B00160	82	69.06	82	68.48
N. castellii (CBS 4309) hypothetical protein NCAS_0G03440	96	60.71	77	60.62
N. dairenensis (CBS 421)	0	0	0	0

Supplementary Table 15. Protein sequence similarity and identity of *K. aerobia* Adh6/Adh7 and *K. servazzii* Adh6/Adh7 and orthologous proteins found in closely related species of the *Saccharomycetaceae* family.

	K. aerobia contig17.g1558		*K. aerobia* contig19.g1873		*K. aerobia* contig_21.g2255		*K. aerobia* contig_3.g2902		*K. servazzii* contig_10.g24		*K. servazzii* contig_24.g2365		*K. servazzii* contig_ 25.g2372	
	% similarity	% identity	% similarity	% identity	% similarity	% identity	% similarity	% identity	% similarity	% identity	% similarity	% identity	% similarity	% identity
S. cerevisiae ADH6 YMR318C	79	66	82	70.83	85	73.82	84	73.74	80	66.11	85	74.58	86	74.30
S. cerevisiae ADH7 YCR105W	N/A	N/A	N/A	N/A	N/A	N/A	N/A	N/A	N/A	N/A	N/A	N/A	N/A	N/A
S. cerevisiae ADH6 CAY80561.2	78	63	84	73	85	62.85	82	70	79	65.83	83	71.39	83	70.56
S. cerevisiae ADH7 CAY82157.1	N/A	N/A	N/A	N/A	N/A	N/A	N/A	N/A	N/A	N/A	N/A	N/A	N/A	N/A
K. africana hypothetical protein KAFR_0C01550	88	76	83	70.39	84	71.51	83	70.67	89	77.09	83	71.51	84	70.67
K. naganishii hypothetical protein KNAG_0D00110	82	69.06	85	73.46	85	73.89	84	72.78	79	62.75	83	73.89	85	72.50
N. castellii (CBS 4309) hypothetical protein NCAS_0C03020	79	66	85	73.89	85	74.42	85	73.89	80	66.39	85	74.44	86	76.11
N. dairenensis NDAI_0A08470	80	67	87	76.06	88	76.62	87	76.62	82	69.92	87	76.90	88	78.59

N/A = not applicable as no significant similarity and/or identity were found in BLASTp search.

Supplementary Table 16. Protein sequence similarity and identity of *K. aerobia* Ald6 and *K. servazzii* Ald6 and orthologous proteins found in closely related species of the *Saccharomycetaceae* family.

	K. aerobia scaffold_15.g5207		*K. aerobia* scaffold_15.g5208		*K. servazzii* scaffold_20.g5254		*K. servazzii* scaffold_20.g5255	
	% similarity	% identity	% similarity	% identity	% similarity	% identity	% similarity	% identity
S. cerevisiae ALD6 YPL061W	88	77.17	87	73.54	88	75.96	87	75.15
S. cerevisiae ALD6 CAY86900.1	N/A	N/A	N/A	N/A	N/A	N/A	N/A	N/A
K. africana hypothetical protein KAFR_0A01210	88	78.79	87	75.86	88	78.70	87	75.66
K. naganishii hypothetical protein KNAG_0A02070	88	76.06	86	74.54	89	75.86	86	74.54
N. castellii (CBS 4309) hypothetical protein NCAS_0C02220	89	78.51	87	76.66	89	77.87	87	76.46
N. dairenensis hypothetical protein NDAI_0E02900	87	77.71	87	76.31	87	77.71	86	74.90

N/A = not applicable as no significant similarity and/or identity were found in BLASTp search.

Supplementary Table 17. Protein sequence similarity and identity of *K. aerobia* Bdh1 and *K. servazzii* Bdh1 and orthologous proteins found in closely related species of the *Saccharomycetaceae* family.

	K. aerobia contig_13.g329		*K. aerobia* contig_25.g2491		*K. aerobia* contig_30.g3329		*K. servazzii* contig_6.g4029	
	% similarity	% identity	% similarity	% identity	% similarity	% identity	% similarity	% identity
S. cerevisiae BDH1 YAL060W	71	57.74	74	59.58	72	57.74	74	59.58
S. cerevisiae BDH1 CAY77584.1	N/A	N/A	N/A	N/A	N/A	N/A	N/A	N/A
K. africana hypothetical protein KAFR_0I00170	72	56.69	72	59.58	72	56.43	73	59.84
K. naganishii hypothetical protein KNAG_0I00120	79	65.62	82	68.50	79	65.35	81	68.50
N. castellii (CBS 4309) hypothetical protein NCAS_0A07660	73	59.48	74	62.44	73	59.74	74	62.08
N. dairenensis NDAI_0H02050	74	58.96	75	63.12	75	58.96	75	62.50

N/A = not applicable as no significant similarity and/or identity were found in BLASTp search.

Supplementary Figure 1.

```
ATF1        MNEIDE------KNQAPVQQECLKEMIQNGHARRMGSVEDLYVALNRQNLYRNFCTYGEL    54
ATF2        -----------MEDIEGYEPHITQELIDRGHARRMGHLENYFAVLSRQKMYSNFTVYAEL    49
KA_ATF      MSVKDLEQENVIEHLLNESDGIDESMLERGHARRMGHLENYFALLQRQDLYGNFSCYCEY    60
KS_ATF      MSVKVHKQEEVVERLLKETDGIDDNMLERGHARRMGHLENYFALLQRQDLYGNFSCYCEY    60
                        :          ..:::.******* :*: :. *.**.:* **  * *

ATF1        SDYCTRDQLTLALREICLKNPTLLHIVLPTRWPNHENYYRSSEYYSRPHPVHDYISVLQE    114
ATF2        NKGVNKRQLMLVLKVLLQKYSTLAHTIIPKHYPHHEAYYSSEEYLSKPFPQHDFIKVISH    109
KA_ATF      DSSISVDRLAPVLREIFFKHPILVHTIIPKNYPNHESFYLDKEYLEQPYPEHDFIKVIPK    120
KS_ATF      DSSIDVNKLAPILREIFFKHPILVHTIIPKNYPNHESFYLDKEYLEQPYPEHDFIKVIPK    120
            ..     :*   *: :  *   * * ::*..:*:** :* ..** .:*.* **:*.*: .

ATF1        LKLSGVVLNEQPEYSAVMKQILEEFKNSKGSYTAKIFKLTTTLTIPYFGPTGPSWRLICL    174
ATF2        LEFDDLIMNNQPEYREVMEKISEQFKKDDFKVTNRLIELISPVIIPLGNPKRPNWRLICL    169
KA_ATF      LHLDDIIINNQEEYKDIISSIVEQFQNDKFEITEQLTEKVSKIRIPVCHPTKPNWRLLLL    180
KS_ATF      LHLNDIVINNQEEYKDIVSSIMDQFQKDKFEITEQLTEKVSQIRIPVCHSTKPNWRLLLL    180
            *.:..:::*:* **  ::..* ::*::.. . * :: :  : : **   .  *.***: *

ATF1        PEEH---TEKWKKFIFVSNHCMSDGRSSIHFFHDLRDELNNIKTP-------PKKLDYIF    224
ATF2        PGKDTDGFETWKNFVYVTNHCGSDGVSGSNFFKDLALLFCKIEEKGFDYDEEFIEDQVII    229
KA_ATF      PEGD--DQTKLKHIVYISNHCSSDATSGINLFKDIAEGLSQEDVA-----SSDANTSLIY    233
KS_ATF      PEND--DYSKLMHIVYISNHCSSDATSGINLFKDIAEGLSFEDIT-----PSDGNDPLIY    233
            *  .     .  ::::::*** **. *. ::*:*:   :   .           :   *

ATF1        KYEEDYQLLRKLPEPIEKVIDFRPPYLFIPKSLLSGFIYNHLRFSSKGVCMR---MDDVE    281
ATF2        DYDRDYTEISKLPKPITDRIDYKPALTSLPKFFLTTFIYEHCNFKTSSESTLTARYSPSS    289
KA_ATF      DYEIDYEKFVRIPIPITERIDYRPGMVAMGKFIGTTMIMNYLTFKFKDSQTAKI--KE--    289
KS_ATF      DYELDHEKFSRIPVPITERIDYRPGMVAMGKFIGTTMVMNYLTFKFKDSQTAKI--KE--    289
            .*: *:  : ::* ** . **::*    : * : : :: ::  *. ..        .

ATF1        KTDDVVTEIINISPTEFQAIKANIKSNIQGKCTITPFLHVCWFVSLHKWGKFFKPLNFEW    341
ATF2        NANASYNYLLHFSTKQVEQIRAQIKKNVHDGCTLTPFIQACFLVALYRLDKLFTKSLLEY    349
KA_ATF      DLRENFHYNLNISWEELTNLKLV---LLQHQSSITGFLQACLFIVLAEQGIFKEKKWNEM    346
KS_ATF      DLRQNYHYNLNITWDELTSLKLI---LLKHESSITGFLQACLFITLTEQGIFKDKKWNEM    346
            .        ::::  :.  ::      ::  .::* *::.* :: * . . :      *

ATF1        LTDIFIPADCRSQLPDDDEMRQMYRYGANVGFIDFTPWISEFDMND-NKENFWPLIEHYH    400
ATF2        GFDVAIPSNARRFLPNDEELRDSYKYGSNVGGSHYAYLISSFDIPEGDNDKFWSLVEYYY    409
KA_ATF      GFDMSIPNDNRKNLPAE-LVEEQYKYGSNVGGSHYSFLLSSFK-----RDQLWELSKYYT    400
KS_ATF      GFDMSIPNDNRKNLPSE-LVEQQYKYGSNVGGSHYSFLLSSFK-----RDQLWELSKYYT    400
              *: ** : *  ** :  :.: *:**:***  .::  :*.*.     .:::* * ::*

ATF1        EVISEALRNKKHLHGLGFNIQGFVQKYVNIDKVMCDRAIGKRRGGTLLSNVGLFNQLEEP    460
ATF2        DRFLESYDNGDHLIGLGVLQLDFIVENKNIDSLLANSYLHQQRGGAIISNTGLVSQDT--    467
KA_ATF      SV----IRNADYNVGLGTLMLDMVYKKQNVDKIISESYLGNQRGGIILSNIGLHEHKG--    454
KS_ATF      NV----IKNADYNVGLGTLMLDMVYKKQNVDKIISESYLGNKRGGIILSNIGLHQHKG--    454
            .       * .:  ***    .:: :  *:*.::.:  : ::*** ::** ** .:

ATF1        DAKYSICDLAFGQFQGSWHQAFSLGVCSTNVKGMNIVVASTKNVVGSQESLEELCSIYKA    520
ATF2        TKPYYVRDLIFSQSAGALRFAFGLNVCSTNVNGMNMDMSVVQGTLRDRGEWESFCKLFYQ    527
KA_ATF      --GIGIKDLKFVQDVGALNFALVVNACSTKTKGMNICISGVEGTIGDREQFTSTGDALKS    512
KS_ATF      --GIGIQDLKFVQDVGALNFALVVNACSTKMKGMNICMSGIEGTIGDREQFNSTGDALKA    512
                 : ** * *  *: . *: :..***: :***: ::  :..: .: .  .  .

ATF1        LLLGP*---     525
ATF2        TIGEFASL*     535
KA_ATF      LIQEYCK--     519
KS_ATF      LIHEYCK--     519
             :
```

Supplementary Figure 2.

```
KA_EHT1/EEB1      MPSEQELMKDINTWPLLNPFHWGYNGTVHHSIGANGTVELHLNEDATVKDELDTKGKITL        60
KS_EHT1/EEB1      MPSEQELMKDINTWPLLNPFHWGYNGSVHHSIGANGTVELQLNEDVTNKDELDTKGKIAL        60
EHT1              -------MSEVSKWPAINPFHWGYNGTVSHIVGENGSIKLHLKD---------NKEQVDF        44
EEB1              -------MFRSGYYPTVTPSHWGYNGTVKHVLGEKGTKSLAFRD---------SKRQIPL        44
                         *   . :* :.* ******:* * :* :*: .* :.:         .* :: :

KA_EHT1/EEB1      DQFVNEHVPGLQDGAKFQLDKKLFTGILQTMYLGAADFSQKFPVFYGREIFEFSDKGACT        120
KS_EHT1/EEB1      DEFVNEHVPGLRDGAKFQLDKKLFTGILQTMYLGAADFSQKFPVFYGREIVEFSDKGACT        120
EHT1              DEFANKYVPTLKNGAQFKLSPYLFTGILQTLYLGAADFSKKFPVFYGREIVKFSDGGVCT        104
EEB1              HEFVTKHVPTLKDGANFRLNSLLFTGYLQTLYLSAGDFSKKFQVFYGREIIKFSDGGVCT        104
                  .:*..::** *::**:*:*.  **** ***:**.*.***:** *******.:*** *.**

KA_EHT1/EEB1      ADWVMNGSWRDRYHFDYATGKFDKQLFNEDEKKTHPENWPRLQPRTRYLDEEELKTVHDE        180
KS_EHT1/EEB1      ADWVMNGSWRERYQYEAATGKFDKQLFNEDEKKTHPENWPRLQPRTRYLDQEELKTVHDE        180
EHT1              ADWLID-SWKKDYEFDQSTTSFDKKKFDKDEKATHPEGWPRLQPRTRYLKDNELEELR--        161
EEB1              ADWVMP-EWEQTYSLNAEKASFNEKQFSNDEKATHPKGWPRLHPRTRYLSSEELEKCHS-        162
                  ***::  .*.. *  :  . .*::: *.:*** ***:.****:******..:**:  :

KA_EHT1/EEB1      KNNERPLVVILHGLAGGSHEPIIRSLTDHLSHASNGKFQVVVLNSRGCARSKITTPNLFT        240
KS_EHT1/EEB1      KNNERPLVVILHGLAGGSHEPIIRSLTDHLSHASNGKFQVVVLNSRGCARSKITTPNLFT        240
EHT1              -EVDLPLVVILHGLAGGSHEPIIRSLAENLSR--SGRFQVVVLNTRGCARSKITTRNLFT        218
EEB1              KGYSYPLVVVLHGLAGGSHEPLIRALSEDLSKVGDGKFQVVVLNARGCSRSKVTTRRIFT        222
                     . ****:***********:**:*::.**:  .*:*******:***:***:** .:**

KA_EHT1/EEB1      AFHSMDIQEFLDRERAKSPNRKLYAIGCSFGATILANYLGFAGDKTPLNAAATFCNPWDM        300
KS_EHT1/EEB1      AFHSMDIKEFLDRERAKSPTRKLYAIGCSFGATILANYLGFAGDKTPLNAAATFCNPWDM        300
EHT1              AYHTMDIREFLQREKQRHPDRKLYAVGCSFGATMLANYLGEEGDKSPLSAAATLCNPWDL        278
EEB1              ALHTGDVREFLNHQKALFPQRKIYAVGTSFGAAMLTNYLGEEGDNCPLNAAVALSNPWDF        282
                  * *: *::***::::   * **:**:* ****::*:****  **: **.**.::.****:

KA_EHT1/EEB1      VLSSYKVGRDYWSQRIFSKTVTQFLVRMIKVNMKELEVPEGTKPDHVPTPENPSFTGFTQ        360
KS_EHT1/EEB1      VLSSYKVGRDYWSQRIFSKTVTQFLVRMIKVNMNELEVPEGTLPDHVPSPEHPSFTGFTQ        360
EHT1              LLSAIRMSQDWWSRTLFSKNIAQFLTRTVQVNMGELGVPNGSLPDHPPTVKNPSFYMFTP        338
EEB1              VHTWDKLAHDWWSNHIFSRTLTQFLTRTVKVNMNELQVPENFEVSHKPTVEKPVFYTYTR        342
                  : :  ::.:*:**. :**:.::***.* ::*** ** **:.   .* *: ::* *  :*

KA_EHT1/EEB1      SNLQKAFKMKFMSEFDSTFTAPALGFKDALDYYKHASSVNRLTNIAIPLLSLNSMDDPVV        420
KS_EHT1/EEB1      SNLQKAFKMRFMSEFDSMFTAPALGFKDALDYYKHASSVNRLTNIAIPLLSLNSMDDPVV        420
EHT1              ENLIKAKSFKSTREFDEVYTAPALGFPNAMEYYKAASSINRVDTIRVPTLVINSRDDPVV        398
EEB1              ENLEKAEKFTDILEFDNLFTAPSMGLPDGLTYYRKASSINRLPNIKIPTLIINATDDPVT        402
                  .** ** .:    ***. :***::*: :.: **: ***:**: .* :* * :*: ****.

KA_EHT1/EEB1      GSDHIPKSYLDQNPNVLMCETDLGGHLAYLQSDGDSWATRQICTFFDKFDELVK-   474
KS_EHT1/EEB1      GSDHIPKTYLDQNPNVLMCETDLGGHLAYLQSDGDSWATRQICNFFDKFDELIK-   474
EHT1              GPD-QPYSIVEKNPRILYCRTDLGGHLAYLDKDNNSWATKAIAEFFTKFDELVV*   451
EEB1              GENVIPYKQARENPCVLLCETDLGGHLAYLDNESNSWLTKQAAEFLGSFDELVL*   456
                  * :  * .   :** :* *.**********:.:.:** *:  . *: .****:
```

Supplementary Figure 3.

```
IAH1        MDYEKFLLFGDSITEFAFNTRPIEDGKDQYALGAALVNEYTRKMDILQRGFKGYTSRWAL   60
KA_IAH1     MNYPKFLLFGDSITEFSFDP-------EHFTVGSALTNVYTRKLDVVQRGYSGYTSRWAI   53
KS_IAH1     MNYPKFLLFGDSITEFAYDP-------EHFTVGSALSNVYTRKLDVVQRGYSGFTSRWAI   53
            *:* ************:::        :::::*:** * ****:*::***:.*:*****:

IAH1        KILPEILKH-ESNIVMATIFLGANDACSAGPQSVPLPEFIDNIRQMVSLMKSYHIRPIII   119
KA_IAH1     PILEKIIASDGEGIVMGTIFFGSNDSVVAGPQRVPLPEFIENTKRLIQMMKDASIKPIVA   113
KS_IAH1     PVLENIIASDGEGIVMGTIFFGSNDSVAAGPQRVPLPEFLDNTKRLIHMMKDANIKPIVV   113
             :* :*:    ..***.***:*:**:  **** ******::* :::: :**.  *:**:

IAH1        GPGLVDREKWEKEKSEEIALGYFRTNENFAIYSDALAKLANEEKVPFVALNKAFQQE---   176
KA_IAH1     GPALINRELWDVLKKEDIDQGWIRSNEAFQEYSDALIKLTHEENVPYINLRQSFLDHAKA   173
KS_IAH1     GPGLINRDLWDVLKKDDIDKGWIRSNETFREYNDALMQLTKDENVPYINLRQSFLDTAEA   173
            **.*::*: *:  *.::*  *::*:** *  *.*** :*:::*:**:: *.::* :

IAH1        GGDAWQQLLTDGLHFSGKGYKIFHDELLKVIETFYPQYHPKNMQYKLKDWRDVLDDGSNI   236
KA_IAH1     KNEDWKTYTIDGLHFSGAGYRIYFDQLMKTIDRYYPEYSPDNLKTFLPNWRDVQEDGSNI   233
KS_IAH1     KNEDWKTYTVDGLHFSGAGYRVYFDQLMKTIDHYYPEYSPVNLKTILPNWRDIQEDGSNI   233
             .: *:    ******* **:::.*:*:*.*: :**:* * *::  * :***: :*****

IAH1        MS* 238
KA_IAH1     F-- 234
KS_IAH1     F-- 234
            :
```

Supplementary Figure 4a.

```
ADH1            MSIPETQKGVIFYESHGKLEYKDIPVPKPKANELLINVKYSGVCHTDLHAWHGDWPLPVK    60
KA_ADH1         MSIPTTQKGVVFETNGGKLEYKEIPVPKPRANELLINVKYSGVCHTDLHAWKGDWPLPVK    60
KS_ADH1         MSIPTTQKGVIFETNGGKLEYKEIPVPTPKANELLINVKYSGVCHTDLHAWKGDWPLPVK    60
                **** *****:*  . ******:****.*:*********************:********

ADH1            LPLVGGHEGAGVVVGMGENVKGWKIGDYAGIKWLNGSCMACEYCELGNESNCPHADLSGY    120
KA_ADH1         LPLVGGHEGAGVVVAMGENVKGWKIGDFAGIKWLNGSCMNCEYCELSNESNCPDADLSGY    120
KS_ADH1         LPLVGGHEGAGVVVAMGENVKGWKIGDFAGIKWLNGSCMNCEYCELSNESNCPDADLSGY    120
                **************.************:*********** ******.******.******

ADH1            THDGSFQQYATADAVQAAHIPQGTDLAQVAPILCAGITVYKALKSANLMAGHWVAISGAA    180
KA_ADH1         THDGSFQQYATADAVQAAKIPAGTDLAQVAPILCAGVTVYKALKSANLRAGEWVAISGAC    180
KS_ADH1         THDGSFQQYATADAVQAAKIPAGTDLANVAPILCAGVTVYKALKSANLRAGEWVAISGAC    180
                ******************:** *****:********:*********** **.*******.

ADH1            GGLGSLAVQYAKAMGYRVLGIDGGEGKEELFRSIGGEVFIDFTKEKDIVGAVLKATDGGA    240
KA_ADH1         GGLGSLAIQYATAMGYRVLGIDGGDEKAKLFKELGGEHFVDFTKTKDIEGDIIKATNGGA    240
KS_ADH1         GGLGSLAIQYATAMGYRVLGIDGGDEKAKLFKELGGEHFVDFTKTKDIEGDIIKATNGGA    240
                *******:***.************: * :**:.:*** *:**** *** * ::***:***

ADH1            HGVINVSVSEAAIEASTRYVRANGTTVLVGMPAGAKCCSDVFNQVVKSISIVGSYVGNRA    300
KA_ADH1         HGVINVSVSEAAIEASTRYVRANGTVVLVGLPAGAVCKSEVFSHVVKSISIVGSYVGNRA    300
KS_ADH1         HGVINVSVSEAAIEASTRYVRANGTVVLVGLPAGAVCKSEVFSHVVKSISIVGSYVGNRA    300
                *************************.****:**** * *:**.:****************

ADH1            DTREALDFFARGLVKSPIKVVGLSTLPEIYEKMEKGQIVGRYVVDTSK*      348
KA_ADH1         DTREALDFFSRGLVKSPIIIAPLSDLPEIFDKMEKGQIVGRYVVNCDN-      348
KS_ADH1         DTREALDFFVRGLVKSPIIIAPLSDLPEIFDKMEKGQIVGRYVVNCDN-      348
                ********* ********:.  ** ****::*************: .: 
```

Supplementary Figure 4b.

```
ADH5            MPSQVIPEKQKAIVFYETDGKLEYKDVTVPEPKPNEILVHVKYSGVCHSDLHAWHGDWPF    60
ADH1            ---MSIPETQKGVIFYESHGKLEYKDIPVPKPKANELLINVKYSGVCHTDLHAWHGDWPL    57
KA_ADH1         ---MSIPTTQKGVVFETNGGKLEYKEIPVPKPRANELLINVKYSGVCHTDLHAWKGDWPL    57
KS_ADH1         ---MSIPTTQKGVIFETNGGKLEYKEIPVPTPKANELLINVKYSGVCHTDLHAWKGDWPL    57
                     ** .**.::*  . ******:: ** *: **:*::********:*****:****:

ADH5            QLKFPLIGGHEGAGVVVKLGSNVKGWKVGDFAGIKWLNGTCMSCEYCEVGNESQCPYLDG    120
ADH1            PVKLPLVGGHEGAGVVVGMGENVKGWKIGDYAGIKWLNGSCMACEYCELGNESNCPHADL    117
KA_ADH1         PVKLPLVGGHEGAGVVVAMGENVKGWKIGDFAGIKWLNGSCMNCEYCELSNESNCPDADL    117
KS_ADH1         PVKLPLVGGHEGAGVVVAMGENVKGWKIGDFAGIKWLNGSCMNCEYCELSNESNCPDADL    117
                 :*:**:********** :*.******:**:********:** *****:.***:**  *

ADH5            TGFTHDGTFQEYATADAVQAAHIPPNVNLAEVAPILCAGITVYKALKRANVIPGQWVTIS    180
ADH1            SGYTHDGSFQQYATADAVQAAHIPQGTDLAQVAPILCAGITVYKALKSANLMAGHWVAIS    177
KA_ADH1         SGYTHDGSFQQYATADAVQAAKIPAGTDLAQVAPILCAGVTVYKALKSANLRAGEWVAIS    177
KS_ADH1         SGYTHDGSFQQYATADAVQAAKIPAGTDLANVAPILCAGVTVYKALKSANLRAGEWVAIS    177
                :*:****:**:**********:** ..:**:********:******* **:  *.**:**

ADH5            GACGGLGSLAIQYALAMGYRVIGIDGGNAKRKLFEQLGGEIFIDFTEEKDIVGAIIKATN    240
ADH1            GAAGGLGSLAVQYAKAMGYRVLGIDGGEGKEELFRSIGGEVFIDFTKEKDIVGAVLKATD    237
KA_ADH1         GACGGLGSLAIQYATAMGYRVLGIDGGDEKAKLFKELGGEHFVDFTKTKDIEGDIIKATN    237
KS_ADH1         GACGGLGSLAIQYATAMGYRVLGIDGGDEKAKLFKELGGEHFVDFTKTKDIEGDIIKATN    237
                **.*******:*** ******:*****: * :**..:*** *:***: *** * ::***:

ADH5            GGSHGVINVSVSEAAIEASTRYCRPNGTVVLVGMPAHAYCNSDVFNQVVKSISIVGSCVG    300
ADH1            GGAHGVINVSVSEAAIEASTRYVRANGTTVLVGMPAGAKCCSDVFNQVVKSISIVGSYVG    297
KA_ADH1         GGAHGVINVSVSEAAIEASTRYVRANGTVVLVGLPAGAVCKSEVFSHVVKSISIVGSYVG    297
KS_ADH1         GGAHGVINVSVSEAAIEASTRYVRANGTVVLVGLPAGAVCKSEVFSHVVKSISIVGSYVG    297
                **:******************* * ***.****:** * * *:**.:********** **

ADH5            NRADTREALDFFARGLIKSPIHLAGLSDVPEIFAKMEKGEIVGRYVVETSK*   351
ADH1            NRADTREALDFFARGLVKSPIKVVGLSTLPEIYEKMEKGQIVGRYVVDTSK*   348
KA_ADH1         NRADTREALDFFSRGLVKSPIIIAPLSDLPEIFDKMEKGQIVGRYVVNCDN-   348
KS_ADH1         NRADTREALDFFVRGLVKSPIIIAPLSDLPEIFDKMEKGQIVGRYVVNCDN-   348
                ************ ***:**** :. ** :***: *****:*******: .:
```

Supplementary Figure 5.

```
ADH3                MLRTSTLFTRRVQPSLFSRNILRLQSTAAIPKTQKGVIFYENKGKLHYKDIPVPEPKPNE        60
KA_ADH3             MLKLITQH--QTVARAGFKQFSRLQSTFTIPSTQKGVVFYEHGGELHYKDIPVPKPKPNE        58
KS_ADH3             MLKLITQH--QTVARSGFKQFSRLQSTFTIPSTQKGVIFYEHGGQLHYKDIPVPKPKPNE        58
                    **:  * .  :.      ::: ***** :**.*****:***: *:*********:*****

ADH3                ILINVKYSGVCHTDLHAWHGDWPLPVKLPLVGGHEGAGVVVKLGSNVKGWKVGDLAGIKW        120
KA_ADH3             ILINVKYSGVCHTDLHAWKGDWPLPVKLPLVGGHEGAGVVVAKGENVKNFKIGDLAGIKW        118
KS_ADH3             ILINVKYSGVCHTDLHAWKGDWPLPVKLPLVGGHEGAGVVVAKGENVTNFKIGDLAGIKW        118
                    ******************:**********************  *.**..:*:********

ADH3                LNGSCMTCEFCESGHESNCPDADLSGYTHDGSFQQFATADAIQAAKIQQGTDLAEVAPIL        180
KA_ADH3             LNGSCMSCELCESGHESNCKQADLSGYTHDGSFQQYATADAVQAAKIQPGTNLAEVAPVL        178
KS_ADH3             LNGSCMSCELCESGHESNCEHADLSGYTHDGSFQQYATADAVQAAKIQPGTNLAEVAPVL        178
                    ******:**:********* .**************:*****:****** **:******:*

ADH3                CAGVTVYKALKEADLKAGDWVAISGAAGGLGSLAVQYATAMGYRVLGIDAGEEKEKLFKK        240
KA_ADH3             CAGITVYKAIKEANLRPGQWICISGAAGGLGSLAVQYAKCMGLRVIGIDGGPGKKELFES        238
KS_ADH3             CAGITVYKAIKEANLRPGQWICISGAAGGLGSLAVQYAKCMGLRVLGIDGGPGKKELFES        238
                    ***:*****:***:*: *:*:.****************..** **:***.*  *::**:.

ADH3                LGGEVFIDFTKT---KNMVSDIQEATKGGPHGVINVSVSEAAISLSTEYVRPCGTVVLVG        297
KA_ADH3             LGGETFIDFTKYKEPKDMVRAIQDATKGGPHGVVNVSVSEAAISLSTEYVRACGTVVLVG        298
KS_ADH3             LGGECFIDFTKHKDPKDMVRAIQDATKGGPHGVINVSVSEAAISLSTEYVRACGTVVLVG        298
                    **** ******    *:**  **:*********:***************** ********

ADH3                LPANAYVKSEVFSHVVKSINIKGSYVGNRADTREALDFFSRGLIKSPIKIVGLSELPKVY        357
KA_ADH3             LPAHSVVKSNVFSHVVKSINIKGSYVGNRADTREALDFFSRGLIKSPIKVVGLSELPKVY        358
KS_ADH3             LPAHSIVKSDVFSHVVKSINIKGSYVGNRADTREALDFFSRGLIKSPIKIVGLSELPKVY        358
                    ***:: ***:***************************************:**********

ADH3                DLMEKGKILGRYVVDTSK* 375
KA_ADH3             ELMEAGKILGRYVVDTSK- 376
KS_ADH3             ELMEAGKILGRYVVDTAK- 376
                    :*** ***********:*
```

Supplementary Figure 6.

```
ARO3            MFIKNDHAGDRKRLEDWRIKGYDPLTPPDLLQHEFPISAKGEENIIKARDSVCDILNGKD   60
KA_ARO3         MFIKNEHAGNRKRLEDWRIKGYDPLTPPDLLQHEYPISEQGEKNIVEAREGVCKVLNGED   60
KS_ARO3         MFIKNEHAGNRKRLEDWRIKGYDPLTPPDLLQHEYPISEQGEKHIVEAREGVCKVLNGED   60
                *****:***:************************:*** :**::*::**:.**.:***:*

ARO3            DRLVIVIGPCSLHDPKAAYDYADRLAKISEKLSKDLLIIMRAYLEKPRTTVGWKGLINDP   120
KA_ARO3         DRLVIVIGPCSIHDPQAAYEYCDRLQKISQKLSGDLLIIMRAYLEKPRTTVGWKGLINDP   120
KS_ARO3         DRLVIVIGPCSIHDPQAAYEYCDRLQKISQKLSGDLLIIMRAYLEKPRTTVGWKGLINDP   120
                ***********:***:***:*.*** ***:*** **************************

ARO3            DMNNSFQINKGLRISREMFIKLVEKLPIAGEMLDTISPQFLSDCFSLGAIGARTTESQLH   180
KA_ARO3         DIDNSFQINKGLRISREMFTKLVEKLPIAGEMLDTISPQFLSDCFSLGAIGARTTESQLH   180
KS_ARO3         DIDNSFQINKGLRISREMFTKLVEKLPIAGEMLDTISPQFLSDCFSLGAIGARTTESQLH   180
                *::**************** ****************************************

ARO3            RELASGLSFPIGFKNGTDGGLQVAIDAMRAAAHEHYFLSVTKPGVTAIVGTEGNKDTFLI   240
KA_ARO3         RELASGLSFPIGFKNGTDGGLQVAIDAMRAAAHEHYFLSVTKPGITAIVGTEGNADTFII   240
KS_ARO3         RELASGLSFPIGFKNGTDGGLQVAIDAMRAAAHEHYFLSVTKPGITAIVGTEGNADTFII   240
                ********************************************:********* ***:*

ARO3            LRGGKNGTNFDKESVQNTKKQLEKAGLTDD--SQKRIMIDCSHGNSNKDFKNQPKVAKCI   298
KA_ARO3         LRGGKNGTNFDAESVKSAKDQLAKANLLDAEGKKRRIMIDCSHGNSNKDYRNQPKVAQTI   300
KS_ARO3         LRGGKNGTNFDAESVKSAKEQLLKANLLDTEGKKRRIMIDCSHGNSDKDFRNQPKVAQTI   300
                *********** ***:.:*.** **.* *   .::***********:**::******: *

ARO3            YDQLTEGENSLCGVMIESNINEGRQDIPKEGGREGLKYGCSVTDACIGWESTEQVLELLA   358
KA_ARO3         YDQLVAGENSLCGVMIESNLVEGRQDVPPEGGRAGLKYGCSITDACIGWDSTEDVLELLA   360
KS_ARO3         YDQLVAGENSLCGVMIESNLVEGRQDVPPEGGRAGLKYGCSITDACIGWESTEDVLELLA   360
                ****. *************: *****:* **** *******:*******:***:******

ARO3            EGVRNRRKALKK*         370
KA_ARO3         EGVRKRRTILQK-         372
KS_ARO3         EGVRKRRSILQK-         372
                ****:**. *:*
```

Supplementary Figure 7.

```
ARO4           MSESPMFAANGMPKVNQGAEEDVRILGYDPLASPALLQVQIPATPTSLETAKRGRREAID   60
KA_ARO4        MSQSPLFNAN------DEASEDVRILGYDPLVSPALLQVQIPASQESIETAKRGRKESIE   54
KS_ARO4        MSQSPLFNAN------DESSEDVRILGYDPLVSPALLQVQVPASQNCIDTAKRGRKESID   54
               **:**:* **      : :.***********.********:**:  .::******:*:*:

ARO4           IITGKDDRVLVIVGPCSIHDLEAAQEYALRLKKLSDELKGDLSIIMRAYLEKPRTTVGWK   120
KA_ARO4        IITGKDDRVLVIVGPCSIHDLDAAQEYAIKLKALSDELQNDLLIVMRAYLEKPRTTVGWK   114
KS_ARO4        IITGKDDRILVVVGPCSIHDLDAAQEYAIKLKALSDELSKDLCIVMRAYLEKPRTTVGWK   114
               ********:**:*********:******::** *****. ** *:***************

ARO4           GLINDPDVNNTFNINKGLQSARQLFVNLTNIGLPIGSEMLDTISPQYLADLVSFGAIGAR   180
KA_ARO4        GLINDPDVNNTFNINKGLQAARQLFVNLTSLGLPIGSEMLDTISPQYLSDLLSFGAIGAR   174
KS_ARO4        GLINDPDVNNTFNINKGLQAARQLFVNLTSLGLPIGSEMLDTISPQYLSDLLSFGAIGAR   174
               *******************:*********.:*****************:**:********

ARO4           TTESQLHRELASGLSFPVGFKNGTDGTLNVAVDACQAAAHSHHFMGVTKHGVAAITTTKG   240
KA_ARO4        TTESQLHRELASGLSFPIGFKNGTDGTLNVAIDACQAASHSHHFMGVTKHGVAAITTTKG   234
KS_ARO4        TTESQLHRELASGLSFPIGFKNGTDGTLNVAIDACQAASHSHHFMGVTKHGVAAITTTKG   234
               *****************:*************:******:*********************

ARO4           NEHCFVILRGGKKGTNYDAKSVAEAKAQLPAGSNGLMIDYSHGNSNKDFRNQPKVNDVVC   300
KA_ARO4        NEHCFVILRGGKKGTNYDAKSVAEAKAALPKGANGLMIDYSHGNSEKDFRNQPKVNDVVC   294
KS_ARO4        NEHCFVVLRGGKKGTNYDAKSVAEAKSVLPKGSNGLMIDYSHGNSEKDFRNQPKVNDVVC   294
               ******:*******************: ** *:************:**************

ARO4           EQIANGENAITGVMIESNINEGNQGIPAEGKAGLKYGVSITDACIGWETTEDVLRKLAAA   360
KA_ARO4        EQIASGEMAITGVMIESNINEGNQPVVPGGKKALKYGVSITDGCISWETTDTVLRKLAAA   354
KS_ARO4        EQIANGEMSITGVMIESNINEGNQPVVPGGKKALKYGVSITDGCISWETTDTVLRKLAAA   354
               ****.** :*************** :   ** .*********.**.****: ********

ARO4           VRQRREVNKK* 370
KA_ARO4        VRARREVNKK- 364
KS_ARO4        VRARREINNNK 365
               ** ***:*::
```

Supplementary Figure 8.

```
ARO7          MDFTKPETVLNLQNIRDELVRMEDSIIFKFIERSHFATCPSVYEANHPGLEI-PNFKGSF   59
KA_ARO7       MDFTKPETVLNLQNIRDELIKMEDSIIFKFIERSHFPTCNGVYQIDNPNITLGSDFHGSF   60
KS_ARO7       MDFTKPETVLNLQNIRDELIKMEDSIIFKFIERSHFPTCNGVYQIDHPNITLGQDFNGSF   60
              *******************::*************** ** .**: ::*.: :  :*:***

ARO7          LDWALSNLEIAHSRIRRFESPDETPFFPDKIQKSFLPSINYPQILAPYAPEVNYNDKIKK   119
KA_ARO7       LDWALMQLEITHSQLRRFESPDQTPFFPNSLKKSILPSINYPKLLSNYSNEVNYNDKIKK   120
KS_ARO7       LDWALMQLEITHSQLRRFESPDQTPFFPNNLKKSILPSINYPKLLSNYSNEVNYNDKIKK   120
              ***** :***:**::*******:*****:.::**:*******::*: *: **********

ARO7          VYIEKIIPLISKRDGDDKNNFGSVATRDIECLQSLSRRIHFGKFVAEAKFQSDIPLYTKL   179
KA_ARO7       IYIEQMVPLISKIDKDDPNNYGSIASCDIECLQSLSRRIHFGKFVAEAKYQSNKELYNKL   180
KS_ARO7       IYIEQMVPLISKIDKDDPNNYGSIASCDIECLQSLSRRIHFGKFVAEAKYQSNKQLYNKL   180
              :***:::***** * ** **:**:*: **********************:**:  **.**

ARO7          IKSKDVEGIMKNITNSAVEEKILERLTKKAEVYGVDPTNESGERRITPEYLVKIYKEIVI   239
KA_ARO7       IQDKNVEGIMHEITNSAVEAKILERLTTKAEVYGVDPTNAEGERRITPEYLVKIYKEFVI   240
KS_ARO7       IQDKNIEGIMHEITNSAVEAKILERLTTKAEVYGVDPTNAEGERRITPEYLVKIYKEFVI   240
              *:.*::****::******* *******.*********** .****************:**

ARO7          PITKEVEVEYLLRRLEE*-  256
KA_ARO7       PITKEVEVEYLLRRLEDPN  259
KS_ARO7       PITKEVEVEYLLRRLEDPN  259
              ****************:
```

Supplementary Figure 9.

```
ARO10           ---------------MAPVTIEKFV---NQEERHLVSNRSATIPFGEYIFKRLLSIDTKS     42
KA_ARO10        MILSSEDKETYINNLPNSSILDTFGEKQAPVPSTAVATLSSQIPLGQYIFQRIITAGTKS     60
KS_ARO10        MILSSEDKETYIGNFTNSAILDTFGEKQAPAPSNTVTTLSSQIPLGQYLFQRIITAGTKS     60
                                    ::.*           *:. *: **:*:*:*:*::: .***

ARO10           VFGVPGDFNLSLLEYLYSPSVESAGLRWVGTCNELNAAYAADGYSRYSNKIGCLITTYGV     102
KA_ARO10        IFGVPGDFNLPLLEYLYEPTLLKQGLRWIGNCNELNSAYSADGFSRYSNKIGVIITTYGV     120
KS_ARO10        IFGVPGDFNLPLLEYLYEPGLLKQGLRWIGNCNELNSAYSADGFSRYSNKIGVVVTTYGV     120
                :********* ******.* : . ****:*.*****:**:***:******** ::*****

ARO10           GELSALNGIAGSFAENVKVLHIVGVAKSIDSRSSN-FSDRNLHHLVPQLHDSNFKGPNHK     161
KA_ARO10        GELSAMNGVAGAFAENVKLLHIVGVAKETDSRPGSLNKDKNIHHLIPNLKDSNFVGPNHK     180
KS_ARO10        GELSAMNGIAGAFAENVKLLHIVGVAKETDSRPDSTNANKNIHHLIPNLKNSNFVGPNHK     180
                *****:**:**:******:********. *** ..   ::*:***:*:*::*** *****

ARO10           VYHDMVKDRVACSVAYLEDIETACDQVDNVIRDIYKYSKPGYIFVPADFADMSVTCDNLV     221
KA_ARO10        VYYEMIKDKISCSSEWLDDINTACDQIDKVIKDIYKYSKPGYIFVPVDFVNQLVSITNLI     240
KS_ARO10        VYYEMIKDKLSCSSEWLDDINTACDQIDKVIKDIYKFSKPGYIFVPVDFVNKLVNIKNLI     240
                **::*:**:::**  :*:**:*****:*:**:****:*********.**.:  *.  **:

ARO10           NVPRISQQDCIVYPSENQLSDIINKITSWIYSSKTPAILGDVLTDRYGVSNFLNKLICKT     281
KA_ARO10        QEPHISMDTCLSEPSQQVSTEITDLILKWMYESKTPGLIGDVLVDRYGANNSLNHFIKQT     300
KS_ARO10        QEPRISMDTCLSKPSSQVTTEITDLICKWVYESKTPALIGDVLVDRYGANKNLNQFIKQT     300
                : *:** : *:  **.:  ::* : * .*:*.****.::****.****..: **::* :*

ARO10           GIWNFSTVMGKSVIDESNPTYMGQYNGKEGLKQVYEHFELCDLVLHFGVDINEINNGHYT     341
KA_ARO10        KMWNFSTVNGKSIINESNPYYMGLYNGKEGASVVIERFLQCDLILNFGLDINEINHGHYT     360
KS_ARO10        KMWNFSTVNGKSIVDESNPYYMGLYNGKEGVSVVIERFLQCDLILNFGLDINEINHGHYT     360
                 :****** ***:::**** *** ****** . * *:*  ***:*:**:******:****

ARO10           FTYKPNAKIIQFHPNYIRLVDTRQGNEQMFKGINFAPILKELYKR-IDVSKLSLQYDSNV     400
KA_ARO10        FNYRKDVNIVEFHPDYIRFVETSTGKEKIFKDVNFVFILQELLEKTLDNDQLNFQYDPNV     420
KS_ARO10        FNYKKDANIVEFHPDYIRFVETSTGKEKIFNDVNFVFILQELLKKTSNNNRLNFKYDPKV     420
                *.*: :.:*::***:***:*:*  *:*::*:.:**. **:** ::  : .:*.::** :*

ARO10           TQYTNETMRLE-----DPTNGQSSIITQVHLQKTMPKFLNPGDVVVCETGSFQFSVRDFA     455
KA_ARO10        KAYSSDEVYPDVVDTDSKEKPQQQNITVDFLSKSLPNLLNPGDVCFSETGAIYFAIRDMV     480
KS_ARO10        KAYSSDEVYPDVIDTDLKG---KQNITVDYLSKSLPNLFNPGDVCFSETGAIYFAIRDMV     477
                . *:.: :  :           .. **  .*.*::*:::***** ..***:: *::**:.

ARO10           FPSQLKYISQGFFLSIGMALPAALGVGIAMQDHSNAHING---GNVKEDYKPRLILFEGD     512
KA_ARO10        FPSQVKYMSQGFYLSIGTALPAAFGVGIAMQDYPRCHITDEDDDSIPIDYKPRLILFEGD     540
KS_ARO10        FPNQFKYMSQGFYLSIGTALPAAFGVGIAMQDYPRCHITD--DESIPINYKPRLILFEGD     535
                **.*.**:****:**** *****:********: ..**..    .:  :***********

ARO10           GAAQMTIQELSTILKCNIPLEVIIWNNNGYTIERAIMGPTRSYNDVMSWKWTKLFEAFGD     572
KA_ARO10        GAAQMTIQEMTSMIRYQVPIELFIWNNDGYTVERAICGPTRSYNDIMPWNWTKLFEAFGD     600
KS_ARO10        GAAQMTVQEMTSMLRYKVPIELFIWNNDGYTVERAICGPTRSYNDIMPWNWTKLFEAFGD     595
                ******:**:::::: ::*:*::****:***:**** ********:* *:**********

ARO10           FDGKYTNSTLIQCPSKLALKLEELKNSNKRSGIELLEVKLGELDFPEQLKCMVEAAALKR     632
KA_ARO10        TDGKYSINTFIDTKTKLTDKFEQLKKKKERNIIELIEVKLGIMDYPKQLQSMVTAMQSQD     660
KS_ARO10        TGGEYSINTFIDTKAKLADKFEQLRKKKERNVIELMEVKLGVLDYPKQLQSMVTAMQSKH     655
                 .*:*: .*:*:  :**: *:*:*::.::*. ***:***** :*:*:**:.** *   :

ARO10           NKK*          635
KA_ARO10        ----          660
KS_ARO10        ----          655
```

Supplementary Figure 10.

```
KA_BAT1         MLSAYSATSKRLATATLNRAIPLCRCFASTSPSGRSLDSTRVKITRNPNPSSPKPNDELI    60
KS_BAT1         MLPAYSATSKRIATSTLYRGIPLYRSFASAS--NAPLDASRVKITKNPNPSKPRPNDELI    58
BAT1            ML--------------QRHSLKLGKFSIRTLATGAPLDASKLKITRNPNPSKPRPNEELV    46
BAT2            ------------------------------MTLAPLDASKVKITTTQHASKPKPNSELV     29
KA_BAT2         -------------------------------MSAPLDASKLVITSVETPSKPLPNDQLV     28
KS_BAT2         ------------------------------MSAAPLDASKLVVTPVETPSKPLPNDQLV     29
                                                    **:::: :*     *.* **.:*:

KA_BAT1         FGKTFTDHMLQIEWTKENGWADPQIVPYGPLVLDPSAAVFHYGFEAFEGLKAYRTPDNKI    120
KS_BAT1         FGKTFTDHMLQIEWTQEKGWADPEIIPYGPLTLDPSAAVFHYGFEAFEGLKAYRTPGNKI    118
BAT1            FGQTFTDHMLTIPWSAKEGWGTPHIKPYGNLSLDPSACVFHYAFELFEGLKAYRTPQNTI    106
BAT2            FGKSFTDHMLTAEWTAEKGWGTPEIKPYQNLSLDPSAVVFHYAFELFEGMKAYRTVDNKI    89
KA_BAT2         FGKTFTDHMLTIEWTQQDGWDNPQIKPYGPLVLDPSSVVFHYAFELFEGMKAYRTPDNKI    88
KS_BAT2         FGKTFTDHMLTIEWTQQNGWDSPQIKPYGPLVLDPSAVVFHYAFELFEGMKAYRTKDNKI    89
                **::******   *: :.**  *.* **  * ****: ****.** ***:*****  *.*

KA_BAT1         SLFRPDMNMKRMNKSAARICLPTFDGDEAIKLMGTLIEQDKHLVPTGQGYSLYLRPTIIG    180
KS_BAT1         ALFRPDMNMKRMNKSAARICLPTFNGDEIIKLMGKLIEQDKHLVPQGQGYSLYLRPTIIG    178
BAT1            TMFRPDKNMARMNKSAARICLPTFESEELIKLTGKLIEQDKHLVPQGNGYSLYIRPTMIG    166
BAT2            TMFRPDMNMKRMNKSAQRICLPTFDPEELITLIGKLIQQDKCLVPEGKGYSLYIRPTLIG    149
KA_BAT2         TLFRPEKNMERMNKSASRILLPNFDGEELIKLITKLIEQDKHLIPEGQGYSLYIRPTLIG    148
KS_BAT2         TLFRPEKNMERMNKSASRILLPNFDGEELIKLITKLIEQDKHLIPEGQGYSLYIRPTLIG    149
                ::***: ** ****** ** **.*: :* *.*  .**:*** *:* *:*****:***:**

KA_BAT1         TTPALGVSTPDKALLYVIASPVGPYYKTGFKAVKLEATDYATRAWPGGCGDKKLGANYAP    240
KS_BAT1         TTAALGVSTPDKALLYVIASPVGPYYKTGFKAVKLEATDYATRAWPGGCGDKKLGANYAP    238
BAT1            TSKGLGVGTPSEALLYVITSPVGPYYKTGFKAVRLEATDYATRAWPGGVGDKKLGANYAP    226
BAT2            TTAGLGVSTPDRALLYVICCPVGPYYKTGFKAVRLEATDYATRAWPGGCGDKKLGANYAP    209
KA_BAT2         TTTTLGVATPDKALLFVICSPVGPYYKTGFKAVRLEATNYATRAWPGGCGDKKLGANYAP    208
KS_BAT2         TTTTLGVATPDKALLFVICSPVGPYYKTGFKAVRLEATNYATRAWPGGCGDKKLGANYAP    209
                *:  ***.**..***:** .*************:****:********* ***********

KA_BAT1         CILPQLQAAERGYQQNLWLFGPEKNITEVGTMNVFFAFKDSTTGKKELVTAPLDGTILEG    300
KS_BAT1         CILPQLQAAERGYQQNLWLFGPEKNITEVGTMNVFFAFKDSTTGKKELVTAPLDGTILEG    298
BAT1            CILPQLQAAKRGYQQNLWLFGPEKNITEVGTMNVFFVFLNKVTGKKELVTAPLDGTILEG    286
BAT2            CVLPQLQAASRGYQQNLWLFGPNNNITEVGTMNAFFVFKDSKTGKKELVTAPLDGTILEG    269
KA_BAT2         CVLPQLQAAQRGYQQNLWLFGPEENITEVGTMNCFFVFKDSATGKKELVTAPLDGTILEG    268
KS_BAT2         CVLPQLQAAQRGYQQNLWLFGPEENITEVGTMNCFFVFKDLATGKKELVTAPLDGTILEG    269
                *:*******.************::********* **.* :  ******************

KA_BAT1         VTRDSILTLTRQNLDPNEWDINERYYTITEVEERAKKGELLEAFGAGTAAVVSPIKEIGW    360
KS_BAT1         VTRDSILTLTRQNLDPNEWEINERYYTISEVEERAKRGELLEAFGAGTAAVVSPIKEIGW    358
BAT1            VTRDSVLTLARDKLDPQEWDINERYYTITEVATRAKQGELLEAFGSGTAAVVSPIKEIGW    346
BAT2            VTRDSILNLAKERLEPSEWTISERYFTIGEVTERSKNGELLEAFGSGTAAIVSPIKEIGW    329
KA_BAT2         VTRDSILTLARTKLDANEWTISERYCNMKELKERADKGELVEAFGSGTAAIVSPIKEVGW    328
KS_BAT2         VTRDSILTLAKTKLDSNEWIISERYCTMKELKERADKGELVEAFGSGTAAIVSPIKEVGW    329
                *****:*.*:: .*: .** *.*** .: *:  *:..***:****:****:******:**

KA_BAT1         KGSDIQVPLIPGEQSGPLTKQVASWIADIQYGRTKHGNWSQIVADLN-        407
KS_BAT1         KGSDIQVPLTPGEQSGPLTKQVASWISDIQYGRTKHDNWSQIVADLN-        405
BAT1            NNEDIHVPLLPGEQCGALTKQVAQWIADIQYGRVNYGNWSKTVADLN*        393
BAT2            KGEQINIPLLPGEQTGPLAKEVAQWINGIQYGETEHGNWSRVVTDLN*        376
KA_BAT2         NGEPIFIPLLPGEQSGALTKQVAEWIGDIQYGRENFNNWSRVITEL--        374
KS_BAT2         NGEPIFIPLLPGEQSGALTKQVAQWIGDIQYGRENFNSWSRVITEL--        375
                :.. * :** **** * *:*:**.** .****. :...**: :::*
```

Supplementary Figure 11.

```
FAS2            --MKPEVEQELAHILLTELLAYQFASPVRWIETQDVFLKDFNTERVVEIGPSPTLAGMAQ    58
KA_FAS2         MVMKPEVEQELAHVLLTELLAYQFASPVRWIETQDVFLKDLNTERVVEIGPSPTLAGMAQ    60
KS_FAS2         MVMKPEVEQELAHVLLTELLAYQFASPVRWIETQDVFLKDLNTERVVEIGPSPTLAGMAQ    60
                  ***********:**************************:*******************

FAS2            RTLKNKYESYDAALSLHREILCYSKDAKEIYYTPDPSELAAKEEPAKEEAPAPTPAASAP    118
KA_FAS2         RTLKNKYESYDAAVSLQRQVLCYSKDAKEIYYTPDPSELATEEAAAPTPDPAQ---AATP    117
KS_FAS2         RTLKNKYESYDAAVSLQRQVLCYSKDAKEIYYTPDPAELATEEAEAT-PVPAA---AAVA    116
                *************:**:*::****************:***::*  *    **    *:.

FAS2            APAAAAPAPVAAAAPAAAAAEIADEPVKASLLLHVLVAHKLKKSLDSIPMSKTIKDLVGG    178
KA_FAS2         AAPVAAAAPVPAAAPVTAAAEVPDAPTTAGLILHVLVAQKLKKSLDSVPMSKTIKDLVGG    177
KS_FAS2         TPAAVAAPVVAAAAPVAAAADVPDAPTTAGLILHVLVAQKLKKSLDSIPMSKTIKDLVGG    176
                :  ..*   * ****.:***:: * *..*.*:******:********:************

FAS2            KSTVQNEILGDLGKEFGTTPEKPEETPLEELAETFQDTFSGALGKQSSSLLSRLISSKMP    238
KA_FAS2         KSTVQNEILGDLGKEFGTTPEKPEETPLDELAETFQDSFSGSLGKQSSSLISRLMSSKMP    237
KS_FAS2         KSTVQNEILGDLGKEFGTTPEKPEETPLDELAETFQDSFNGSLGKQSSSLISRLMSSKMP    236
                ****************************:********:*.*:********:***:*****

FAS2            GGFTITVARKYLQTRWGLPSGRQDGVLLVALSNEPAARLGSEADAKAFLDSMAQKYASIV    298
KA_FAS2         GGFTITVARKYLQSRWGLGNGRQDSVLLVALTNEPASRLGSEADAKSFLDEQAQKYASIS    297
KS_FAS2         GGFTITVARKYLQSRWGLGNGRQDSVLLIALTNEPASRLGSETDAKSFLDEQAQKYASIS    296
                *************:**** .****.***:**:****:*****:***:***. *******

FAS2            GVDLSSAAS-ASGAAGAGAAAGAAMIDAGALEEITKDHKVLARQQLQVLARYLKMDLDNG    357
KA_FAS2         GINLASAAAASAGGAGAGAGAGGATIDAAALEDLTKDNKILARQQLEVLARYLKMDLDNG    357
KS_FAS2         GINLASAAAASAGGAGAGAAAGGATIDAAALEDLTKDNKILARQQLEVLARYLKMDLDNG    356
                *::*:***: ::*.*****.**.* ***.***::***:*:******:*************

FAS2            ERKFLKEKDTVAELQAQLDYLNAELGEFFVNGVATSFSRKKARTFDSSWNWAKQSLLSLY    417
KA_FAS2         ERKYLKEKSAVLELQAQLDHITEEMGEFYVSSLTNDFSRKKARVFDSSWNWAKQSLLHLY    417
KS_FAS2         ERKYLKEKSTVGELQAQLDHITEEMGEFYISALTNNFSRKKARVFDSSWNWAKQSLLHLY    416
                ***:****.:* *******::. *:***::..::..*******.************* **

FAS2            FEIIHGVLKNVDREVVSEAINIMNRSNDALIKFMEYHISNTDETKGENYQLVKTLGEQLI    477
KA_FAS2         FEIIHGVLKNVDREVVSEAINIMNRSNDALIKFMEYHVSNTDVSKGENYQLVKTLGEQLI    477
KS_FAS2         FEIIHGVLKNVDREVVSEAINIMNRSNDALIKFMEYHVSHTDVSKGENYQLVKSLGEQLI    476
                *************************************:*:** :*********:******

FAS2            ENCKQVLDVDPVYKDVAKPTGPKTAIDKNGNITYSEEPREKVRKLSQYVQEMALGGPITK    537
KA_FAS2         ENCKQVLNVDPVYRDISKPTGPKTSIDKNGNIKYEEAPREQVRKFSQYVKEMAVGGPLTK    537
KS_FAS2         ENCKQVLNVDPVYRDIAKPTGPKTSIDKNGNIKYEEAPREQVRKFSQYVQEMAVGGPLTK    536
                *******:*****:*::*******:*******.*.* ***:***:****:***:***:**

FAS2            ESQPTIEEDLTRVYKAISAQADKQDISSSTRVEFEKLYSDLMKFLESSKEIDPSQTTQLA    597
KA_FAS2         EDQPTIEQDLTRVYKAISAQASEHSISDSTKLEFEKLYGELIKFLSNSKEIDHTQTTQLA    597
KS_FAS2         EEQPTIEQDLTRVYKAINAQASEHSISDSTKLEFEKLYGELIKFLSNSKEIDHTQTTRLA    596
                *.*****:*********.***.::.**.**::******.:*:***..***** :***:**

FAS2            GMDVEDALDKDSTKEVASLPNKSTISKTVSSTIPRETIPFLHLRKKTPAGDWKYDRQLSS    657
KA_FAS2         GVVNDDDLDKDSTKEVASLSNKSQVTGAISSTIPRETVPFLHLKTKVANGAWQYDRSSSK    657
KS_FAS2         GVVNDDDLDKDSTKEVASLSNKSQATGTISSTIPRETVPFLHIKTKVANGAWKYDRTSSK    656
                *:  :* ************ ***  : ::********:****::.*.  * *:***  *.

FAS2            LFLDGLEKAAFNGVTFKDKYVLITGAGKGSIGAEVLQGLLQGGAKVVVTTSRFSKQVTDY    717
KA_FAS2         IFIDGLEDAAVNGTTFKDKYVLITGAGQGSIGGEILQGLLQGGAKVIVTTNSFNKKNLDY    717
KS_FAS2         VFIDGLENAAVNGTTFKDKYVLITGAGKGSIGGEILQGLLQGGAKVIATTYSFNKDNLDY    716
                :*:****.**.**.*************:****.*:***********:.**  *.*.  **

FAS2            YQSIYAKYGAKGSTLIVVPFNQGSKQDVEALIEFIYDTEKNGGLGWDLDAIIPFAAIPEQ    777
KA_FAS2         FQSQYAKYGAKGSTLIVAPFNQGSKNDVEALIDYIYDDEKNGGLGWDLDAIIPFAAIPEN    777
KS_FAS2         FQSQYAKYGAKGSTLIVAPFNQGSKNDVVALVDYIYDDEKNGGLGWDLDAIIPFAAIPEN    776
                :** *************.*******:** **:::*** *********************:
```

```
FAS2       GIELEHIDSKSEFAHRIMLTNILRMMGCVKKQKSARGIETRPAQVILPMSPNHGTFGGDG   837
KA_FAS2    GIELEDIDSKSEFAHRIMLTNIYRIMGCVKKQKSAKGIETRPAQVILPMSPNHGTFGGDG   837
KS_FAS2    GIELEDIDSKSEFAHRIMLTNIYRIMGCVKKQKTAKGIETRPAQVILPMSPNHGTFGGDG   836
           *****.**************** *:*********:*:***********************

FAS2       MYSESKLSLETLFNRWHSESWANQLTVCGAIIGWTRGTGLMSANNIIAEGIEKMGVRTFS   897
KA_FAS2    LYSESKLSLETLFNRWHSESWANQLTVCGAIIGWTRGTGLMSGNNIIAEGIEKMGVRTFS   897
KS_FAS2    LYSESKLSLETLFNRWHSESWANQLTVCGAIIGWTRGTGLMSGNNIIAEGIEKMGVRTFS   896
           :*****************************************.*****************

FAS2       QKEMAFNLLGLLTPEVVELCQKSPVMADLNGGLQFVPELKEFTAKLRKELVETSEVRKAV   957
KA_FAS2    QKEMAFNLLGLLTPEVTQLCQKSPVMADLNGGLQYLKDLKSFTAKLRRELTETSEIRKAV   957
KS_FAS2    QKEMAFNLLGLLTPEVTQLCQKSPVMADLNGGLQYLKDLKNFTAKLRRELTETSEIRKAV   956
           ****************.:****************:: :**.******:**.****:****

FAS2       SIETALEHKVVNGNSADAAYAQVEIQPRANIQLDFPELKPYKQVKQIAPAELEGLLDLER   1017
KA_FAS2    SIETALEHKAVNGDKADAAYAEVEVQPRANIQLDFPELKPYKQVKELAAPELEGMLDLEK   1017
KS_FAS2    SIETALEHKAVNGDKADAAYAEVEVQPRANIQLDFPELKPYKQVKQLAAPELEGMLDLER   1016
           *********.***:.******:**:********************::*  ****:****:

FAS2       VIVVTGFAEVGPWGSARTRWEMEAFGEFSLEGCVEMAWIMGFISYHNGNLKGRPYTGWVD   1077
KA_FAS2    VIVVTGFSEVGPWGSSRTRWQMEAFGEFSLEGCVEMAWMMNLIKYHNGNLKGRPYTGWVD   1077
KS_FAS2    VIVVTGFSEVGPWGSSRTRWQMEAFGEFSLEGCVEMAWMMNLIKYHNGNLKGRPYTGWVD   1076
           *******:*******:****:*****************:*.:*.****************

FAS2       SKTKEPVDDKDVKAKYETSILEHSGIRLIEPELFNGYNPEKKEMIQEVIVEEDLEPFEAS   1137
KA_FAS2    AKTNEPVEDKDVKPMYEKYILDHAGIRLIEPELFNGYDPKKKQLVQEVIVEEDMEPFEAS   1137
KS_FAS2    AKTNEPVEDKDVKPMYEKYILDHAGIRLIEPELFNGYDPKKKQMVQEIIVEEDMEPFEAS   1136
           :**:***:*****  **. **:*:*************:*:**:::**:*****:******

FAS2       KETAEQFKHQHGDKVDIFEIPETGEYSVKLLKGATLYIPKALRFDRLVAGQIPTGWNAKT   1197
KA_FAS2    KETAEQFKHEHGDKVDIFEIPETGEFSVRLLKGATLFVPKALRFDRLVAGQVPTGWDAKT   1197
KS_FAS2    KETAEQFKHEHGDRVDIFEIPETGEFSVKLLKGATLFVPKALRFDRLVAGQVPTGWDAKT   1196
           *********:***:***********:**:*******::*************:****:***

FAS2       YGISDDIISQVDPITLFVLVSVVEAFIASGITDPYEMYKYVHVSEVGNCSGSGMGGVSAL   1257
KA_FAS2    YGISEDTISQVDPITLFVLVSVAEAFIAAGITDPYEMYEYVHVSEVGNCSGSGMGGVSAL   1257
KS_FAS2    YGISEDTISQVDPITLFVLVSVAEAFIAAGITDPYEMYEYVHVSEVGNCSGSGMGGVSAL   1256
           ****:* ***************.*****:*********:*********************

FAS2       RGMFKDRFKDEPVQNDILQESFINTMSAWVNMLLISSSGPIKTPVGACATSVESVDIGVE   1317
KA_FAS2    RGMFKDRYKDLPVQNDILQESFINTMSAWVNMLLISSSGPIKTPVGACATAVESVDIGAE   1317
KS_FAS2    RGMFKDRYKDLPVQNDILQESFINTMSAWVNMLLISSSGPIKTPVGACATAVESVDIGAE   1316
           *******:** ***************************************:*******.*

FAS2       TILSGKARICIVGGYDDFQEEGSFEFGNMKATSNTLEEFEHGRTPAEMSRPATTTRNGFM   1377
KA_FAS2    TILSGKAKICIVGGYDDFQEEGSYEFANMKATSNTLEEYEHGRTPAEMSRPATTTRSGFM   1377
KS_FAS2    TILSGKAKICIVGGYDDFQEEGSYEFANMKATSNTLEEYEHGRTPAEMSRPATTTRSGFM   1376
           *******:***************:**.***********:*****************.***

FAS2       EAQGAGIQIIMQADLALKMGVPIYGIVAMAATATDKIGRSVPAPGKGILTTAREHHSSVK   1437
KA_FAS2    EAQGSGIQVIMTAELALKMGVPIYGIVALTATATDKIGRSVPAPGKGILTTAREHHGSLK   1437
KS_FAS2    EAQGSGIQVIMTADLALKMGVPIYGIVALTATATDKIGRSVPAPGKGILTTAREHHGSLK   1436
           ****:***:** *:**************::**************************.*:*

FAS2       YASPNLNMKYRKRQLVTREAQIKDWVENELEALKLEAEEIPSEDQNEFLLERTREIHNEA   1497
KA_FAS2    YASPLLDIKYRRRQLTKREVQIKQWVEDELELLQVEIEEIPEADQAEFVAERTKEIKLEG   1497
KS_FAS2    YASPLLDIKYRRRQLSNREVQIKQWVEDELELLQVEIEEIPEVDQAEFIAERTKEIKLEG   1496
           **** *::***:*** .**.***:***:*** *::* ****. ** **: ***:**: *.

FAS2       ESQLRAAQQQWGNDFYKRDPRIAPLRGALATYGLTIDDLGVASFHGTSTKANDKNESATI   1557
KA_FAS2    EKQLKAAQAQWGNEFYKRYPRIAPLRGALATYGLTIDDLGVASFHGTSTKANDKNESATI   1557
KS_FAS2    EKQLKAAQAQWGNEFYKRYPRIAPLRGALATYGLTIDDLGVASFHGTSTMANDKNESATI   1556
           *.**:*** ****:**** ****************************** **********

FAS2       NEMMKHLGRSEGNPVIGVFQKFLTGHPKGAAGAWMMNGALQILNSGIIPGNRNADNVDKI   1617
KA_FAS2    NEMMKHLGRSEGNPVFGVFQKFLTGHPKGAAGAWMLNGALQILNTGIVPGNRNADNIDKL   1617
KS_FAS2    NEMMKHLGRSEGNPVFGVFQKFLTGHPKGAAGAWMLNGALQILNSGIVPGNRNADNIDKL   1616
```

```
                ***************:*******************:********:**:********:**:

FAS2            LEQFEYVLYPSKTLKTDGVRAVSITSFGFGQKGGQAIVVHPDYLYGAITEDRYNEYVAKV    1677
KA_FAS2         LEQFEYVLYPSKTLKTNGVKAVSVTSFGFGQKGGQAIVVHPDFLYGAIDESRYNDYVKKV    1677
KS_FAS2         LEQFEYVLYPSKTLKTNGVKAVSVTSFGFGQKGGQAIVIHPDFLYGAIDESRYNEYAKKV    1676
                ****************:**:***:**************:***:***** *.***:*. **

FAS2            SAREKSAYKFFHNGMIYNKLFVSKEHAPYTDELEEDVYLDPLARVSKDKKSG-SLTFNSK    1736
KA_FAS2         TIREKSAYQFFHTGMTQNKIFISKEHAPYTDELEESVYLDPLARVSAQPKSGNELVFNKK    1737
KS_FAS2         AIREKSAYQFFHTGMTQNKIFISKEHAPYSDELEESVYLDPLARVSAEPKSGNELVFNKK    1736
                : ******:***.**  **:*:*******:*****.********** : *** .*.**.*

FAS2            NIQSKDSYINANTIETAKMIENMTK--EKVSNGGVGVDVELITSINVENDTFIERNFTPQ    1794
KA_FAS2         SIQNETSYEKNAAT--ANIVKSLTAEIAGGDDNGVGVDVELIQSINVDNETFIERNFTAA    1795
KS_FAS2         AIQSESSYEKNAAT--ANVVKSLTAEIAGGDDNGVGVDVELIQSINLDNETFIERNFTGS    1794
                 **.: ** :  :   *::::.:*      .:.********* ***::*:********

FAS2            EIEYCSAQPSVQSSFAGTWSAKEAVFKSLGVKSLGGGAALKDIEIVRVNKNAPAVELHGN    1854
KA_FAS2         EIEYCQKQPSIQSSFAGTWSAKEAVFKSLGVKSQGAGASLKDIEITRVNGNGPEVVLTGN    1855
KS_FAS2         EIEYCQRQPSIQSSFAGTWSAKEAVFKSLGVKSQGAGASLKDIEITRVNGNGPEVVLTGN    1854
                *****. ***:********************** *.**:******.*** *.* * * **

FAS2            AKKAAEEAGVTDVKVSISHDDLQAVAVAVSTKK*    1887
KA_FAS2         AKKIATEAGVKSVKVSISHDDFQSVAVAISSKN-    1888
KS_FAS2         AKKIASEAGVKSVKVSISHDDFQSVAVAISSKK-    1887
                *** * ****..*********:*:****:*:*:
```

Supplementary Figure 12.

```
TOR1          ------------------------------------MEPHEEQIWKSKLLKAANNDMDMDR    25
KA_TOR1       MMSFSGSATPFDSVNGSQPSSNIMSQILVRDMSTTLTESVEELFKHGSNISS--PY-YQE    57
KS_TOR1       MMSFSGSATPFDSANESQPSSNVMSQIVVRDMSTTLTESVEELFKYGSSISS--TY-YQE    57
                                                    :    *:::*     ::       :.

TOR1          NVPLAPNLNVNMNMKMNASRNGDEFGLTSSRFDGVVIGSNGDVNFKPILEKIFRELTSDY    85
KA_TOR1       NNISSSNIAAQMT-----------FDLMSQ--PSLNMGDR--ASALSTLDNIINSLKVKG    102
KS_TOR1       NTISSSNIAAQMT-----------FDLMSQ--PSLNMGDR--SSVLTTLDNIINSLKVKS    102
              *    : *: .:*.            *.* *.    .: :*..    .     *::*:..*. .

TOR1          KEERKLASISLFDLLVSLEHELSIEEFQAVSNDINNKILELVHTK-----KTSTRVGAVL    140
KA_TOR1       FEEQKSTVRYLESFLGLLARESNIEELKFYEKYINKKVIELVSNKTYSKKSINEKIGGVI    162
KS_TOR1       FEEQKSTVRYLESFLGLLARESNIEELKFYEKYINKKIIDLVSNKTNSKKSINEKIGGVI    162
               **:* :   *  .:*  * :* .***::  .: **:*:::** .*      . . ::*.*:

TOR1          SIDTLISFYAYTERLPNETSRLAGYLRGLIPSNDVEVMRLAAKTLGKLAVPGGTYTSDFV    200
KA_TOR1       AIQCLIRFYSQSEDIPNNIQKLVSALRSLLSCPNIEVIRLVTQTLGNLSQPGGPLISDYV    222
KS_TOR1       AIQCLIRFYSQSEDIPNNIQKLVGALRSVLACPNIEVIRLVTQTLGSLSQPGGPLISDYV    222
              :*: ** **: :* :**: .:*.. **.:: . ::**:**.::***.*: ***    **:*

TOR1          EFEIKSCLEWLTASTEKNSFSSSKPDHAKHAALLIITALAENCPYLLYQYLNSILDNIWR    260
KA_TOR1       EDEIKTGVDWLVSSSEKSS---SRQENKKHTAILILLTIAINSPYSIFPHINVILDNIWK    279
KS_TOR1       EDEIKTGVDWLVSSSEKSS---SRQENKKHTAILILLTIAINSPYSIFPHINIILDNIWK    279
              * ***: ::**.:*:**.*    *: :: **:*:**: ::* *.** :: ::* ******:

TOR1          ALRDPHLVIRIDASITLAKCLSTLRNRDPQLTSQWVQRLATSCEYGFQVNTLECIHASLL    320
KA_TOR1       ALKDSNKELRIDAANTMKQCLLIIERRDKTMFSKWITSFLSKCTTELNTNNMDTIHACLL    339
KS_TOR1       ALKDSNKELRIDAANTMKQCLLIIERRDMAIFSKWITSFLSKCTSELNTSNMDTIHACLL    339
              **:* :  :****: *: :**  :..**  : *:*:  : :.*   ::...:: ***.**

TOR1          VYKEILFLKD-PFLNQVFDQMCLNCIAYENHKAKMIREKIYQIVPLLASFNPQLFAGKYL    379
KA_TOR1       VYKVLISLNEHELIGKAFNDIFNNTWSFIDSKISYIRFETYQLFTLLSIFDSAVFSENYL    399
KS_TOR1       VYKVLISLNEHELIGKAFNDIFNNTWKFIDSKISYIRFETYQLFTLLSLFNPTVFSKNYL    399
              *** :: *::  ::.:.*:::  *    : : * . ** : **:. **: *:  :*: :**

TOR1          HQIMDNYLEILTNAPANKIPHLKDDKPQILISIGDIAYEVGPDIAPYVKQILDYIEHDLQ    439
KA_TOR1       NRVMINYLSKLQMLNKATSYIHKVDHPILIRSIGDIALHLGNDILPYLHSIVEILNNDLN    459
KS_TOR1       NQVMINYLSKLQMVNKSTSYIHKVDHPILIRSIGDIALHLGHDILPYLHSIVEILNNDLN    459
              :::* ***. *       .     * *:* :: ****** .:* ** **::.*:: :::**:

TOR1          TKFKFRKKFENEIFYCIGRLAVPLGPVLGKLLNRNILDLMFKCPLSDYMQETFQILTERI    499
KA_TOR1       MKYKNRITFEKEIFYCIARLVEGTESQMIVYLQGGLLESMLECPLTDYMQFTLQVITSKI    519
KS_TOR1       LKYKNRITFEKEIFYCIARLVEGTELEMIVYLQGGLLESMLECPLTDYMQFTLQIITSKI    519
               *:* * .**:******.**.       :    *: .:*: *::***:**** *:*::*.:*

TOR1          PSLGPKINDELLNLVCSTLSGTPFIQPGSPMEIPSFSRERAREWRNKNILQKTGESNDDN    559
KA_TOR1       PILESVISDKLLDLISLRLSGATFKLPGSPEESQLFSLQGARNWRNENEFRKKNLLNDDE    579
KS_TOR1       PILESVISDKLLDLISLRLSSTTFKLPGSPEESQLFSLQGARNWRNENEFRKKNVTNDDE    579
              * *   *.*:**:*:.  **.: *  **** *   ** : **:***:* ::*..  ***:

TOR1          NDIKIIIQAFRMLKNIKSRFSLVEFVRIVALSYIEHTDPRVRKLAALTSCEIYVKDNICK    619
KA_TOR1       NDTKIIIQSLRMLLNIDYKYQMSEFVRTTIICYIEHEDPRVRKLAALTSCHLLIKDNIGR    639
KS_TOR1       NDTKIIIQSLRMLLNIDYKYQMAEFVRTTIICYIEHEDPRVRKLAALTSCHLLIKDNIGR    639
              ** *****::*** **. ::.: **** . :.**** ************.: :**** :

TOR1          QTSLHSLNTVSEVLSKLLAITIADPLQDIRLEVLKNLNPCFDPQLAQPDNLRLLFTALHD    679
KA_TOR1       QTSLNSLNIVSEVLSKLLTVAITDINPEIRLQILEHMDHSFDPHLSQPENSRLLFMLLED    699
KS_TOR1       QTSLNSLNIVSEVLSKLLSVAITDINPEIRLQILEHIDHTFDPHLSQPENSRLLFMLLED    699
              ****:*** *********:::*:*   :***::*::::  ***:*:**:* ****  *.*

TOR1          ESFNIQSVAMELVGRLSSVNPAYVIPSIRKILLELLTKLKFSTSSREKEETASLLCTLIR    739
KA_TOR1       EVFIVRVSTLKILGRLTSVNPAYIVPLLRNTLLELLTELKYLKLPNQNEECLTMLCTLIS    759
KS_TOR1       EVFIVRVSTLKILGRLTSVNPAYIVPLLRSTLLELLTELKYLKLPNQNEECLTMLCTLIS    759
              * * ::  ::::***:******::* :*. ******:**:  .   .::**  ::*****
```

```
TOR1          SSKDVAKPYIEPLLNVLLPKFQDTSSTVASTALRTIGELSVVGGEDMKIYLKDLFPLIIK   799
KA_TOR1       STKEITKPYIEPMLDILLEKLEDSSSSVVSSALRTIGELSIVGGEGMKDYLDKLMPPIIE   819
KS_TOR1       STKEITKPYIEPMLDILLEKLEDSSSSVVSSALRTIGELSVVGGEDMKDYLHKLMPPIIE   819
              *:*:::******:*::** *::*:**:*.*:*********:****.** **..*:* **:

TOR1          TFQDQSNSFKREAALKALGQLAASSGYVIDPLLDYPELLGILVNILKTENSQNIRRQTVT   859
KA_TOR1       AFKDQSNSYKRSAALRTLRQLSSASGYVIDPLLDYSELLNVLINILKSDASQHIKQETVS   879
KS_TOR1       AFKDQSNSYKRSAALRTLRQLSSASGYVIDPLLDYSELLNVLINILKSDASQHIKQETVS   879
              :*:*****:**.***::* **::::********** ***.:*:****:: **:*:::**:

TOR1          LIGILGAIDPYRQKEREVTSTTDISTEQNAPPIDIALLMQGMSPSNDEYYTTVVIHCLLK   919
KA_TOR1       LLGTLGALDPYKYREVEETAEETTTLEQNSIPIDISLLMQNVSPSNEEYYPTIVINTLLK   939
KS_TOR1       LLGTLGALDPYKYREVEETAEETTTLEQNAIPIDISLLMQNISPSNEEYYPTIVVNTLLK   939
              *:* ***:***: :* * *:     : ***: ****:****.:****:*** *:*:: ***

TOR1          ILKDPSLSSYHTAVIQAIMHIFQTLGLKCVSFLDQIIPTILDVMRTCSQSLLEFYFQQLC   979
KA_TOR1       ILKDSSLSMHHPAVVQTIVNLFEILKLRCVAFLGQTIPAIISLLSSCPNSLLEYYLQQLG   999
KS_TOR1       ILKDSSLSMHHPAVVQTIVNLFETLKLRCVSFLGQTIPAIISLLSLCPNSLLEYYLQQLG   999
              **** *** :* **:*:*:::*: * *:**:**.* **:*:.::  * :****:*:***

TOR1          SLIIIVRQHIRPHVDSIFQAIKDFSSVAKLQITLVSVIEAISKALEGEFKRLVPLTLTLF   1039
KA_TOR1       VLISIVKQHIRPYVDDIFNSIALHFEDYKLQLTLISVITSLASSLKGEFKRFVPSVLTYF   1059
KS_TOR1       ILISIVKQHIRPYVDDIFNTIESRFEDYKLQLTIISVINSLASSLKGEFKRFVPSVLTYF   1059
               ** **:*****:**.**::*    .  ***:*::*** :::.:*:*****:**  .** *

TOR1          LVILENDKSSDKVLSRRVLRLLESFGPNLEGYSHLITPKIVQMAEFTSGNLQRSAIITIG   1099
KA_TOR1       MSIIDKDKSKDKSVTNAIFGCLIVLGDNLDEYLYLIIPSIIKISEVTHGSIKKASIITLG   1119
KS_TOR1       MGIIDKDKCKDKSVAKAIFGCLIILGNNLDEYLYLIIPSIIRISEVTHGSIKKASIITLG   1119
              : *:::**..** ::. ::  *   :* **: * :** *.*::::*.* *.:::::***:*

TOR1          KLAKDVDLFEMSSRIVHSLLRVLSSTTSDELSKVIMNTLSLLLIQMGTSFAIFIPVINEV   1159
KA_TOR1       KLAKINNMSTMSSRIIHACFRLLYSKDS-EVIRLTMNLLCLLLLHLKSDFSVFIPTINTL   1178
KS_TOR1       KLAKINNMSTMSSRIIHACFRLLYSKDP-EVIRLTMNLLCLLLLQLKSDFSVFIPTLNTL   1178
              ****  ::  *****:*: :*:* *.   *: :: ** *.***::: :.*::***.:* :

TOR1          LMKKHIQHTIYDDLTNRILNNDVLPTKILEANTTDYKPAEQMEAADAGVAKLPINQSVLK   1219
KA_TOR1       LIRNNIQHNVYDQLVNKLLHNEMLPSALVFGLDYDDNQ-IVEQRKDQGLEKLNVNQSLLK   1237
KS_TOR1       LIKNNIQHNVYDQLVNKLLNNEILPSTLIFGLDYEDTQ-VVDQRKDQALEKLSVNQSLLK   1237
              *:::::***.:**:*.*::*::**: :: .    : .    :  * .: ** :***:**

TOR1          SAWNSSQQRTKEDWQEWSKRLSIQLLKESPSHALRACSNLASMYYPLAKELFNTAFACVW   1279
KA_TOR1       SMWDCSQQRTKEDWQEWLKRLSIQMLKESPSHALRACAGLASVYLPLARDLFNASFSSVW   1297
KS_TOR1       SMWDCSQQRTKEDWQEWLKRLSIQMLKESPSHALRVCAGLASVYLPLARELFNASFSSVW   1297
              * *:.************ ******:*********.*:.***:* ***::***::*:.**

TOR1          TELYSQYQEDLIGSLCIALSSPLNPPEIHQTLLNLVEFMEHDDKALPIPTQSLGEYAERC   1339
KA_TOR1       TELYTQYQEDFIQSLCLALSSPQNPPEIYQTLLNLVEFMEHDDKSLPISSQTLGEYAEKC   1357
KS_TOR1       TELYTQYQEDFIQSLCLALSSPQNPPEIYQTLLNLVEFMEHDDKSLPISSQTLGEYAEKC   1357
              ****:*****:* ***:***** *****:***************:*** :*:******:*

TOR1          HAYAKALHYKEIKFIKEPE-NSTIESLISINNQLNQTDAAIGILKHAQQHHSLQLKETWF   1398
KA_TOR1       HAYAKALHFKEAKFLQEPLDNATVESLISINNQIYQNDAAVGILKFAQKNNNLQLQESWY   1417
KS_TOR1       HAYAKALHFKEAKFLQEPLDNATVESLISINNQIYQNDAAVGILKFAQKNNNLQLQESWY   1417
              ********:** **::**  *:*:*********: *.***:****.**::::.***:*:*:

TOR1          EKLERWEDALHAYNEREKAGDTSVSVTLGKMRSLHALGEWEQLSQLAARKWKVSKLQTKK   1458
KA_TOR1       EKLRRWEDALAAYSKRTEAGDTSPSVTLGHMRSLYALSDWDNLAKFSAERWDDASVTLQK   1477
KS_TOR1       EKLRRWEDALAAYSKRTEAGDTSPSVTLGHMRSLYALSDWNNLAKFSAERWDDASASLQK   1477
              ***.****** **.:* :***** *****:****:**.:*::*:::*:*:.:*. :.   :*

TOR1          LIAPLAAGAAWGLGEWDMLEQYISVMKPKSPDKEFFDAILYLHKNDYDNASKHILNARDL   1518
KA_TOR1       QMAPLAANGAWSLGQWDKIDQYISVMKNKTPDKEFFSAILSIHNDEFDSAKEHIYNARDL   1537
KS_TOR1       QMAPLAANGAWSLGQWDKINQYISVMKNKTPDKEFFSAILSIHNDDFDSAKDHIYNARDL   1537
               :*****..**.**:** :: :*******:*:******.*** :*::::*.*..** *****

TOR1          LVTEISALINESYNRAYSVIVRTQIITEFEEIIKYKQLPPNSEKKLHYQNLWTKRLLGCQ   1578
KA_TOR1       LVTELSALVNESYNRAYNVIVRTQLITELEEVIEYKRLPANSEKRAVYNSAWNKRLLGCQ   1597
KS_TOR1       LVTEISALVNESYNRAYNVIVRTQLITELEEVIEYKKLPPNSDKRAVYNSTWNKRLLGCQ   1597
```

```
                    ****:***:********.******:***:**:*:**:** **:*:  *:. *.*******

TOR1                KNVDLWQRVLRVRSLVIKPKQDLQIWIKFANLCRKSGRMRLANKALNMLLEGGNDPSLPN     1638
KA_TOR1             KNVDIWQRALKVRSLVISPKQDMHIWIKFANLCRKSGKLNLAEAALSALLEDTSDISPSL     1657
KS_TOR1             KNVDIWQRALKVRSLVISPKQDMHIWIKFANLCRKSGKLNLAEAALSALLEDTTDISPAL     1657
                    ****:***.*:******.****::*************::.**: **. ***. .* *

TOR1                TFKAPPPVVYAQLKYIWATGAYKEALNHLIGFTSRLAHDLGLDPNNMIAQSVKLSSASTA     1698
KA_TOR1             PAKAPPPVVYSQLKYLWATGSRQEALRYLIGFTSRMAHDLGLDPSNMIAQSVSQNQSLSP     1717
KS_TOR1             PAKAPPPVVYSQLKYLWATGSRQEALRYLIGFTSRMAHDLGLDPSNMIAQSVSQNQSLSP     1717
                      ********:****:****: :***.:*******:********.*******. ..: :

TOR1                PYVEEYTKLLARCFLKQGEWRIATQPNWRNTNPDAILGSYLLATHFDKNWYKAWHNWALA     1758
KA_TOR1             SNIEEYTKLLARCFLKQGEWRVTMQPNWREENPDAILGSYLLATHFDNKWYKAWHNWALV     1777
KS_TOR1             SNIEEYTKLLARCFLKQGEWRVTMQPNWREENPDAILGSYLLATHFDNRWYKAWHNWALV     1777
                      :******************:: *****: ****************:.**********.

TOR1                NFEVISMVQEETKLNGGKNDDDDDTAVNNDNVRIDGSILGSGSLTINGNRYPLELIQRHV     1818
KA_TOR1             NFEVISAVTSGKKPDNGTSTDVSKSNQ-------KHGNIQSDILKIEGREYSMELIQRHV     1830
KS_TOR1             NFEVISAVTSGKKPDNAASNDTSKPNQ-------KHGSIQNDIIKIEGREYSMELIQRHV     1830
                    ****** * . .* :.. . * ..          . . : .. :.*:*..* :*******

TOR1                VPAIKGFFHSISLLETSCLQDTLRLLTLLFNFGGIKEVSQAMYEGFNLMKIENWLEVLPQ     1878
KA_TOR1             IPAIKGFFHSISLSKTSSLQDTLRLLTLWFTFGGTSEAGQAMHAGFNLIKIDSWLEVLPQ     1890
KS_TOR1             IPAIKGFFHSISLSKTSSLQDTLRLLTLWFTFGGTSEAGQAMYAGFNLIKIDSWLEVLPQ     1890
                    :************ :**.********** *.*** .*..***: ****:**:.*******

TOR1                LISRIHQPDPTVSNSLLSLLSDLGKAHPQALVYPLTVAIKSESVSRQKAALSIIEKIRIH     1938
KA_TOR1             LISRIHQPDEAVSRSLLALLSDIGKAHPQVMVNPLIVAIKSESVSRQKAALSIIDKMKIH     1950
KS_TOR1             LISRIHQPDEAVSRSLLALLSDLGKAHPQVMVNPLIVAIKSESVSRQKAALSIIDKMKIH     1950
                    ********* :**.***:****:******.:* ** ******************:*::**

TOR1                SPVLVNQAELVSHELIRVAVLWHELWYEGLEDASRQFFVEHNIEKMFSTLEPLHKHLGNE     1998
KA_TOR1             STKLVEQAELVSNELIRVAVLWHELWYEGLEDASRQFFGEHNTEKMFATLEPLHELLKRT     2010
KS_TOR1             STRLVEQAELVSNELIRVAVLWHELWYEGLEDASRQFFGEHNTEKMFATLEPLHELLKKS     2010
                    *  **:******:************************* *** ****:******: * .

TOR1                PQTLSEVSFQKSFGRDLNDAYEWLNNYKKSKDINNLNQAWDIYYNVFRKITRQIPQLQTL     2058
KA_TOR1             PETIRELSFQNAFGRDLTDAYEWVLNFKRSKDVSNLNQAWDIYYNVFRRISRQLPQLQTL     2070
KS_TOR1             PETIRELSFQNAFGRDLTDAYEWVLNFKRSKDVSNLNQAWDIYYNVFRRISRQLPQLQTL     2070
                    *:*: *:***::*****.*****: *:*:***:.**************:*:**:******

TOR1                DLQHVSPQLLATHDLELAVPGTYFPGKPTIRIAKFEPLFSVISSKQRPRKFSIKGSDGKD     2118
KA_TOR1             NLHHVSPKLLDARDLEMAVPGSYSVGKPIITISYFDPIFSVISSKQRPRKFTIRGSDGKD     2130
KS_TOR1             NLRHVSPKLLDARDLEMAVPGSYSAGKPIIRISYFDPIFSVISSKQRPRKFTIRGSDGKD     2130
                    :*:****:** ::***:****:*  *** * *: *:*:*************:*:******

TOR1                YKYVLKGHEDIRQDSLVMQLFGLVNTLLKNDSECFKRHLDIQQYPAIPLSPKSGLLGWVP     2178
KA_TOR1             YQYVLKGHEDIRQDSLVMQLFGLVNTLLEHDSECFKRHLDIQKYPAIPLSPKSGLLGWVP     2190
KS_TOR1             YQYVLKGHEDIRQDSLVMQLFGLVNTLLEHDSECFKRHLDIQKYPAIPLSPKSGLLGWVP     2190
                    *:**************************::************:*****************

TOR1                NSDTFHVLIREHRDAKKIPLNIEHWVMLQMAPDYENLTLLQKIEVFTYALDNTKGQDLYK     2238
KA_TOR1             NSDTFHVLIRDHRDANKVPLNIEHWVMLQMAPDYDNLTLLQKIEVFQYAMDNTKGQDLAQ     2250
KS_TOR1             NSDTFHVLIRDHRDANKVPLNIEHWVMLQMAPDYDNLTLLQKIEVFQYAMDNTKGQDLAQ     2250
                    **********:****:*:****************:*********** **:******** :

TOR1                ILWLKSRSSETWLERRTTYTRSLAVMSMTGYILGLGDRHPSNLMLDRITGKVIHIDFGDC     2298
KA_TOR1             VLWLKSRSSETWLDRRTVYSRSLAVMSMVGYILGLGDRHPSNLMLDRITGKVIHIDFGDC     2310
KS_TOR1             VLWLKSRSSETWLDRRTVYSRSLAVMSMVGYILGLGDRHPSNLMLDRITGKVIHIDFGDC     2310
                    :************:***.*:********.*******************************

TOR1                FEAAILREKYPEKVPFRLTRMLTYAMEVSGIEGSFRITCENVMRVLRDNKESLMAILEAF     2358
KA_TOR1             FEAAILREKFPEKVPFRLTRMLIKALEVSGIEGSFRITCEHVMRVLRNNKESLMAILEAF     2370
KS_TOR1             FEAAILREKFPEKVPFRLTRMLIKALEVSGIEGSFRITCEHVMRVLRNNKESLMAILEAF     2370
                    *********:************  *:**************:******:************

TOR1                ALDPLIHWGFDLPPQKLTEQTGIPLPLINPSELLRKGAITVEEAANMEAEQQNETKNARA     2418
```

```
KA_TOR1      AFDPLIHWGFDFPTDKIMEETGIRLPMVNPSELLRKGAITVSEANKMEEEQQIEIRNARA   2430
KS_TOR1      AFDPLIHWGFDFPTDKIMEETGIQLPMVNPSELLRKGAITVSEANKMEEQQQIEIRNARA   2430
             *:*********:* :*: *:*** **::*************.** :** :** * :****

TOR1         MLVLRRITDKLTGNDIKRFNELDVPEQVDKLIQQATSIERLCQHYIGWCPFW*  2470
KA_TOR1      LLVLKRITDKLTGNDIPRFKNLDIPDQVDKLTKEAMSIENLCQHYVGWCPFW-  2482
KS_TOR1      LLVLKRITDKLTGNDIPRFESLDIPDQVDKLTKEAMSIENLCQHYVGWCPFW-  2482
             :***:*********** **:.**:*:***** ::* ***.*****:******
```

Supplementary Figure 13a. Multiple alignment of the amino acid sequences of Gcn4 from *S. cerevisiae* (S288C) and their *Kazachstania* spp. (*K. aerobia* (KA) and *K. servazzii* (KS) orthologues. The alignments were performed using Clustal Omega software. Non-conserved residues are black text on white background, identical residues are black text on light grey background, and conserved residues in all sequences are black text on dark grey background.

```
GCN4                    --MSEYQP-SLFALNPMGFSPLDGSKSTNENVSASTSTAKPMVGQLIFDKFIKTEEDPII  57
KA_contig_17.g1561      --MSEFQLPSLFTK-NGSFEPSTESTAT--MT--TKINNDSQSAMVDFDQFIKKE--DE-  50
KS_contig_10.g21        MKMSEFQMPSLFTK-TGSFESSLNT--E--LL--KKNNNNSQPAMVDFDHFIKKEQEGD-  52
                          ***:*  ***:    .*.    :         .. . .   . : **:***.*

GCN4                    KQDTPSNLDFDFALPQTATAPDAKTVLPIPELDDAVVESFFSSSTDSTPMFEYENL----  113
KA_contig_17.g1561      --------------------LINEDSLDLPELDSAVVDAFFASSNDSTPMFEYDTTPIQD  90
KS_contig_10.g21        --------------------LIDDSSLDLPELDSAVVDAFFASSNDSTPMFEYDTTPIQD  92
                                               .  * :****.***::**:**.********:.

GCN4                    ---EDNSKEWTSLFDNDIPVTT-DDVSLADKAIESTEEVSLVPSN-LEVSTTSFLPTPVL  168
KA_contig_17.g1561      SVASTHDHEWASLFDDDIPVITEDDVSLNDKAMQSTEVSSYDAFQSTINQIPSFLPTPVI  150
KS_contig_10.g21        SVAATNDHEWASLFDDDIPVITEDDVSLNDKAMQSTEVSSCDAFPSTINQIPSFLPTPII  152
                             :.:**:****:**** * ***** ***::***  *         .  ******::

GCN4                    EDAKLTQTRKVKKPN--SVVKKSHHVGKDDESRLDHLGVVAYNRKQRSIPLSPIVPESSD  226
KA_contig_17.g1561      EDAKLLKSTTSGRVSKKKSVSSSSSSSASSTPKIDHLGVITYNKKNRSIPLTPVIPESDD  210
KS_contig_10.g21        EDAKLSKT--TGRINKKSF--SSSSSSSSSTPKIDHLGVITYNKKSRSIPLTPVIPESDD  208
                        ***** ::    : .  .   .*   . ..  ::*****::**:*.*****:*::***.*

GCN4                    PAALKRARNTEAARRSRARKLQRMKQLEDKVEELLSKNYHLENEVARLKKLVGER*      281
KA_contig_17.g1561      PAALKRARNTEAARRSRARKLKRMTQLEDKVEELLNKNKSLENEVETLKRLLAERS      266
KS_contig_10.g21        PAALKRARNTEAARRSRARKLKRMNQLEDKVEELLNKNKSLESEVESLKKLLAERS      264
                        *********************:**.**********.**  **.**  **:*:.**
```

Supplementary Figure 13b. Multiple alignment of the amino acid sequences of Gcn4 from *S. cerevisiae* (EC1118) and their *Kazachstania* spp. (*K. aerobia* (KA) and *K. servazzii* (KS) orthologues. The alignments were performed using Clustal Omega software. Non-conserved residues are black text on white background, identical residues are black text on light grey background, and conserved residues in all sequences are black text on dark grey background.

```
CAY79156.1              --MSEYQP-SLFALNPMGFSPLDGSKSTNENVSASTSTAKPMVGQLIFDKFIKTEEDPII  57
KA_contig_17.g1561      --MSEFQLPSLFTK-NGSFEPSTESTAT--MT--TKINNDSQSAMVDFDQFIKKE--DE-  50
KS_contig_10.g21        MKMSEFQMPSLFTK-TGSFESSLNT--E--LL--KKNNNNSQPAMVDFDHFIKKEQEGD-  52
                          ***:*  ***:    .*.    :         .. . .   . : **:***.*

CAY79156.1              KQDTPSNLDFDFALPQTATAPDAKTVLPIPELDAAVVESFFSSSTDSTPMFEYENL----  113
KA_contig_17.g1561      --------------------LINEDSLDLPELDSAVVDAFFASSNDSTPMFEYDTTPIQD  90
KS_contig_10.g21        --------------------LIDDSSLDLPELDSAVVDAFFASSNDSTPMFEYDTTPIQD  92
                                               .  * :****:***::**:**.********:.

CAY79156.1              ---EDNSKEWTSLFDNDIPVTT-DDVSLADKAIESTEEVSLVPSN-LEVSTTSFLPTPVL  168
KA_contig_17.g1561      SVASTHDHEWASLFDDDIPVITEDDVSLNDKAMQSTEVSSYDAFQSTINQIPSFLPTPVI  150
KS_contig_10.g21        SVAATNDHEWASLFDDDIPVITEDDVSLNDKAMQSTEVSSCDAFPSTINQIPSFLPTPII  152
                             :.:**:****:**** * ***** ***::***   *        .  ******::

CAY79156.1              EDAKLTQTRKVKKPN--SVVKKSHHVGKDDESRLDHLGVVAFNRKQRSIPLSPIVPESSD  226
KA_contig_17.g1561      EDAKLLKSTTSGRVSKKKSVSSSSSSSASSTPKIDHLGVITYNKKNRSIPLTPVIPESDD  210
KS_contig_10.g21        EDAKLSKT--TGRINKKSF--SSSSSSSSSTPKIDHLGVITYNKKSRSIPLTPVIPESDD  208
                        ***** ::    : .  .   .*   . ..  ::*****:::*:*.*****:*::***.*

CAY79156.1              PAALKRARNTEAARRSRARKLQRMKQLEDEVEELLSKNYHLENEVARLKKLVGER-      281
KA_contig_17.g1561      PAALKRARNTEAARRSRARKLKRMTQLEDKVEELLNKNKSLENEVETLKRLLAERS      266
KS_contig_10.g21        PAALKRARNTEAARRSRARKLKRMNQLEDKVEELLNKNKSLESEVESLKKLLAERS      264
                        *********************:**.****:*****.**  **.**  **:*:.**
```

Supplementary Figure 14a.

```
KS_contig_25.g2372      MATN------SIKLIGSDGQLNI-------VKSIAKRLSIPITECVLIRDPNQEIVFSIS  47
ADH7                    --MLYPEKFQGIGISNAKDWKHPKLVSFDPKPFGDHDVDVEIEACGICGSDFHIAVGNWG  58
KA_contig_17.g1558      MTTNYPEEFQGFGIVDKDDWLHPKKINFKPKEFTPNSVDIKIECCGVCGSDIHCAKSDWS  60
KS_contig_10.g24        MTTNYPEEFQGFGIVDKDDWLHPKKINFKPKEFTPNSVDIKIECCGVCGSDIHCAKSDWS  60
ADH6                    --MSYPEKFEGIAIQSHEDWKNPKKTKYDPKPFYDHDIDIKIEACGVCGSDIHCAAGHWG  58
KS_contig_24.g2365      --MSYPEKFQGIAVLDHADYTHPKKVAFDPKPFHPSDIDIAIEACGVCGSDCHAANGGWG  58
KA_contig_21.g2255      --MSYPEKFQGIAVLDHADYTHPKKVSYDPKPFSPTDIDISIEACGVCGSDCHSANGGWG  58
KA_contig_19.g1873      --MSYPEKFQGIAVLDHADYTHPKKVSFDPKPFAPSDIDISIEACGVCGSDCHAANGGWG  58
KA_contig_3.g2902       --MSYPEKFQGIAVLDHADYTHPKKVSFDPKPFAPSDIDISIEACGVCGSDCHAANGGWG  58
                                  .: :  .   .   :             :.: *   * :   .   :     .

KS_contig_25.g2372      E-----TVRDQDVYIVAQIGSD-----KVNDR------VFELLIMINAAKAASARKITVV  91
ADH7                    PVPENQILGHEIIGRVVKVGSKCHTGVKIGDRVGVGAQALACFE---CER--------CK  107
KA_contig_17.g1558      ELAHGQIVGHEIIGTVTKIGPGCKSGLKLGDRVGVGAQVFSCLD---CDR--------CK  109
KS_contig_10.g24        ELSHGQVVGHEIIGTVAKIGPDCNTGLKIGDRVGVGAQVFSCLD---CDR--------CK  109
ADH6                    NMKMPLVVGHEIVGKVVKLGPKSNSGLKVGQRVGVGAQVFSCLE---CDR--------CK  107
KS_contig_24.g2365      DKPKPLVVGHEVIGRVVKIGPNCTTGLKLGDRVGVGAQVLSCLE---CNR--------CK  107
KA_contig_21.g2255      DKPKPLVVGHEVIGRVVKMGPNCTSGLKIGDRVGVGAQVLSCLE---CDR--------CK  107
KA_contig_19.g1873      DKPKPLVVGHEVIGRVVKMGPNCTSGLKIGDRVGVGAQVLSCLE---CDR--------CK  107
KA_contig_3.g2902       DKPKPLVVGHEVIGRVVKMGPNCTSGLKIGDRVGVGAQVLSCLE---CDR--------CK  107
                               : .: :  *.::*       *:.:*      .:  :    . :

KS_contig_25.g2372      IPNFPYARQDKKDKARAPITAKLMADMLTTAGCDHIVTMDLHAAQIQGFFDVPLDNLYAE  151
ADH7                    SDNEQYCTNDHVLTMWTPYK----DGYISQGGFASHV-------RLHEHFAIQIPENIPS  156
KA_contig_17.g1558      NNNEPYCPK-FVTTYSQPYE----DGYISQGGYANYV-------RVHEHFAIPIPEEIPS  157
KS_contig_10.g24        NDNEPYCPK-FVTTYSQPYE----DGYISQGGYANYI-------RVHEHFAVPIPEEIPS  157
ADH6                    NDNEPYCTK-FVTTYSQPYE----DGYVSQGGYANYV-------RVHEHFVVPIPENIPS  155
KS_contig_24.g2365      EDNEPYCPK-FVTTYGQDYE----DGYNSKGGYASHI-------RVHEHFVVPIPENIPS  155
KA_contig_21.g2255      ADNEPYCPK-FVTTYGQDYE----DGYNSKGGYASHI-------RVHEHFVVPIPENIPS  155
KA_contig_19.g1873      ADNEPYCPR-FVTTYGQDYE----DGYNSKGGYASHI-------RVHEHFVVPIPENIPS  155
KA_contig_3.g2902       ADNEPYCPK-FVTTYGQDYE----DGYNSKGGYASHI-------RVHEHFVVPIPEYIPS  155
                          *  *. .    .         .  : .*    :       ::: .* : : :   .

KS_contig_25.g2372      PSIVNYIKEHLDLNNCIIVSPDAGGTKRVASLA------------EDLNL-TFALIHKER  198
ADH7                    PLAAPLLCGGITVFS-PLLRNGCGPGKKRVGIVGIGGIGHMGILLAKAMGAEVYAFSRGHS  215
KA_contig_17.g1558      ELAAPLMCGGITVFS-PLLRNGCGPGKKVGIVGIGGIGHMGVIFAKALGAEVWAFSRKNN  216
KS_contig_10.g24        ELAAPLMCGGITVFS-PLLRNGCGPGKKVGIVGIGGIGHMGVIFAKALGAEVWAFSRKNN  216
ADH6                    HLAAPLLCGGLTVYS-PLVRNGCGPGKKVGIVGLGGIGSMGTLISKAMGAETYVISRSSR  214
KS_contig_24.g2365      ELAAPLLCGGLTVFS-PLVRNGCGPGKKVGILGIGGIGHMGVIFAKALGAEVYAISRSSA  214
KA_contig_21.g2255      HLAAPLLCGGLTVFS-PLVRNGCGPGKKVGVLGIGGIGHMGVLFAKALGAEVYAISRTSA  214
KA_contig_19.g1873      HLAAPLLCGGLTVFS-PLVRNGCGPGKKVGVLGIGGIGHMGVLFAKALGAEVYAISRTSA  214
KA_contig_3.g2902       HLAAPLLCGGLTVFS-PLVRNGCGPGKKVGILGIGGIGHMGVIFAKALGAEVYAISRTSA  214
                           .   :   : :  .  ::  ..*  *:*. :.              : :.  .:.: :

KS_contig_25.g2372      VKANEVSRMVLVGDVTG---KTCIIVDDMADTCGTLVKASSILLDNGANTVVAIVTHGIL  255
ADH7                    -KRE--DSMKLGADHYIAMLEDKGWTEQYSNALDLLVVCSSSLSKVNFDSIVKIMK----  268
KA_contig_17.g1558      -KKE--DALKLGADHYVATLEDEDWDKKLFDTFDLVVVCASSLTDIQIDRFVKVMK----  269
KS_contig_10.g24        -KKE--DALKLGADHYVATMEDEDWDKKLFDTFDLVVVCASSLTDIKIDRFVKVMK----  269
ADH6                    -KRE--DAMKMGADHYIATLEEGDWGEKYFDTFDLIVVCASSLTDIDFNIMPKAMK----  267
KS_contig_24.g2365      -KKE--DAMKLGADHFIATKEEPTWAEDYFDTLDLVVICAGSLTDVDFTKLPKIMK----  267
KA_contig_21.g2255      -KKE--DAMKLGADHFIATKEESTWAEDYFDTLDLVVICAGSLTDVDFTKLPKIMK----  267
KA_contig_19.g1873      -KKD--DAMKLGADHFIATKEQPTWAEDNFDTLDLVVICAGSLTDVDFTKLPKIMK----  267
KA_contig_3.g2902       -KKD--DAMKLGADHFIATKEQPTWAEDNFDTLDLVVICAGSLTDVDFTKLPKIMK----  267
                         * :  . : : .*      :     ..  :: . :* .:. * .     .    :.

KS_contig_25.g2372      SGDAIKNINGSKLDKVVCTN----------TVQFGNKL--EQCSK----------LEIID  293
ADH7                    IGGSIVSIAAPEVNEKLVLKPLGLMGVSISSSAIGSRKEIEQLLKLVSEKNVKIWVEKLP  328
KA_contig_17.g1558      IGSSIISITAPSREEKLVLNPLGLLGVKIGNSGIGSVKEIKTMLDLVAKKNLKIWVETIP  329
KS_contig_10.g24        IGSSIISITAPSREEKLVLNPLGLLGVKIGNSGIGSVKEIKTMLDLVAKKNLKIWVETIP  329
ADH6                    VGGRIVSISIPEQHEMLSLKPYGLKAVSISYSALGSIKELNQLLKLVSEKDIKIWVETLP  327
KS_contig_24.g2365      VGGAIRSISIPEASQKLTLQPFGLVGVSIGNSALGSIKETKQLLKLVSEKDLKIWVETIP  327
KA_contig_21.g2255      IGGAIRSISVPDQSQKLTLSPFGLVGVSIGNSALGSIKEVKQLLKLVSEKDLKIWVETIP  327
KA_contig_19.g1873      VGGAIRSISIPDQSQKLTLSPFGLVGVSIGNSMLGSIKETKQLLQLVSEKDLKIWVETIP  327
KA_contig_3.g2902       VGGAIRSISIPDQSQKLTLSPFGLVGVSIGNSALGSIKETKQLLQLVSEKNLKIWVETIP  327
                         *. * .*    .  : :   .                :*.     :    .         :* :

KS_contig_25.g2372      ISN-VLAESIRRLHNGESVSYLFRNH---------        318
ADH7                    ISEEGVSHAFTRMESGD-VKYRFTLVDYDKKFHK*        361
KA_contig_17.g1558      ISEKGVNEAFERMESGD-VRYRFTLVDYDKEFN--        361
KS_contig_10.g24        ISEKGVNEAFERMESGD-VRYRFTLVDYDKEFK--        361
ADH6                    VGEAGVHEAFERMEKGD-VRYRFTLVGYDKEFSD*        360
KS_contig_24.g2365      ISEKGVQEAFERMDKGD-VRYRFSLTDFDKEFGN-        360
KA_contig_21.g2255      ISEKGVQEAFERMDKGD-VRYRFTLTDFDKEFDN-        360
```

```
KA_contig_19.g1873      ISEKGVQEAFERMDKGD-VRYRFSLTDFDKEFGN-        360
KA_contig_3.g2902       ISEKGVQEAFERMDKGD-VRYRFSLTDFDKEFGN-        360
                        :.:  : .:: *:..*: * * *
```

Supplementary Figure 14b.

```
KS_contig_25.g2372        MATN------SIKLIGSDGQLNI-------VKSIAKRLSIPITECVLIRDPNQEIVFSIS  47
CAY80561.2                --MLYPEKFQGIGISNAKDWKHPKLVSFDPKPFGDHDVDVEIEACGICGSDFHIAVGNWG  58
KA_contig_17.g1558        MTTNYPEEFQGFGIVDKDDWLHPKKINFKPKEFTPNSVDIKIECCGVCGSDIHCAKSDWS  60
KS_contig_10.g24          MTTNYPEEFQGFGIVDKDDWLHPKKINFKPKEFTPNSVDIKIECCGVCGSDIHCAKSDWS  60
CAY82157.1                --MSYPEKFEGIAIQSHEDWKNPKKTKYDPKPFYDHDIDIKIEACGVCGSDIHCAAGHWG  58
KS_contig_24.g2365        --MSYPEKFQGIAVLDHADYTHPKKVAFDPKPFHPSDIDIAIEACGVCGSDCHAANGGWG  58
KA_contig_21.g2255        --MSYPEKFQGIAVLDHADYTHPKKVSYDPKPFSPTDIDISIEACGVCGSDCHSANGGWG  58
KA_contig_19.g1873        --MSYPEKFQGIAVLDHADYTHPKKVSFDPKPFAPSDIDISIEACGVCGSDCHAANGGWG  58
KA_contig_3.g2902         --MSYPEKFQGIAVLDHADYTHPKKVSFDPKPFAPSDIDISIEACGVCGSDCHAANGGWG  58
                                    .: : .  .  :               :.: *  * :  .  :      .

KS_contig_25.g2372        E-----TVRDQDVYIVAQIGSD-----KVNDR------VFELLIMINAAKAASARKITVV  91
CAY80561.2                PVPENQILGHEIIGRVVKVGSKCHTGVKIGDRVGVGAQALACFE---CER--------CK  107
KA_contig_17.g1558        ELAHGQIVGHEIIGTVTKIGPGCKSGLKLGDRVGVGAQVFSCLD---CDR--------CK  109
KS_contig_10.g24          ELSHGQVVGHEIIGTVAKIGPDCNTGLKIGDRVGVGAQVFSCLD---CDR--------CK  109
CAY82157.1                NMKMPLVVGHEIVGKVVKLGPKSNSGLKVGQRVGVGAQVFSCLE---CDR--------CK  107
KS_contig_24.g2365        DKPKPLVVGHEVIGRVVKIGPNCTTGLKLGDRVGVGAQVLSCLE---CNR--------CK  107
KA_contig_21.g2255        DKPKPLVVGHEVIGRVVKMGPNCTSGLKIGDRVGVGAQVLSCLE---CDR--------CK  107
KA_contig_19.g1873        DKPKPLVVGHEVIGRVVKMGPNCTSGLKIGDRVGVGAQVLSCLE---CDR--------CK  107
KA_contig_3.g2902         DKPKPLVVGHEVIGRVVKMGPNCTSGLKIGDRVGVGAQVLSCLE---CDR--------CK  107
                                 : .: :  *.::*       *:.:*      .:  :    . :

KS_contig_25.g2372        IPNFPYARQDKKDKARAPITAKLMADMLTTAGCDHIVTMDLHAAQIQGFFDVPLDNLYAE  151
CAY80561.2                SDNEQYCTNDHVLTMWTPYK----DGYISQGGFASHV-------RLHEHFAIQIPENIPS  156
KA_contig_17.g1558        NNNEPYCPK-FVTTYSQPYE----DGYISQGGYANYV-------RVHEHFAIPIPEEIPS  157
KS_contig_10.g24          NDNEPYCPK-FVTTYSQPYE----DGYISQGGYANYI-------RVHEHFAVPIPEEIPS  157
CAY82157.1                NDNEPYCTK-FVTTYSQPYE----DGYVSQGGYANYV-------RVHEHFVVPIPENIPS  155
KS_contig_24.g2365        EDNEPYCPK-FVTTYGQDYE----DGYNSKGGYASHI-------RVHEHFVVPIPENIPS  155
KA_contig_21.g2255        ADNEPYCPK-FVTTYGQDYE----DGYNSKGGYASHI-------RVHEHFVVPIPENIPS  155
KA_contig_19.g1873        ADNEPYCPR-FVTTYGQDYE----DGYNSKGGYASHI-------RVHEHFVVPIPENIPS  155
KA_contig_3.g2902         ADNEPYCPK-FVTTYGQDYE----DGYNSKGGYASHI-------RVHEHFVVPIPEYIPS  155
                            *  *. .    .           .  : .*    :       ::: .* : : :   .

KS_contig_25.g2372        PSIVNYIKEHLDLNNCIIVSPDAGGTKRVASLA------------EDLNL-TFALIHKER  198
CAY80561.2                PLAAPLLCGGITVFS-PLLRNGCGPGKRVGIVGIGGIGHMGILLAKAMGAEVYAFSRGHS  215
KA_contig_17.g1558        ELAAPLMCGGITVFS-PLLRNGCGPGKKVGIVGIGGIGHMGVIFAKALGAEVWAFSRKNN  216
KS_contig_10.g24          ELAAPLMCGGITVFS-PLLRNGCGPGKKVGIVGIGGIGHMGVIFAKALGAEVWAFSRKNN  216
CAY82157.1                HLAAPLLCGGLTVYS-PLVRNGCGPGKKVGIVGLGGIGSMGTLISKAMGAETYVISRSSR  214
KS_contig_24.g2365        ELAAPLLCGGLTVFS-PLVRNGCGPGKKVGILGIGGIGHMGVIFAKALGAEVYAISRSSA  214
KA_contig_21.g2255        HLAAPLLCGGLTVFS-PLVRNGCGPGKKVGVLGIGGIGHMGVIFAKALGAEVYAISRTSA  214
KA_contig_19.g1873        HLAAPLLCGGLTVFS-PLVRNGCGPGKKVGILGIGGIGHMGVLFAKALGAEVYAISRTSA  214
KA_contig_3.g2902         HLAAPLLCGGLTVFS-PLVRNGCGPGKKVGILGIGGIGHMGVIFAKALGAEVYAISRTSA  214
                             .  :   : : .  ::  ..*  *:*. :.            : :.  .:.: :

KS_contig_25.g2372        VKANEVSRMVLVGDVTG---KTCIIVDDMADTCGTLVKASSILLDNGANTVVAIVTHGIL  255
CAY80561.2                -KRE--DSMKLGADHYIAMLEDKGWTEQYSNALDLLVVCSSSLSKVNFDSIVKIMK----  268
KA_contig_17.g1558        -KKE--DALKLGADHYVATLEDEDWDKKLFDTFDLVVVCASSLTDIQIDRFVKVMK----  269
KS_contig_10.g24          -KKE--DALKLGADHYVATMEDEDWDKKLFDTFDLVVVCASSLTDIKIDRFVKVMK----  269
CAY82157.1                -KRE--DAMKMGADHYIATLEEGDWGEKYFDTFDLIVVCASSLTDIDFNIMPKAMK----  267
KS_contig_24.g2365        -KKE--DAMKLGADHFIATKEEPTWAEDYFDTLDLVVICAGSLTDVDFTKLPKIMK----  267
KA_contig_21.g2255        -KKE--DAMKLGADHFIATKEESTWAEDYFDTLDLVVICAGSLTDVDFTKLPKIMK----  267
KA_contig_19.g1873        -KKD--DAMKLGADHFIATKEQPTWAEDNFDTLDLVVICAGSLTDVDFTKLPKIMK----  267
KA_contig_3.g2902         -KKD--DAMKLGADHFIATKEQPTWAEDNFDTLDLVVICAGSLTDVDFTKLPKIMK----  267
                           * :  . : : .*      :     ..  :: . :* .:. * .     .   :.

KS_contig_25.g2372        SGDAIKNINGSKLDKVVCTN----------TVQFGNKL--EQCSK----------LEIID  293
CAY80561.2                IGGSIVSIAAPEVNEKLVLKPLGLMGVSISSSAIGSRKEIEQLLKLVSEKNVKIWVEKLP  328
KA_contig_17.g1558        IGSSIISITAPSREEKLVLNPLGLLGVKIGNSGIGSVKEIKTMLDLVAKKNLKIWVETIP  329
KS_contig_10.g24          IGSSIISITAPSREEKLVLNPLGLLGVKIGNSGIGSVKEIKTMLDLVAKKNLKIWVETIP  329
CAY82157.1                VGGRIVSISIPEQHEMLSLKPYGLKAVSISYSALGSIKELNQLLKLVSEKDIKIWVETLP  327
KS_contig_24.g2365        VGGAIRSISIPEASQKLTLQPFGLVGVSIGNSALGSIKETKQLLKLVSEKDLKIWVETIP  327
KA_contig_21.g2255        IGGAIRSISVPDQSQKLTLSPFGLVGVSIGNSALGSIKEVKQLLKLVSEKDLKIWVETIP  327
KA_contig_19.g1873        VGGAIRSISIPDQSQKLTLSPFGLVGVSIGNSMLGSIKETKQLLQLVSEKDLKIWVETIP  327
KA_contig_3.g2902         VGGAIRSISIPDQSQKLTLSPFGLVGVSIGNSALGSIKETKQLLQLVSEKNLKIWVETIP  327
                           *. * .*   .  : :  .             :*.    :   .          :* :

KS_contig_25.g2372        ISN-VLAESIRRLHNGESVSYLFRNH--------        318
CAY80561.2                ISEEGVSHAFTRMESGD-VKYRFTLVDYDKKFHK        361
KA_contig_17.g1558        ISEKGVNEAFERMESGD-VRYRFTLVDYDKEFN-        361
KS_contig_10.g24          ISEKGVNEAFERMESGD-VRYRFTLVDYDKEFK-        361
CAY82157.1                VGEAGGHEAFERMEKGD-VRYRFTLVGYDKEFSD        360
KS_contig_24.g2365        ISEKGVQEAFERMDKGD-VRYRFSLTDFDKEFGN        360
KA_contig_21.g2255        ISEKGVQEAFERMDKGD-VRYRFTLTDFDKEFDN        360
KA_contig_19.g1873        ISEKGVQEAFERMDKGD-VRYRFSLTDFDKEFGN        360
```

```
KA_contig_3.g2902       ISEKGVQEAFERMDKGD-VRYRFSLTDFDKEFGN        360
                        :.:    .:: *:..*: * * *
```

Supplementary Figure 15a.

```
ALD6                    MTKLHFDTAEPVKITLPNGLTYEQPTGLFINNKFMKAQDGKTYPVEDPSTENTVCEVSSA     60
KA_scaffold_15.g5208    -MKYNFEQSDPVEITLPNGLTYSQPTGLFINNEFIQSQSYATSTIEDPATETPIVDVASG     59
KS_scaffold_20.g5255    -MKYNFEQSDPVEITLPNGLTYSQPTGLFINNEFIQSQSYATSTIEDPATETPIVDVASG     59
KA_scaffold_15.g5207    -MKYNFEIADPVEVTLPNGVKYSQPTGLFINNQFIQSHNQKTIEVENPATQETIVNVSSG     59
KS_scaffold_20.g5254    -MKYNFEIAEPVEVTLPNGVKYSQPTGLFINNQFIQSHNHKTIDVENPATQETIVAVSTG     59
                          * :*: ::**::*****:.*.*********:*::::.  *  :*:*:*:  :  *::.

ALD6                    TTEDVEYAIECADRAFHDTEWATQDPRERGRLLSKLADELESQIDLVSSIEALDNGKTLA     120
KA_scaffold_15.g5208    ASEDVDYAVECAEYTFNNSKWATQDPKLRAKVLFKLADLVEQNKELIASIETYNNGKTLA     119
KS_scaffold_20.g5255    SSEDVDYAVECAEYTFNQSKWATQDPKLRAKVIYKLADLVEQNKELIASIETYNNGKTLA     119
KA_scaffold_15.g5207    TVEDVEYAVASAEKAFKDTNWAKQDPKVRAKALFKLADLVEENLDLFTAVETTNNGKTLA     119
KS_scaffold_20.g5254    TVEDVEYAVESAERAFKDTNWATQDPKVRAKALFKLADLVEENIDLFSAVETANNGKTLA     119
                        : ***:**: .*: :*::::**.***: *.: : ****  :*.: :*.:::*: :******

ALD6                    LARGDVTIAINCLRDAAAYADKVNGRTINTGDGYMNFTTLEPIGVCGQIIPWNFPIMMLA     180
KA_scaffold_15.g5208    LATGDVEVGIDCLRDAAAYADKINGRVIESGDEYMNFTMKTPIGVCGQIIPWNFPFMMLI     179
KS_scaffold_20.g5255    LSTGDVEVGIDCLRDAASYADKINGRVIESGEEYMNFTMKTPIGVCGQIIPWNFPFMMLI     179
KA_scaffold_15.g5207    LSAGDVQIGIDCLRDAASYADKINGRTMDTGDGYMNFTMKSPIGICGQIIPWNFPFMMLI     179
KS_scaffold_20.g5254    LSAGDVQIGIDCLRDAASYADKINGRTMDTGDGYMNFTMKSPIGICGQIIPWNFPFMMLI     179
                        *: *** :.*:******:****:***.:::*: *****   ***:**********:***

ALD6                    WKIAPALAMGNVCILKPAAVTPLNALYFASLCKKVGIPAGVVNIVPGPGRTVGAALTNDP     240
KA_scaffold_15.g5208    WKIAPAIAMGNTIILKPAPATPLSALFFASLVQQSGMPAGVVNIIPSAGVSVGTALTSHP     239
KS_scaffold_20.g5255    WKIAPAIAMGNTIILKPAPATPLTALFFASLVQQAGMPAGVVNIIPSAGVSVGTALTQHP     239
KA_scaffold_15.g5207    WKIAPALAMGNVLILKPASATPLTALLLCSFMEKAGMPAGVVNIVPGSGREVGTAITEHP     239
KS_scaffold_20.g5254    WKIAPALAMGNVSILKPASATPLTALLLCSFMEKAGFPAGVVNIVPGSGREVGTAITEHP     239
                        ******:****. *****  .***.**  :.*: :: *:*******:*. *  **:*:*..*

ALD6                    RIRKLAFTGSTEVGKSVAVDSSESNLKKITLELGGKSAHLVFDDANIKKTLPNLVNGIFK     300
KA_scaffold_15.g5208    KIRKIAFTGSTGIGKHVANSAASSNLKKTTLELGGKSAHLVFDDANIEKTLPNLVNGIFK     299
KS_scaffold_20.g5255    KIRKIAFTGSTGIGKHVANSAATSNLKKTTLELGGKSAHLVFNDANIEKTLPNLVNGIFK     299
KA_scaffold_15.g5207    KIRKIAFTGSTEIGKDIAVRSSAANLKKATLELGGKSAHLVFDDANIEKTLPNLVNGIFK     299
KS_scaffold_20.g5254    KIRKIAFTGSTDIGKDIAVRSSAANLKKATLELGGKSAHLVFNDANIEKTLPNLVNGIFK     299
                        :***:****** :** :*   :: :****  ************:****:***********

ALD6                    NAGQICSSGSRIYVQEGIYDELLAAFKAYLETEIKVGNPFDKANFQGAITNRQQFDTIMN     360
KA_scaffold_15.g5208    NAGQICSSGSRIYIQEGIYDELLAAFKTYVE-NLKVGNPFDKSNFQGAISTKPQFETIMN     358
KS_scaffold_20.g5255    NAGQICSSGSRIYIQEGIYDELLAAFKTYVE-DLKVGNPFDKSNFQGAISTKPQFETILN     358
KA_scaffold_15.g5207    NAGQICSSGSRIYIQEGIYDELLAAFKTYVE-NLKVGDPFDKSNFQGAITNKGQFDTIMK     358
KS_scaffold_20.g5254    NAGQICSSGSRIYIQEGIYDELLAAFKTYVE-DLKVGDPFDKSNFQGAITNKGQFDTIMK     358
                        *************:*************:*:*  ::***:****:******:.: **:**::

ALD6                    YIDIGKKEGAKILTGGEKVGDKGYFIRPTVFYDVNEDMRIVKEEIFGPVVTVAKFKTLEE     420
KA_scaffold_15.g5208    YINIGKEEGAKILTGGERVGDKGYFIRPTIFYDVKEDMRIVKEEIFGPVVTISKFTTIED     418
KS_scaffold_20.g5255    YINIGKEEGAKILTGGERVGDKGYFVRPTIFYDVKEDMRIVKEEIFGPVVTISKFSTIED     418
KA_scaffold_15.g5207    YIKIGKEEGAKILTGGERVGDKGYFIRPTIFYDVKEDMRIVKEEIFGPVVTISKFTTIED     418
KS_scaffold_20.g5254    YINIGKEEGAKILTGGERVGDKGYFVRPTIFYDVKEDMKIVKEEIFGPVVTISKFSTIED     418
                        **.***:**********:*******:***:****:***:***********::**.*:*:

ALD6                    GVEMANSSEFGLGSGIETESLSTGLKVAKMLKAGTVWINTYNDFDSRVPFGGVKQSGYGR     480
KA_scaffold_15.g5208    GVAKANDSEFGLGAGIETENLSTALKVAKMLHAGTIWVNTYNDFDSRVPFGGVKQSGYGR     478
KS_scaffold_20.g5255    GVAMANDSEFGLGAGIETENLSTALRVAKMLHAGTVWVNTYNDFDSRVPFGGVKQSGYGR     478
KA_scaffold_15.g5207    GVAMANDSEFGLGAGIETENLSTGLRVARMLHSGTVWINTYNDFDSRVPFGGVKQSGYGR     478
KS_scaffold_20.g5254    GVAMANDSEFGLGAGIETENISTGLRVARMLHSGTVWINTYNDFDSRVPFGGVKQSGYGR     478
                        **  **.******:*****.:**.*:**:**::**:*:*********************

ALD6                    EMGEEVYHAYTEVKAVRIKL*    500
KA_scaffold_15.g5208    EMGEEVYANYTEVKAVRIKL-    498
KS_scaffold_20.g5255    EMGEEVYANYTEVKAVRIKL-    498
KA_scaffold_15.g5207    EMGTEVYDNYTEVKAVRIKL-    498
KS_scaffold_20.g5254    EMGTEVYDNYTEVKAVRIKL-    498
                        *** ***  **********
```

Supplementary Figure 15b.

```
CAY86900.1              MTKLHFDTAEPVKITLPNGLTYEQPTGLFINNKFMKAQDGKTYPVEDPSTENTVCEVSSA     60
KA_scaffold_15.g5208    -MKYNFEQSDPVEITLPNGLTYSQPTGLFINNEFIQSQSYATSTIEDPATETPIVDVASG     59
KS_scaffold_20.g5255    -MKYNFEQSDPVEITLPNGLTYSQPTGLFINNEFIQSQSYATSTIEDPATETPIVDVASG     59
KA_scaffold_15.g5207    -MKYNFEIADPVEVTLPNGVKYSQPTGLFINNQFIQSHNQKTIEVENPATQETIVNVSSG     59
KS_scaffold_20.g5254    -MKYNFEIAEPVEVTLPNGVKYSQPTGLFINNQFIQSHNHKTIDVENPATQETIVAVSTG     59
                          * :*: ::**::*****:.*.*********:*::::.  *  :*:*:*:  :  *::.

CAY86900.1              TTEDVEYAIECADRAFHDTEWATQDPRERGRLLSKLADELESQIDLVSSIEALDNGKTLA     120
KA_scaffold_15.g5208    ASEDVDYAVECAEYTFNNSKWATQDPKLRAKVLFKLADLVEQNKELIASIETYNNGKTLA     119
KS_scaffold_20.g5255    SSEDVDYAVECAEYTFNQSKWATQDPKLRAKVIYKLADLVEQNKELIASIETYNNGKTLA     119
KA_scaffold_15.g5207    TVEDVEYAVASAEKAFKDTNWAKQDPKVRAKALFKLADLVEENLDLFTAVETTNNGKTLA     119
KS_scaffold_20.g5254    TVEDVEYAVESAERAFKDTNWATQDPKVRAKALFKLADLVEENIDLFSAVETANNGKTLA     119
                        : ***:**: .*: :*::::**.***: *.: : **** :*.: :*.:::*: :******

CAY86900.1              LARGDVTIAINCLRDAAAYADKVNGRTINTGDGYMNFTTLEPIGVCGQIIPWNFPIMMLA     180
KA_scaffold_15.g5208    LATGDVEVGIDCLRDAAAYADKINGRVIESGDEYMNFTMKTPIGVCGQIIPWNFPFMMLI     179
KS_scaffold_20.g5255    LSTGDVEVGIDCLRDAASYADKINGRVIESGEEYMNFTMKTPIGVCGQIIPWNFPFMMLI     179
KA_scaffold_15.g5207    LSAGDVQIGIDCLRDAASYADKINGRTMDTGDGYMNFTMKSPIGICGQIIPWNFPFMMLI     179
KS_scaffold_20.g5254    LSAGDVQIGIDCLRDAASYADKINGRTMDTGDGYMNFTMKSPIGICGQIIPWNFPFMMLI     179
                        *: *** :.*:******:****:***.:::*: *****   ***:*********:***

CAY86900.1              WKIAPALAMGNVCILKPAAVTPLNALYFASLCKKVGIPAGVVNIVPGPGRTVGAALTNDP     240
KA_scaffold_15.g5208    WKIAPAIAMGNTIILKPAPATPLSALFFASLVQQSGMPAGVVNIIPSAGVSVGTALTSHP     239
KS_scaffold_20.g5255    WKIAPAIAMGNTIILKPAPATPLTALFFASLVQQAGMPAGVVNIIPSAGVSVGTALTQHP     239
KA_scaffold_15.g5207    WKIAPALAMGNVLILKPASATPLTALLLCSFMEKAGMPAGVVNIVPGSGREVGTAITEHP     239
KS_scaffold_20.g5254    WKIAPALAMGNVSILKPASATPLTALLLCSFMEKAGFPAGVVNIVPGSGREVGTAITEHP     239
                        ******:****. ***** .***.** :.*: :: *:*******:*. *  **:*:*..*

CAY86900.1              RIRKLAFTGSTEVGKSVAVDSSESNLKKITLELGGKSAHLVFDDANIKKTLPNLVNGIFK     300
KA_scaffold_15.g5208    KIRKIAFTGSTGIGKHVANSAASSNLKKTTLELGGKSAHLVFDDANIEKTLPNLVNGIFK     299
KS_scaffold_20.g5255    KIRKIAFTGSTGIGKHVANSAATSNLKKTTLELGGKSAHLVFNDANIEKTLPNLVNGIFK     299
KA_scaffold_15.g5207    KIRKIAFTGSTEIGKDIAVRSSAANLKKATLELGGKSAHLVFDDANIEKTLPNLVNGIFK     299
KS_scaffold_20.g5254    KIRKIAFTGSTDIGKDIAVRSSAANLKKATLELGGKSAHLVFNDANIEKTLPNLVNGIFK     299
                        :***:****** :** :*  :: :**** *************:****:***********

CAY86900.1              NAGQICSSGSRIYVQEGIYDELLAAFKAYLETEIKVGNPFDKANFQGAITNRQQFDTIMN     360
KA_scaffold_15.g5208    NAGQICSSGSRIYIQEGIYDELLAAFKTYVE-NLKVGNPFDKSNFQGAISTKPQFETIMN     358
KS_scaffold_20.g5255    NAGQICSSGSRIYIQEGIYDELLAAFKTYVE-DLKVGNPFDKSNFQGAISTKPQFETILN     358
KA_scaffold_15.g5207    NAGQICSSGSRIYIQEGIYDELLAAFKTYVE-NLKVGDPFDKSNFQGAITNKGQFDTIMK     358
KS_scaffold_20.g5254    NAGQICSSGSRIYIQEGIYDELLAAFKTYVE-DLKVGDPFDKSNFQGAITNKGQFDTIMK     358
                        *************:*************:*:* ::***:****:******:.: **:**::

CAY86900.1              YIDIGKKEGAKILTGGEKVGDKGYFIRPTVFYDVNEDMRIVKEEIFGPVVTVAKFKTLEE     420
KA_scaffold_15.g5208    YINIGKEEGAKILTGGERVGDKGYFIRPTIFYDVKEDMRIVKEEIFGPVVTISKFTTIED     418
KS_scaffold_20.g5255    YINIGKEEGAKILTGGERVGDKGYFVRPTIFYDVKEDMRIVKEEIFGPVVTISKFSTIED     418
KA_scaffold_15.g5207    YIKIGKEEGAKILTGGERVGDKGYFIRPTIFYDVKEDMRIVKEEIFGPVVTISKFTTIED     418
KS_scaffold_20.g5254    YINIGKEEGAKILTGGERVGDKGYFVRPTIFYDVKEDMKIVKEEIFGPVVTISKFSTIED     418
                        **.***:**********:*******:***:****:***:***********::**.*:*:

CAY86900.1              GVEMANSSEFGLGSGIETESLSTGLKVAKMLKAGTVWINTYNDFDSRVPFGGVKQSGYGR     480
KA_scaffold_15.g5208    GVAKANDSEFGLGAGIETENLSTALKVAKMLHAGTIWVNTYNDFDSRVPFGGVKQSGYGR     478
KS_scaffold_20.g5255    GVAMANDSEFGLGAGIETENLSTALRVAKMLHAGTVWVNTYNDFDSRVPFGGVKQSGYGR     478
KA_scaffold_15.g5207    GVAMANDSEFGLGAGIETENLSTGLRVARMLHSGTVWINTYNDFDSRVPFGGVKQSGYGR     478
KS_scaffold_20.g5254    GVAMANDSEFGLGAGIETENISTGLRVARMLHSGTVWINTYNDFDSRVPFGGVKQSGYGR     478
                        **  **.******:*****.:**.*:**:**::**:*:*********************

CAY86900.1              EMGEEVYHAYTEVKAVRIKL     500
KA_scaffold_15.g5208    EMGEEVYANYTEVKAVRIKL     498
KS_scaffold_20.g5255    EMGEEVYANYTEVKAVRIKL     498
KA_scaffold_15.g5207    EMGTEVYDNYTEVKAVRIKL     498
KS_scaffold_20.g5254    EMGTEVYDNYTEVKAVRIKL     498
                        *** ***  ***********
```

Supplementary Figure 16a.

```
BDH1                      MRALAYFKKGDIHFTNDIPRPEIQTDDEVIIDVSWCGICGSDLHEYLDGPIFMPKDGECH  60
KA_contig_13.g329         MRALAYFKEGDIHFTDSLPEPKLTTPDSMLITPSFVGLCGSDLHEA-EHPIFYPQDGHTH  59
KA_contig_30.g3329        MRALAYFKEGDIHFTDSLPEPKLSTPDSMLITPSFVGLCGSDLHEA-EHPIFYPQDGHTH  59
KA_contig_25.g2491        MRALAYFKEGDIHFTDSLPEPKLTSPDSLLISPSFVGLCGSDLHEA-EHPIFFPKDGHRH  59
KS_contig_6.g4029         MRGLAYFKEGDIHFTDSLPEPKLTSPDSLLISPSFVGLCGSDLHEA-SHPIFFPKDGHRH  59
                          **.*****:******:.:*.*:: : *.::*  *: *:*******  . *** *:**. *

BDH1                      KLSNAALPLAMGHEMSGIVSKVGPKVTKVKVGDHVVVDAASSCADLHCWPHSKFYNSKPC  120
KA_contig_13.g329         KLSGIPLPQALGHEISGYVKAVGPKVTKFKAGDRVVIEATTSCLDKHRWPTAKNANTPTC  119
KA_contig_30.g3329        KLSGIPLPQALGHEISGYVKAVGPKVTKFKAGDRVVIEATTSCLDKHRWPTAKNANTPTC  119
KA_contig_25.g2491        KLSENELPQALGHEVSGYVKAIGPKVTKFKVGDRVVVEASASCVDKHRWPNAKFANTPTC  119
KS_contig_6.g4029         KLSENELPQALGHEVSGYVKAIGPAVTQFKVGDRVVVEASASCVDKHRWPNAKFANTPIC  119
                          ***   ** *:***:** *. :** **:.*.**:**::*::** * * ** :*  *:  *

BDH1                      DACQRGSENLCTHAGFVGLGVISGGFAEQVVVSQHHIIPVPKEIPLDVAALVEPLSVTWH  180
KA_contig_13.g329         DACKAGRTNCCEYAGFSGLGVVSGGFAETMDTIEHHVVKLPDFVPMDVGALVEPLSVAWH  179
KA_contig_30.g3329        DACKAGRTNCCEYAGFSGLGVVSGGFAETMDTIEHHVVKLPDFVPMEVGALVEPLSVAWH  179
KA_contig_25.g2491        DACSEGYENCCEYAGFSGLGVVSGGFADTMETIEHHVVKLPDFLPMDVGALVEPLSVAWH  179
KS_contig_6.g4029         DACNEGYENCCEYAGFSGLGVVSGGFADTMETIEHHVVKLPDFLPMDVGALVEPLSVAWH  179
                          ***. *  * * :*** ****:*****: : . :**:: :*. :*::*.********:**

BDH1                      AVKISGFKKGSSALVLGAGPIGLCTILVLKGMGASKIVVSEIAERRIEMAKKLGVEVFNP  240
KA_contig_13.g329         GATVAKFTPGKTALILGSGPIGLAMILVLKAKGAKKIVVSEPASIRRELAAKLNVETFDP  239
KA_contig_30.g3329        GATVAKFTPGKTALILGSGPIGLAMILVLKAKGAKKIVVSEPASIRRELAAKLNVETFDP  239
KA_contig_25.g2491        GSRVAKFTPGKTALVLGAGPIGLAMILVLKAKGAKKIVVSELASIRRELAEKFNVETFDS  239
KS_contig_6.g4029         GSRVANFTPGKTALILGAGPIGLAMILVLKAKGAKKIVVSELASIRRELAERFNVETFDP  239
                          .  :: *. *.:**:**:*****. *****. **.****** *. * *:* ::.**.*:

BDH1                      SKHGHKSIEILRGLTKSHDGFDYSYDCSGIQVTFETSLKALTFKGTATNIAVWGPKPVPF  300
KA_contig_13.g329         TKYKNDAVSVLRSIPEGNKGFDFAFDCSGVPSTLNTGVGAIHFRGIHCNVAIWGK-GLDF  298
KA_contig_30.g3329        TKYKNDAVSVLRSIPEGNEGFDFAFDCSGVPSTLNTGVGAIHFRGIHCNVAIWGK-GLDF  298
KA_contig_25.g2491        SKHGDNAISELRAITEGNKGFDFAFDCSGVPPTFNTGIGAIHFRGVYCNVAIWGK-GLNF  298
KS_contig_6.g4029         SKHGDNAISELRSITEGNKGFDFAFDCSGVAPTFNTGIAAIHFRGTYCNVAIWGK-GLNF  298
                          :*: ..::. **.: :.:.***::****:  *::*.: *: *:*   *:*:**   : *

BDH1                      QPMDVTLQEKVMTGSIGYVVEDFEEVVRAIHNGDIAMEDCKQLITGKQRIEDGWEKGFQE  360
KA_contig_13.g329         NPMDITFQEKVFVGSIGYTIEDFQQVIDAFERKKIDPKECENLITGRQKIEDGWEMGFLE  358
KA_contig_30.g3329        NPMDITFQEKVFVGSIGYTIEDFQQVIDAFERKKIDPKECENLITGRQKIEDGWEKGFLE  358
KA_contig_25.g2491        NPMDITLQEKNLTGSIGYTVEDFKQVVDAFENKKIDPKECEHLITGRQKIEDGWEKGFLE  358
KS_contig_6.g4029         NPMDITLQEKNLTGSIGYTVEDFKQVVDAFEKKKIDPKECEHLITGRQKIEDGWEKGFLE  358
                          :***:*:*** :.*****.:***::*: *:.. .*  ::*::****:*:****** ** *

BDH1                      LMDHKESNVKILLTPNNHGEMK* 382
KA_contig_13.g329         LINHKDTNIKVLLTPNTHGELDR 381
KA_contig_30.g3329        LINHKDTNIKVLLTPNTHGELDR 381
KA_contig_25.g2491        LMNHKDTNIKVLLTPNNHGELDG 381
KS_contig_6.g4029         LMNHKDTNIKVLLTPNNHGELDG 381
                          *::**::*:*:*****.***:.
```

Supplementary Figure 16b.

```
CAY77584.1             MRALAYFKKGDIHFTNDIPRPEIQTDDEVIIDVSWCGICGSDLHEYLDGPIFMPKDGECH  60
KA_contig_13.g329      MRALAYFKEGDIHFTDSLPEPKLTTPDSMLITPSFVGLCGSDLHEA-EHPIFYPQDGHTH  59
KA_contig_30.g3329     MRALAYFKEGDIHFTDSLPEPKLSTPDSMLITPSFVGLCGSDLHEA-EHPIFYPQDGHTH  59
KA_contig_25.g2491     MRALAYFKEGDIHFTDSLPEPKLTSPDSLLISPSFVGLCGSDLHEA-EHPIFFPKDGHRH  59
KS_contig_6.g4029      MRGLAYFKEGDIHFTDSLPEPKLTSPDSLLISPSFVGLCGSDLHEA-SHPIFFPKDGHRH  59
                       **.*****:******:.:*.*:: : *.::*  *: *:*******  . *** *:**. *

CAY77584.1             KLSNAALPLAMGHEMSGIVSKVGPKVTKVKVGDHVVVDAASSCADLHCWPHSKFYNSKPC  120
KA_contig_13.g329      KLSGIPLPQALGHEISGYVKAVGPKVTKFKAGDRVVIEATTSCLDKHRWPTAKNANTPTC  119
KA_contig_30.g3329     KLSGIPLPQALGHEISGYVKAVGPKVTKFKAGDRVVIEATTSCLDKHRWPTAKNANTPTC  119
KA_contig_25.g2491     KLSENELPQALGHEVSGYVKAIGPKVTKFKVGDRVVVEASASCVDKHRWPNAKFANTPTC  119
KS_contig_6.g4029      KLSENELPQALGHEVSGYVKAIGPAVTQFKVGDRVVVEASASCVDKHRWPNAKFANTPIC  119
                       ***   ** *:***:** *. :** **:.*.**:**::*::** * * ** :*  *:  *

CAY77584.1             DACQRGSENLCTHAGFVGLGVISGGFAEQVVVSQHHIIPVPKEIPLDVAALVEPLSVTWH  180
KA_contig_13.g329      DACKAGRTNCCEYAGFSGLGVVSGGFAETMDTIEHHVVKLPDFVPMDVGALVEPLSVAWH  179
KA_contig_30.g3329     DACKAGRTNCCEYAGFSGLGVVSGGFAETMDTIEHHVVKLPDFVPMEVGALVEPLSVAWH  179
KA_contig_25.g2491     DACSEGYENCCEYAGFSGLGVVSGGFADTMETIEHHVVKLPDFLPMDVGALVEPLSVAWH  179
KS_contig_6.g4029      DACNEGYENCCEYAGFSGLGVVSGGFADTMETIEHHVVKLPDFLPMDVGALVEPLSVAWH  179
                       ***. *  * * :*** ****:*****: : . :**:: :*. :*::*.********:**

CAY77584.1             AVKISGFKKGSSALVLGAGPIGLCTILVLKGMGASKIVVSEVAERRIEMAKKLGVEVFNP  240
KA_contig_13.g329      GATVAKFTPGKTALILGSGPIGLAMILVLKAKGAKKIVVSEPASIRRELAAKLNVETFDP  239
KA_contig_30.g3329     GATVAKFTPGKTALILGSGPIGLAMILVLKAKGAKKIVVSEPASIRRELAAKLNVETFDP  239
KA_contig_25.g2491     GSRVAKFTPGKTALVLGAGPIGLAMILVLKAKGAKKIVVSELASIRRELAEKFNVETFDS  239
KS_contig_6.g4029      GSRVANFTPGKTALILGAGPIGLAMILVLKAKGAKKIVVSELASIRRELAERFNVETFDP  239
                       .  :: *. *.:**:**:*****. *****. **.****** *. * *:* ::.**.*:

CAY77584.1             SKHGHKSIEILRGLTKSHDGFDYSYDCSGIQVTFETSLKALTFRGTATNIAVWGPKPVPF  300
KA_contig_13.g329      TKYKNDAVSVLRSIPEGNKGFDFAFDCSGVPSTLNTGVGAIHFRGIHCNVAIWGK-GLDF  298
KA_contig_30.g3329     TKYKNDAVSVLRSIPEGNEGFDFAFDCSGVPSTLNTGVGAIHFRGIHCNVAIWGK-GLDF  298
KA_contig_25.g2491     SKHGDNAISELRAITEGNKGFDFAFDCSGVPPTFNTGIGAIHFRGVYCNVAIWGK-GLNF  298
KS_contig_6.g4029      SKHGDNAISELRSITEGNKGFDFAFDCSGVAPTFNTGIAAIHFRGTYCNVAIWGK-GLNF  298
                       :*: ..::. **.: :.:.***:::****:  *::*.: *: ***   *:*:**   : *

CAY77584.1             QPMDVTLQEKVMTGSIGYVVEDFEEVVRAIHNGDITMEDCKQLITGKQRIEDGWEKGFQE  360
KA_contig_13.g329      NPMDITFQEKVFVGSIGYTIEDFQQVIDAFERKKIDPKECENLITGRQKIEDGWEMGFLE  358
KA_contig_30.g3329     NPMDITFQEKVFVGSIGYTIEDFQQVIDAFERKKIDPKECENLITGRQKIEDGWEKGFLE  358
KA_contig_25.g2491     NPMDITLQEKNLTGSIGYTVEDFKQVVDAFENKKIDPKECEHLITGRQKIEDGWEKGFLE  358
KS_contig_6.g4029      NPMDITLQEKNLTGSIGYTVEDFKQVVDAFEKKKIDPKECEHLITGRQKIEDGWEKGFLE  358
                       :***:*:*** :.*****.:***::*: *:.. .*  ::*::****:*:****** ** *

CAY77584.1             LMDHKESNVKILLTPNNHGEMK- 382
KA_contig_13.g329      LINHKDTNIKVLLTPNTHGELDR 381
KA_contig_30.g3329     LINHKDTNIKVLLTPNTHGELDR 381
KA_contig_25.g2491     LMNHKDTNIKVLLTPNNHGELDG 381
KS_contig_6.g4029      LMNHKDTNIKVLLTPNNHGELDG 381
                       *::**::*:*:*****.***:.
```
